# Isolation, Characterization, and Anti-Allergic Evaluation of Phytochemicals from *Wikstroemia trichotoma*

**DOI:** 10.3390/nu17091552

**Published:** 2025-04-30

**Authors:** Min-Ji Keem, Tae-Young Kim, No-June Park, Sangho Choi, Jin-Hyub Paik, Beom-Geun Jo, Taek-Hwan Kwon, Su-Nam Kim, Seoung Rak Lee, Min Hye Yang

**Affiliations:** 1 Department of Pharmacy, Research Institute for Drug Development, College of Pharmacy, Pusan National University, Busan 46241, Republic of Korea; mj_keem@pusan.ac.kr (M.-J.K.); taeyour@pusan.ac.kr (T.-Y.K.); bg_jo@pusan.ac.kr (B.-G.J.); thkwon@pusan.ac.kr (T.-H.K.); 2 Natural Products Research Institute, Korea Institute of Science and Technology, Gangneung 25451, Republic of Korea; parknojune@kist.re.kr (N.-J.P.); snkim@kist.re.kr (S.-N.K.); 3 International Biological Material Research Center, Korea Research Institute of Bioscience and Biotechnology, Daejeon 34141, Republic of Korea; decoy0@kribb.re.kr (S.C.); jpaik@kribb.re.kr (J.-H.P.); 4 Department of Manufacturing Pharmacy, Research Institute for Drug Development, College of Pharmacy, Pusan National University, Busan 46241, Republic of Korea

**Keywords:** *Wikstroemia trichotoma*, flavonoid, anti-inflammation, anti-allergy, interleukin 4, *β*-hexosaminidase

## Abstract

**Background/Objectives:** The *Wikstroemia* genus has been traditionally used in Asia to treat various ailments, including parotitis, pneumonia, and pertussis. These plants contain many bioactive compounds, including flavonoids, coumarins, and lignans. This study investigates the chemical components of a MeOH extract of the aerial parts of *Wikstroemia trichotoma* (Thunb.) Makino and evaluates their anti-inflammatory and anti-allergic effects in vitro. **Methods:** Chromatographic techniques, spectroscopic analysis, and the literature were used to isolate compounds from the branches and leaves of *W. trichotoma*. IL-4 mRNA and *β*-hexosaminidase levels were assessed by performing assays on RBL-2H3 cells to evaluate anti-inflammatory and anti-allergic potential. **Results:** Forty-two compounds were isolated from the *W. trichotoma* extract, and the flavanones trichotocinol A and B were newly identified. Screening of isolated compounds showed that several significantly inhibited DNP-BSA-induced *β*-hexosaminidase release by 10.0–58.0% and PMA/ionomycin-induced IL-4 mRNA expression by 25.3–71.7% versus negative controls. In addition, trichotocinol A reduced IL-4 mRNA expression by 31.9%. **Conclusions:** The discovery of these new compounds contributes to our understanding of the bioactive properties of *W. trichotoma* and suggests their potential use as natural therapeutic agents for inflammatory disorders.

## 1. Introduction

Allergic inflammation occurs when the immune system exhibits an exaggerated response to allergenic substances. This reaction is closely linked to the main symptoms of allergic diseases, such as rhinitis, eczema, and asthma, which have steadily increased in incidence over the past century [1]. Allergic inflammation is primarily characterized by predominant T helper type 2 (Th2) immune responses and immunoglobulin E (IgE)-mediated mast cell activation [2]. During early inflammatory response, epithelial cells release key cytokines, such as thymic stromal lymphopoietin (TSLP), which amplify Th2-driven allergic inflammation by activating dendritic cells and other immune cells [3]. Subsequently, IgE antibodies generated by activated B cells are released into the bloodstream and bind to FcεRI on mast cells, which are widely present in the skin, respiratory tract, and gastrointestinal system and play a critical role in IgE-mediated allergic reactions [4]. Upon activation, mast cells release preformed mediators, such as histamine and tryptase, through degranulation and synthesize cytokines and chemokines that further exacerbate the inflammatory response [5]. Therefore, effective treatment of allergic inflammatory diseases requires agents that modulate mast cell degranulation and Th2 responses.

*Wikstroemia trichotoma* (Thunb.) Makino is a deciduous broadleaf shrub that grows in East Asia, particularly in Japan and Korea. The plant thrives under trees on islands, coastal forests, valleys, and mountainous regions to reach heights of 0.5 to 2.5 m [6]. The *Wikstroemia* genus has been utilized in traditional Asian medicine for centuries, and the detoxifying, anti-inflammatory, analgesic, and antimicrobial properties of this genus have long been recognized. Accordingly, they have historically been used to treat a range of inflammatory conditions, including bronchitis, arthritis, edema, and cancer [7,8,9]. Furthermore, pharmacological studies have reported that extracts from *Wikstroemia* species possess a variety range of biological effects, including anti-inflammatory, antioxidant, antimicrobial, and immune-modulatory properties [10,11]. Specifically, extracts of *W. trichotoma* have been shown to inhibit inflammatory cytokine secretion and reduce inflammatory responses. Several bioactive phytochemical compounds found in the *Wikstroemia* genus, such as genkwanin, indicanone, and wikstronone A, have potent anti-inflammatory and immune-modulatory effects that suppress inflammation caused by allergic reactions [12,13,14]. However, studies on the anti-inflammatory and immune-modulating activities of compounds produced by these plants are limited.

During our studies to identify novel bioactive compounds in medicinal plants, we investigated a MeOH extract derived from the aerial parts of *W. trichotoma*. Through a combination of successive chromatographic techniques and high-performance liquid chromatography (HPLC), we successfully isolated two new flavonoids (**1** and **2**) along with 40 known compounds (**3**–**42**). The chemical structures of these new flavonoids were elucidated by spectroscopic analysis, including nuclear magnetic resonance (NMR) spectroscopy, high-resolution electrospray ionization mass spectrometry (HR-ESI-MS), and electronic circular dichroism (ECD) spectroscopy. Additionally, we evaluated the biological activities of these isolated compounds by assessing their effects on IL-4 mRNA expression and *β*-hexosaminidase release in RBL-2H3 cells. Herein, we describe the purification, structural elucidation, and biological evaluation of all 42 compounds to expand chemical and pharmacological knowledge of *W. trichotoma* and its potential therapeutic applications.

## 2. Materials and Methods

### 2.1. Plant Material

The *W. trichotoma* samples were collected from Janmi Mountain in Boryeong, Republic of Korea, in August 2022. Samples were confirmed by Dr. Jin-Hyub Paik at the International Biological Material Research Center (IBMRC) of the Korea Research Institute for Bioscience and Biotechnology (KRIBB). The collected plant materials were air-dried at 30 °C before being used in the experiments. A voucher specimen (PNU-0041) has been deposited at the College of Pharmacy, Pusan National University.

### 2.2. Extraction and Isolation

The aerial parts of *W. trichotoma* (3.4 kg) were ground into fine powder, extracted in 34 L of MeOH using ultrasonication for 90 min, and then soaked at room temperature for 12 h. The extract was filtered through a gauze and concentrated under vacuum at 40 °C twice. This process yielded 629.3 g of crude extract (a yield of 18.7%). The crude extract was then suspended in 4 L of distilled water and solvent-partitioned sequentially with 8 L of each of hexane, chloroform (CHCl_3_), ethyl acetate (EtOAc), and *n*-butanol (*n*-BuOH) to provide four fractions containing extracts of 96.2 g, 41.5 g, 216.5 g, and 197.9 g, respectively. Chromatographic techniques suitable for separation were applied to each fraction, resulting in the isolation of 40 known compounds (**3**–**42**). Additionally, two new compounds (**1** and **2**) were isolated, and their structures were confirmed through spectroscopic analysis. The equipment used for analysis and detailed procedures for the isolation of compounds (Figures S1–S3) are described in the Experimental section of the Supplementary Materials.

Trichotocinol A (**1**): pale-yellow amorphous powder; UV (MeOH) λ_max_ (log ε) 225 (4.67), 281 (2.31) and 352 (0.64) nm; ECD (MeOH) λ (Δε) 237 (−1.68), 260 (0.58), and 292 (−2.00) nm; ^1^H (400 MHz) and ^13^C (100 MHz) NMR data, see Table 1; HR-ESI-MS (positive ion-mode) *m*/*z* 703.2532 [M + Na]^+^ (calcd for C_33_H_44_O_15_Na, 703.2573).

Trichotocinol B (**2**): brown amorphous powder; UV (MeOH) λ_max_ (log ε) 222 (3.89), 285 (2.76), and 362 (0.68) nm; ECD (MeOH) λ (Δε) 291 (−4.35) nm; ^1^H (400 MHz) and ^13^C (100 MHz) NMR data, see Table 1; HR-ESI-MS (positive ion-mode) *m*/*z* 779.2047 [M + Na]^+^ (calcd for C_34_H_44_O_19_Na, 779.2359).

### 2.3. Cell Culture

RBL-2H3 cells (KCLB No. 22256) were purchased from the Korea Cell Line Bank (Seoul, Republic of Korea) and cultured in 150 mm dishes at 37 °C in a 5% CO_2_ incubator. The cells were maintained in DMEM (HyClone Laboratories, Logan, UT, USA) supplemented with 10% fetal bovine serum (FBS), 1% penicillin-streptomycin, and 1 mM sodium pyruvate. The cells were passaged when they reached 80–90% confluence, and experiments were conducted using cells from the 20th passage.

### 2.4. IL-4 mRNA Expression in RBL-2H3 Cells

RBL-2H3 cells were pre-treated with isolated compounds for 1 h before induction of inflammation using phorbol 12-myristate 13-acetate (PMA, 50 ng/mL, Sigma-Aldrich, St. Louis, MO, USA) and ionomycin (1 μM, Sigma-Aldrich, St. Louis, MO, USA). The control group was treated with DMSO without PMA/ionomycin. After 20 h of incubation, cells were collected for cDNA synthesis. The expression of IL-4 mRNA was quantified using quantitative real-time PCR (qPCR). Total RNA was extracted using the RNeasy mini kit (Qiagen, Redwood City, CA, USA), and cDNA was synthesized using the RevertAid First Strand cDNA Synthesis Kit (Thermofisher, Waltham, MA, USA). qPCR was carried out on the QuantStudio^™^ 6 Pro Real-Time PCR System (Applied Biosystems, Waltham, MA, USA) and SYBR^®^ Green Master Mix (Applied Biosystems, Waltham, MA, USA). The expression of IL-4 in treated cells was compared to that in the control group using the comparative cycle threshold (Ct) method [15]. The primer sequences used were as follows: IL-4 forward: 5′-CCA CCT TGC TGT CAC CCT GTT CTG CT-3′; IL-4 reverse: 5′-GTG TTG TGA GCG TGG ACT CAT TCA CG-3′; *β*-actin forward: 5′-ACG GTG AAA AGA TGA CCC AG-3′; *β*-actin reverse: 5′- TGT CAG CTG TGG TGG TGA AG-3′. mRNA expressions were normalized to *β*-actin.

### 2.5. β-Hexosaminidase Release in RBL-2H3 Cells

RBL-2H3 cells were cultured overnight in 24-well plates. After sensitization with 100 ng/mL of anti-2,4-dinitrophenylated-IgE (Sigma-Aldrich, St. Louis, MO, USA), cells were incubated for 4 h at 37 °C in a 5% CO_2_. Siraganian buffer (Biosolution, Seoul, Republic of Korea) was used for 2,4-dinitrophenylated-bovine serum albumin (DNP-BSA) and sample treatments. After sensitization with IgE, isolated compounds were applied to the cells at 30 mM for 1 h. Subsequently, the cells were treated with 10 μg/mL DNP-BSA for 30 min. To measure *β*-hexosaminidase activity, the supernatants from the cultures were combined with 10 mM poly-*N*-acetyl glucosamine in 0.1 M sodium citrate buffer (pH 4.5) in a 96-well plate, then incubated for 1 h at 37 °C. *β*-Hexosaminidase activity was quantified by measuring absorbance at 405 nm using the Infinite M1000 microplate reader (Tecan, Männedorf, Switzerland).

### 2.6. Statistical Analysis

Data were analyzed by one-way analysis of variance (ANOVA) and Tukey’s multiple comparisons test using GraphPad Prism 9 (GraphPad Software, San Diego, CA, USA). Results are expressed as means ± standard deviations, and statistical significance was accepted for * *p <* 0.05, ** *p <* 0.01, or *** *p <* 0.001, as indicated.

## 3. Results

### 3.1. Isolation of Compounds from W. trichotoma Extract and the Structural Elucidation of Compounds **1** and **2**

**Figure 1:**
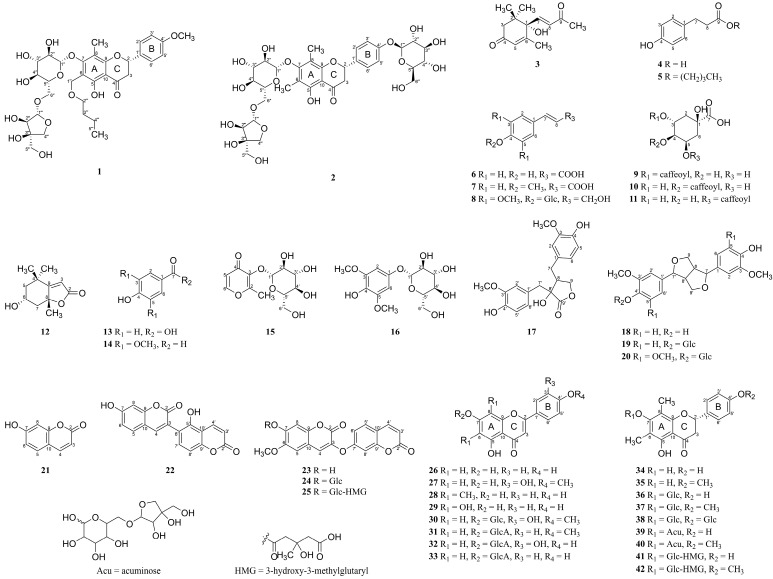
Chemical structures of the isolated compounds (**1**–**42**) from the *W. trichotoma* extract.

Two new flavonoids, seventeen known flavonoids, five coumarins, four lignans, ten phenylpropanoids, two terpenoids, and two simple phenolics were obtained from the MeOH extract of *W. trichotoma* (Figure 1). Known compounds were identified as dehydrovomifoliol (**3**) [16], phloretic acid (**4**) [17], butyl 4-hydroxybenzenepropanoate (**5**) [18], *p*-coumaric acid (**6**) [19], *p*-methoxy coumaric acid (**7**) [20], syringin (**8**) [21], chlorogenic acid (**9**) [22], cryptochlorogenic acid (**10**) [23], neochlorogenic acid (**11**) [24], epiloliolide (**12**) [25], 4-hydroxybenzoic acid (**13**) [26], syringaldehyde (**14**) [27], dianthoside (**15**) [28], koaburaside (**16**) [29], wikstromol (**17**) [30], pinoresinol (**18**) [31], pinoresinol-4′-*O*-glucoside (**19**) [32], syringaresinol-4′-*O*-glucoside (**20**) [33], umbelliferone (**21**) [34], repensin B (**22**) [35], daphnoretin (**23**) [36], daphnorin (**24**) [35], rutarensin (**25**) [37], apigenin (**26**) [38], diosmetin (**27**) [39], syzalterin (**28**) [40], nortangeretin (**29**) [41], diosmetin 7-*O*-glucoside (**30**) [42], acacetin 7-*O*-glucuronide (**31**) [43], luteolin 7-*O*-glucuronide (**32**) [44], apigenin 7-*O*-glucuronide (**33**) [45], farrerol (**34**) [40], matteucinol (**35**) [46], farrerol 7-*O*-glucoside (**36**) [47], matteucinol 7-*O*-glucoside (**37**) [46], diplomorphanin A (**38**) [48], miconioside B (**39**) [49], matteucinol-7-*O*-apiosyl (1 → 6)-glucoside (**40**) [49], matteuinterate B (**41**) [50], and matteuorienate A (**42**) [46] by analyzing their NMR data and comparing it to previously reported values.

Compound **1** was isolated as a pale-yellow amorphous powder. HR-ESI-MS showed an *m*/*z* value of 703.2532 [M + Na]^+^ (calcd. for C_33_H_44_O_15_Na, 703.2573), suggesting a molecular formula of C_33_H_44_O_15_ (Figure S4). The UV spectrum of **1** exhibited maximum absorptions at 225, 281, and 352 nm, implying the presence of a typical flavanone chromophore [51]. The ^1^H NMR data (Table 1) of **1** showed that the existence of three methyl groups [*δ*_H_ 2.04 (3H, s, 8-CH_3_) and 0.87 (3H, t, *J* = 7.5 Hz, H-5⁗)], including one methoxy group [*δ*_H_ 3.78 (3H, s, 4′-OCH_3_)], eight methylene groups [*δ*_H_ 4.59 (1H, d, *J* = 10.0 Hz, H-1⁗a), 4.40 (1H, d, *J* = 10.0 Hz, H-1⁗b), 3.75 (1H, m, H-6″a), 3.73 (1H, d, *J* = 9.0 Hz, H-4‴a), 3.55 (1H, d, *J* = 9.0 Hz, H-4‴b), 3.45 (1H, m, H-6″b), 3.45 (2H, d, *J* = 6.0 Hz, H-2⁗), 3.35 (1H, dd, *J* = 17.0, 13.0 Hz, H-3a), 3.27 (2H, s, H-5‴), 2.84 (1H, dd, *J* = 17.0, 3.0 Hz, H-3b), 1.50 (2H, m, H-3⁗), and 1.36 (2H, m, H-4⁗)], twelve methine protons [*δ*_H_ 7.47 (2H, d, *J* = 8.5 Hz, H-3′ and H-5′), 6.99 (2H, d, *J* = 8.5 Hz, H-2′ and H-6′), 5.59 (1H, dd, *J* = 13.0, 3.0 Hz, H-2), 4.84 (1H, d, *J* = 7.5 Hz, H-1″), 4.77 (1H, d, *J* = 2.5 Hz, H-1‴), 3.68 (1H, m, H-5″), 3.30 (1H, m, H-2″), 3.18 (1H, m, H-2‴), 3.15 (1H, m, H-3″), and 3.15 (1H, m, H-4″)]. Combined analysis of ^13^C NMR (Table 1), HSQC, and HMBC data revealed 33 carbon resonance signals. Fifteen carbon signals observed at *δ*_C_ 198.3 (C-4), 162.5 (C-7), 159.4 (C-4′), 159.3 (C-5), 159.3 (C-9), 130.6 (C-1′), 127.9 (C-2′, C-6′), 113.9 (C-3′, C-5′), 111.3 (C-6), 110.1 (C-8), 104.8 (C-10), 77.9 (C-2), and 42.1 (C-3) were identical to those of flavanone. Additionally, eleven carbon signals were detected at *δ*_C_ 109.2 (C-1‴), 104.6 (C-1″), 78.7 (C-3‴), 76.3 (C-2‴), 75.8 (C-5″), 75.6 (C-3″), 73.9 (C-2″), 73.2 (C-4‴), 69.1 (C-4″), 67.2 (C-6″), and 63.4 (C-5‴), suggesting the presence of an apiofuranosyl (1 → 6)-glucopyranosyl moiety. The remaining seven carbon signals at *δ*_C_ 69.7 (C-2⁗), 60.1 (C-1⁗), 55.1 (4′-OCH_3_), 31.4 (C-3⁗), 19.0 (C-4⁗), 13.8 (C-5⁗), and 9.1 (8-CH_3_) were assigned to substituents attached to the aglycone.

**Figure 2:**
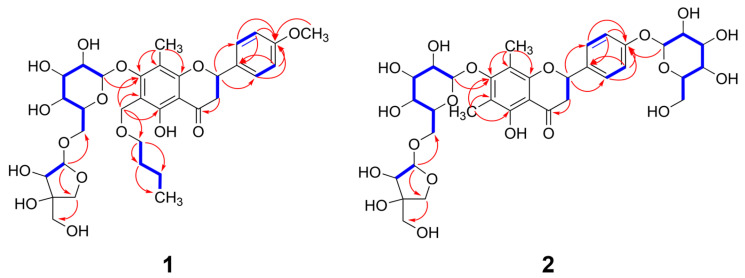
Selected COSY (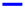) and HMBC (→) correlations of compounds **1** and **2**.

Analysis of 2D NMR spectra (^1^H-^1^H COSY, HSQC, and HMBC) showed **1** had a planar structure (Figures S7–S9). The presence of a flavanone skeleton in **1** was verified by the ^1^H-^1^H COSY correlations of H-2/H_2_-3, H-2′/H-3′, and H-5′/H-6′ and by the HMBC correlations of H-2/C-4, H-2/C-9, H-2/C-2′, and H_2_-3/C-10 (Figure 2). The presence of a methyl and a methoxy group at C-8 and C-4′ of **1**, respectively, was confirmed by the HMBC correlations of 8-CH_3_/C-8 and 4′-OCH_3_/C-4. Detailed interpretation of the remaining 2D NMR correlations suggested that the chemical structure of **1** was similar to that of matteucinol-7-*O*-apiosyl (1 → 6)-glucoside (**40**) and differed only due to the additional substitution of a butoxymethyl group at C-6. ^1^H-^1^H COSY correlations from H_2_-2⁗ to H_3_-5⁗ and HMBC correlations of H_2_-1⁗/C-6 and H_2_-2⁗/C-1⁗ confirmed the presence of a butoxymethyl group attached to C-6 of the aglycone. The ECD spectrum of **1** showed a negative Cotton effect at around 290 nm, indicating C-2 had an *S* configuration (Figure S10), which is commonly observed in typical 2*S*-flavanones known to exhibit a negative Cotton effect at the *π* → π* transition (270–290 nm) [52]. Based on the above evidence, **1** was identified as 5-hydroxy-4′-methoxy-8-methyl-6-(*n*-butoxymethyl)-7-*O*-*β*-d-apiofuranosyl (1 → 6)-*β*-d-glucopyranosyl flavanone and was trivially named trichotocinol A.

Compound **2** was a brown amorphous powder. HR-ESI-MS showed an adducted sodium ion signal at *m/z* 779.2047 [M + Na]^+^ (calcd. for C_34_H_44_O_19_Na, 779.2359), which supported a molecular formula of C_34_H_44_O_19_ (Figure S11). Comprehensive analysis of 1D and 2D NMR spectra of **2** (Figures S12–S16) revealed that its chemical structure was almost identical to that of **40**, except for the absence of a methoxy group at the C-4′ position. The existence of an additional *β*-glucopyranosyl moiety in **2** was established by anomeric signals at *δ*_H_ 4.89 (1H, d, *J* = 7.0 Hz, H-1⁗) and *δ*_C_ 100.3 (C-1⁗), along with one oxymethylene carbon at *δ*_C_ 60.7 (C-6⁗), and four oxymethine carbons at *δ*_C_ 73.2 (C-2⁗), 76.2 (C-3⁗), 69.7 (C-4⁗), and 75.8 (C-5⁗). HMBC correlations of H-2′ and H-6′/C-4′, H-3′ and H-5′/C-4′, as well as H-1⁗/C-4′, indicated the glucose moiety was attached to C-4′ of the B ring (Figure 2). The ECD spectrum of **2** also exhibited a negative Cotton effect near 290 nm corresponding to *π* → π* electronic transitions and confirming that the absolute configuration of C-2 in **2** was *S* (Figure S17). Thus, **2** was identified as a new flavanone glycoside, named trichotocinol B, and assigned the structure 5-hydroxy-4′-*O*-*β*-d-glucopyranosyl-6-methyl-8-methyl-7-*O*-*β*-d-apiofuranosyl (1 → 6)-*β*-d-glucopyranosyl flavanone.

### 3.2. Evaluation of the Effects of Compounds (**1**–**42**) on IL-4 Expression in RBL-2H3 Cells

**Figure 3:**
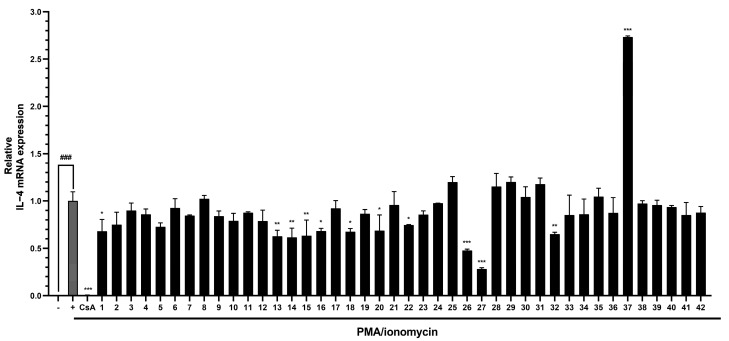
Inhibitory effects of the compounds isolated from the MeOH extract of *W. trichotoma* on PMA/ionomycin-induced IL-4 mRNA expression in RBL-2H3 cells. Data are presented as means ± SDs (*n* = 2), and significance was accepted for ^###^ *p* value < 0.001 versus non-treated cells, and * *p* < 0.05, ** *p* < 0.01, *** *p* < 0.001 versus PMA/ionomycin-treated cells. PMA, phorbol 12-myristate 13-acetate; CsA, cyclosporin A; IL-4, interleukin-4.

PMA/ionomycin treatment significantly increased IL-4 mRNA levels, and several compounds present in the MeOH extract of *W. trichotoma*, viz. simple phenolics, phenylpropanoids, lignans, coumarins, and flavonoids, significantly reduced these increases. Among the simple phenolics, 4-hydroxybenzoic acid (**13**) and syringaldehyde (**14**) significantly reduced PMA/ionomycin-induced IL-4 mRNA levels by 37.2% and 38.5%, respectively. In the phenylpropanoid group, dianthoside (**15**) and koaburaside (**16**) significantly reduced IL-4 mRNA levels by 36.7% and 31.6%. Of the lignans tested, pinoresinol (**18**) and syringaresinol-4′-*O*-glucoside (**20**) exhibited significant inhibitory effects and reduced IL-4 mRNA expression by 32.3% and 31.1%, respectively. Of the five coumarins, repensin B (**22**) significantly reduced IL-4 mRNA expression by 25.3%. In the flavonoid group, specifically, apigenin (**26**), diosmetin (**27**), luteolin 7-*O*-glucuronide (**32**), and trichotocinol A (**1**) significantly decreased PMA/ionomycin-induced IL-4 mRNA levels by 52.3%, 71.7%, 35.0%, and 31.9%, respectively (Figure 3).

### 3.3. Evaluation of the Abilities of Compounds (**1**–**42**) to Suppress β-Hexosaminidase Release by RBL-2H3 Cells

**Figure 4:**
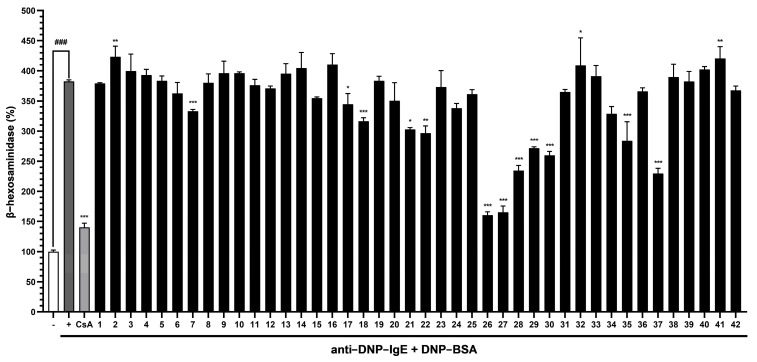
Inhibitory effects of the compounds isolated from *W. trichotoma* on *β*-hexosaminidase release by IgE + DNP-BSA-induced RBL-2H3 cells. Data are presented as means ± SDs (*n* = 2), and significant was accepted for ^###^ *p* values < 0.001 versus non-treated cells, and * *p* < 0.05, ** *p* < 0.01, *** *p* < 0.001 versus IgE + DNP-BSA-treated cells. IgE, immunoglobulin E; DNP-BSA, 2,4-dinitrophenylated-bovine serum albumin; CsA, cyclosporin A.

The inhibitory effects of compounds **1**–**42** were investigated by assessing antigen-induced degranulation in IgE-sensitized RBL-2H3 cells. IgE + DNP-BSA treatment significantly increased *β*-hexosaminidase by 3.8-fold. However, several compounds in the MeOH extract, including phenylpropanoids, lignans, coumarins, and flavonoids, reduced IgE + DNP-BSA-induced *β*-hexosaminidase release. Of the phenylpropanoids, *p*-methoxy coumaric acid (**7**) had a significant inhibitory effect and reduced *β*-hexosaminidase release by 13.0%. The lignans wikstromol (**17**) and pinoresinol (**18**) also demonstrated significant inhibitory effects and reduced *β*-hexosaminidase release by 10.0% and 17.3%, respectively. Regarding the coumarins, umbelliferone (**21**) and repensin B (**22**) significantly inhibited *β*-hexosaminidase release by 20.9% and 22.4%, respectively. In addition, seven flavonoids significantly inhibited *β*-hexosaminidase release. Syzalterin (**28**), matteucinol-7-*O*-glucoside (**37**), and diosmetin-7-*O*-glucoside (**30**) significantly reduced *β*-hexosaminidase release by 38.7%, 39.9%, and 32.1%, respectively. The most potent inhibitors were apigenin (**26**) and diosmetin (**27**), which inhibited *β*-hexosaminidase release by 58.0% and 56.8%, respectively, and reduced *β*-hexosaminidase levels to those of cyclosporin A, the positive control (Figure 4).

## 4. Discussion

Allergic inflammation is a complex immune response characterized by the activation of mast cells and the release of inflammatory mediators such as histamine, *β*-hexosaminidase, and cytokines, including IL-4 [53,54]. These mediators play a central role in the pathogenesis of asthma, allergic rhinitis, atopic dermatitis, and other allergic diseases [55]. We investigated the anti-allergic and anti-inflammatory potentials of phytochemicals produced by *W. trichotoma*, a plant recently shown to alleviate atopic dermatitis-like symptoms [56]. Our findings reveal that *W. trichotoma* contains an array of bioactive compounds, including two newly identified flavonoids (trichotocinol A and B), among which some exhibit significant inhibitory effects on mast cell degranulation and IL-4 expression.

The isolation and characterization of forty-two compounds from *W. trichotoma*, including two novel flavonoids, highlight the diversity of phytochemicals manufactured by this plant. In addition, the structural characterization of these two flavanone glycosides provides valuable insights regarding their potential use for treating allergic inflammation. Glycosylation and the presence of alkyl ether groups in these compounds suggest enhanced membrane permeability and interactions with cellular targets, which could contribute to their observed anti-inflammatory and anti-allergic effects [57,58,59].

Our biological analysis of the isolated compounds showed that flavones, such as apigenin (**26**) and diosmetin (**27**), tended to exhibit more potent inhibitory effects on both IL-4 mRNA expression and *β*-hexosaminidase release than flavanones. These observations suggest that the planar structure and electron delocalization of flavones contribute to their strong anti-allergic effects, whereas the absence of the C2-C3 double bond in flavanones may limit their activity [60]. We also observed that glycosylated flavonoids like matteucinol-7-*O*-glucoside (**37**) significantly inhibited *β*-hexosaminidase release, indicating glycosylation can modulate the biological activity of these compounds by improving their stability or bioavailability [61].

Trichotocinol A (**1**) reduced PMA/ionomycin-induced IL-4 mRNA expression by 31.9%. This finding is particularly noteworthy, as IL-4 is critically involved in Th2 immune response, which involves increasing IgE production and eosinophil recruitment [62]. Structurally, the butoxymethyl group at the C-6 position of **1** might enhance its anti-inflammatory activity by increasing lipophilicity and facilitating interactions with intracellular signaling pathways [63,64]. In contrast, trichotocinol B (**2**), which lacks this substitution, did not significantly inhibit PMA/ionomycin-induced IL-4 expression.

The anti-allergic and anti-inflammatory effects of the isolated compounds are almost certainly mediated by multiple mechanisms. It has been established that flavonoids modulate inflammatory pathways by inhibiting the production of pro-inflammatory cytokines and suppressing the activation of immune cells [65,66,67]. Moreover, the inhibition of *β*-hexosaminidase release and IL-4 expression by flavonoids observed in this study suggests that they may interfere with mast cell signaling pathways, potentially by suppressing the FcεRI-mediated activation or modulating the expression of downstream signaling molecules such as NF-κB and MAPK.

## 5. Conclusions

This study expands our understanding of the chemical constituents and bioactivity of *W. trichotoma*. The identification of trichotocinol A, trichotocinol B, and 40 known compounds in the MeOH extract of *W. trichotoma* provides a foundation for further research on the structure-activity relationships of these compounds. Furthermore, the observed suppressions of mast cell degranulation and IL-4 expression by several compounds highlight their therapeutic potential for treating allergic inflammatory diseases. These compounds may also contribute to the development of functional skincare products, dietary supplements, or pharmaceuticals for managing inflammatory and allergic conditions. In particular, trichotocinol A, which exhibited IL-4 inhibitory effects, may serve as a potential lead compound for novel therapeutic agents, with additional studies. Future studies should aim to clarify the molecular mechanisms driving these effects and evaluate the in vivo efficacy of these compounds in animal models of allergic inflammation.

## Figures and Tables

**Table 1:** ^1^H NMR (400 MHz) and ^13^C NMR (100 MHz) spectral data of **1** and **2** in DMSO-*d*_6_ ^a^.

Position	1		2	
	*δ*_C_	*δ*_H_ (*J* in Hz)	*δ*_C_	*δ*_H_ (*J* in Hz)
2	77.9 d	5.59 dd (13.0/3.0)	77.8 d	5.56 dd (12.5/3.0)
3a	42.1 t	3.35 dd (17.0/13.0)	42.3 t	3.32 m
3b		2.84 dd (17.0/3.0)		2.84 dd (17.0/3.0)
4	198.3 s		198.4 s	
5	159.3 s		157.5 s	
6	111.3 s		111.1 s	
7	162.5 s		161.3 s	
8	110.1 s		110.0 s	
9	159.3 s		157.3 s	
10	104.8 s		104.8 s	
1′	130.6 s		132.1 s	
2′	127.9 d	6.99 d (8.5)	127.8 d	7.46 d (9.0)
3′	113.9 d	7.47 d (8.5)	116.2 d	7.08 d (9.0)
4′	159.4 s		157.9 s	
5′	113.9 d	7.47 d (8.5)	116.2 d	7.08 d (9.0)
6′	127.9 d	6.99 d (8.5)	127.8 d	7.46 d (9.0)
6-CH_3_			8.6 q	2.08 s
8-CH_3_	9.1 q	2.04 s	9.2 q	2.06 s
4′-OCH_3_	55.1 q	3.78 s		
1″	104.6 d	4.84 d (7.5)	104.1 d	4.59 d (7.5)
2″	73.9 d	3.30 m	75.3 d	3.33–3.19
3″	75.6 d	3.15 m	77.0 d	3.33–3.19
4″	69.1 d	3.15 m	69.7 d	3.16 m
5″	75.8 d	3.68 m	76.6 d	3.33–3.19
6″a	67.2 t	3.75 m	67.2 t	3.70 m
6″b		3.45 m		3.43 m
1‴	109.2 d	4.77 d (2.5) 1H	109.2 d	4.76 d (2.5)
2‴	76.3 d	3.18 m	73.7 d	3.33–3.19
3‴	78.7 s		78.8 s	
4‴a	73.2 t	3.73 d (9.0)	73.7 t	3.72 d (9.0)
4‴b		3.55 d (9.0)		3.54 d (9.0)
5‴	63.4 t	3.27 s	63.4 t	3.28 s
1⁗a/1⁗	60.1 t	4.59 d (10.0)	100.3 d	4.89 d (7.0)
1⁗b		4.40 d (10.0)		
2⁗	69.7 t	3.45 d (6.0)	73.2 d	3.33–3.19
3⁗	31.4 t	1.50 m	76.2 d	3.33–3.19
4⁗	19.0 t	1.36 m	69.7 d	3.16 m
5⁗	13.8 q	0.87 t (7.5)	75.8 d	3.33–3.19
6⁗a			60.7 t	3.68 m
6⁗b				3.46 m

^a^ Coupling constants (in parentheses) are in Hz.

## Data Availability

The original contributions presented in the study are included in the article/Supplementary Materials, further inquiries can be directed to the corresponding author.

## Supplementary Materials

The following supporting information can be downloaded at https://www.mdpi.com/article/10.3390/nu17091552/s1, experimental section, Figures S1–S3: Isolation of compounds from the CHCl_3_, EtOAc, and *n*-BuOH fraction of the *W. trichotoma* extract, Figures S4–S10: HR-ESI-MS, 1D and 2D NMR, and ECD data of compound **1**, Figures S11–S17: HR-ESI-MS, 1D and 2D NMR, and ECD data of compound **2**, Figures S18–S57: 1H NMR spectra of compounds **3**–**42**.

## References

[1] Edwards-Salmon S.E., Padmanabhan S.L., Kuruvilla M., Levy J.M. (2022). Increasing Prevalence of Allergic Disease and Its Impact on Current Practice. Curr. Otorhinolaryngol. Rep..

[2] Bellanti J.A. (2024). IgE and Non-IgE Food Allergy: A Review of Immunological Mechanisms. J. Food Allergy.

[3] Ronchese F., Webb G.R., Ochiai S., Lamiable O., Brewerton M. (2024). How Type-2 Dendritic Cells Induce Th2 Differentiation: Instruction, Repression, or Fostering T cell-T Cell Communication?. Allergy.

[4] Dahlin J.S., Maurer M., Metcalfe D.D., Pejler G., Sagi-Eisenberg R., Nilsson G. (2022). The Ingenious Mast Cell: Contemporary Insights into Mast Cell Behavior and Function. Allergy.

[5] Theoharides T.C., Alysandratos K.-D., Angelidou A., Delivanis D.-A., Sismanopoulos N., Zhang B., Asadi S., Vasiadi M., Weng Z., Miniati A. (2012). Mast Cells and Inflammation. Biochim. Biophys. Acta Mol. Basis Dis..

[6] Chen J.-R., Lee S.Y., Guo J.-Q., Jin J.-H., Fan Q., Liao W.-B. (2022). *Wikstroemia fragrans* (Thymelaeaceae, Daphneae), a New Species from Mount Danxia, China Based on Morphological and Molecular Evidence. PhytoKeys.

[7] Liu Z., Dong M., Chang H., Han N., Yin J. (2020). Guaiane Type of Sesquiterpene with NO Inhibitory Activity from the Root of *Wikstroemia Indica*. Bioorg. Chem..

[8] Lu C.-L., Zhu L., Piao J.-H., Jiang J.-G. (2012). Chemical Compositions Extracted from *Wikstroemia indica* and Their Multiple Activities. Pharm. Biol..

[9] Wu M., Su X., Wu Y., Luo Y., Guo Y., Xue Y. (2022). Glycosylated Coumarins, Flavonoids, Lignans and Phenylpropanoids from *Wikstroemia nutans* and Their Biological Activities. Beilstein J. Org. Chem..

[10] Fan Q., Jiang Y.-P., Zhu D.-Q., Xu W., Huang W.-Q., Huang X.-J., Shao M. (2018). Phenols from the Rhizome of *Wikstroemia indica*. Biochem. Syst. Ecol..

[11] Huang W.-H., Zhou G.-X., Wang G.-C., Chung H.-Y., Ye W.-C., Li Y.-L. (2012). A New Biflavonoid with Antiviral Activity from the Roots of *Wikstroemia indica*. J. Asian Nat. Prod. Res..

[12] Liu Y.-Y., Liu Y.-P., Wang X.-P., Qiao Z.-H., Yu X.-M., Zhu Y.-Z., Xie L., Qiang L., Fu Y.-H. (2020). Bioactive Daphnane Diterpenes from *Wikstroemia chuii* with Their Potential Anti-Inflammatory Effects and Anti-HIV Activities. Bioorg. Chem..

[13] Ingert N., Bombarda I., Herbette G., Faure R., Moretti C., Raharivelomanana P. (2013). Oleodaphnoic Acid and Coriaceol, Two New Natural Products from the Stem Bark of *Wikstroemia coriacea*. Molecules.

[14] Lei J.-P., Yuan J.-J., Pi S.-H., Wang R., Tan R., Ma C.-Y., Zhang T., Jiang H.-Z. (2017). Flavones and Lignans from the Stems of *Wikstroemia scytophylla* Diels. Pharmacogn. Mag..

[15] Schmittgen T.D., Livak K.J. (2008). Analyzing Real-Time PCR Data by the Comparative CT Method. Nat. Protoc..

[16] Yang Y., Bakri M., Gu D., Aisa H.A. (2013). Separation of (S)-Dehydrovomifoliol from Leaves of *Nitraria sibirica* pall. by High-Speed Counter-Current Chromatography. J. Liq. Chromatogr. Relat. Technol..

[17] Owen R.W., Haubner R., Mier W., Giacosa A., Hull W.E., Spiegelhalder B., Bartsch H. (2003). Isolation, Structure Elucidation and Antioxidant Potential of the Major Phenolic and Flavonoid Compounds in Brined Olive Drupes. Food Chem. Toxicol..

[18] Botta G., Bizzarri B.M., Garozzo A., Timpanaro R., Bisignano B., Amatore D., Palamara A.T., Nencioni L., Saladino R. (2015). Carbon Nanotubes Supported Tyrosinase in the Synthesis of Lipophilic Hydroxytyrosol and Dihydrocaffeoyl Catechols with Antiviral Activity against DNA and RNA Viruses. Bioorg. Med. Chem..

[19] Bertelli D., Papotti G., Bortolotti L., Marcazzan G.L., Plessi M. (2012). ^1^H-NMR Simultaneous Identification of Health-Relevant Compounds in Propolis Extracts. Phytochem. Anal..

[20] Tang K.S.C., Konczak I., Zhao J. (2017). Phenolic Compounds of the Australian Native Herb *Prostanthera rotundifolia* and Their Biological Activities. Food Chem..

[21] Yang E.-J., Kim S.-I., Ku H.-Y., Lee D.-S., Lee J.-W., Kim Y.-S., Seong Y.-H., Song K.-S. (2010). Syringin from Stem Bark of *Fraxinus rhynchophylla* Protects Aβ(25–35)-Induced Toxicity in Neuronal Cells. Arch. Pharm. Res..

[22] Han T., Li H., Zhang Q., Zheng H., Qin L. (2006). New Thiazinediones and Other Components from *Xanthium strumarium*. Chem. Nat. Compd..

[23] Chan E.W., Wong S., Lim Y., Ling S. (2014). Caffeoylquinic Acids in Leaves of Selected Apocynaceae Species: Their Isolation and Content. Phcog. Res..

[24] Forino M., Tenore G.C., Tartaglione L., Carmela D., Novellino E., Ciminiello P. (2015). (1S,3R,4S,5R)5-O-Caffeoylquinic Acid: Isolation, Stereo-Structure Characterization and Biological Activity. Food Chem..

[25] Chi H., Qi X., Wang X., Wang Y., Han X., Wang J., Wang H. (2021). Preparative Separation and Purification of Loliolide and Epiloliolide from *Ascophyllum nodosum* Using Amine-Based Microporous Organic Polymer for Solid Phase Extraction Coupled with Macroporous Resin and Prep-HPLC. Anal. Methods.

[26] Cho J.-Y., Moon J.-H., Seong K.-Y., Park K.-H. (1998). Antimicrobial Activity of 4-Hydroxybenzoic Acid and *Trans* 4-Hydroxycinnamic Acid Isolated and Identified from Rice Hull. Biosci. Biotechnol. Biochem..

[27] Kim H., Ralph J., Lu F., Ralph S.A., Boudet A.-M., MacKay J.J., Sederoff R.R., Ito T., Kawai S., Ohashi H. (2003). NMR Analysis of Lignins in CAD-Deficient Plants. Part 1. Incorporation of Hydroxycinnamaldehydes and Hydroxybenzaldehydes into Lignins. Org. Biomol. Chem..

[28] Plouvier V., Martin M.-T., Brouard J.-P. (1993). Two Pyran Type Glycosides from *Tunica prolifera*. Phytochemistry.

[29] Shao J.-H., Chen J., Zhao C.-C., Shen J., Liu W.-Y., Gu W.-Y., Li K.-H. (2019). Insecticidal and *α*-Glucosidase Inhibitory Activities of Chemical Constituents from *Viburnum Fordiae* Hance. Nat. Prod. Res..

[30] Sefkow M. (2001). Enantioselective Synthesis of (−)-Wikstromol Using a New Approach via Malic Acid. J. Org. Chem..

[31] Brenes M., Hidalgo F.J., García A., Rios J.J., García P., Zamora R., Garrido A. (2000). Pinoresinol and 1-acetoxypinoresinol, Two New Phenolic Compounds Identified in Olive Oil. J. Americ. Oil Chem. Soc..

[32] Ouyang M.-A., Wein Y.-S., Zhang Z.-K., Kuo Y.-H. (2007). Inhibitory Activity against Tobacco Mosaic Virus (TMV) Replication of Pinoresinol and Syringaresinol Lignans and Their Glycosides from the Root of *Rhus javanica* Var. roxburghiana. J. Agric. Food Chem..

[33] Park J.-H., Jung Y.-J., Jung J.-W., Shrestha S., Lim D.W., Han D., Baek N.-I. (2014). A New Flavonoid Glycoside from the Root Bark of *Morus alba* L. Nat. Prod. Res..

[34] Singh R., Singh B., Singh S., Kumar N., Kumar S., Arora S. (2010). Umbelliferone—An Antioxidant Isolated from *Acacia nilotica* (L.) Willd. Ex. Del. Food Chem..

[35] Kicel A., Wolbis M. (2012). Coumarins from the Flowers of Trifolium Repens. Chem. Nat. Compd..

[36] Li X.-N., Tong S.-Q., Cheng D.-P., Li Q.-Y., Yan J.-Z. (2014). Coumarins from *Edgeworthia chrysantha*. Molecules.

[37] Yang Q.-Y., Tian X.-Y., Fang W.-S. (2007). Bioactive Coumarins from *Boenninghausenia sessilicarpa*. J. Asian Nat. Prod. Res..

[38] Zhang Y., Shi S., Wang Y., Huang K. (2011). Target-Guided Isolation and Purification of Antioxidants from *Selaginella sinensis* by Offline Coupling of DPPH-HPLC and HSCCC Experiments. J. Chromatogr. B.

[39] Park Y., Moon B., Yang H., Lee Y., Lee E., Lim Y. (2007). Complete Assignments of NMR Data of 13 Hydroxymethoxyflavones. Magn. Reson. Chem..

[40] Youssef D.T.A., Ramadan M.A., Khalifa A.A. (1998). Acetophenones, a Chalcone, a Chromone and Flavonoids from *Pancratium maritimum*. Phytochemistry.

[41] Ko J.-H., Nam Y.H., Joo S.-W., Kim H.-G., Lee Y.-G., Kang T.H., Baek N.-I. (2018). Flavonoid 8-O-Glucuronides from the Aerial Parts of Malva Verticillata and Their Recovery Effects on Alloxan-Induced Pancreatic Islets in Zebrafish. Molecules.

[42] Li A., Sun A., Liu R., Zhang Y., Cui J. (2014). An Efficient Preparative Procedure for Main Flavonoids from the Peel of *Trichosanthes kirilowii* Maxim. Using Polyamide Resin Followed by Semi-Preparative High Performance Liquid Chromatography. J. Chromatogr. B.

[43] Lee J.Y., Chang E.J., Kim H.J., Park J.H., Choi S.W. (2002). Antioxidative Flavonoids from Leaves of *Carthamus tinctorius*. Arch. Pharm. Res..

[44] Ringl A., Prinz S., Huefner A., Kurzmann M., Kopp B. (2007). Chemosystematic Value of Flavonoids from *Crataegus x Macrocarpa* (Rosaceae) with Special Emphasis on (*R*)- and (*S*)-Eriodictyol-7-*O*-glucuronide and Luteolin-7-*O*-glucuronide. Chem. Biodivers..

[45] Feng J.H., Lee H.J., Kim S.B., Jung J.S., Lim S.S., Suh H.W. (2019). Antinociceptive Effect of Single Components Isolated from *Agrimonia pilosa* Ledeb. Extract. Sci. Pharm..

[46] Huh J., Ha T.K.Q., Kang K.B., Kim K.H., Oh W.K., Kim J., Sung S.H. (2017). *C* -Methylated Flavonoid Glycosides from *Pentarhizidium orientale* Rhizomes and Their Inhibitory Effects on the H1N1 Influenza Virus. J. Nat. Prod..

[47] Lai Y., Zeng H., He M., Qian H., Wu Z., Luo Z., Xue Y., Yao G., Zhang Y. (2016). 6,8-Di-C-Methyl-Flavonoids with Neuroprotective Activities from *Rhododendron fortunei*. Fitoterapia.

[48] Devkota H.P., Watanabe M., Watanabe T., Yahara S. (2013). Diplomorphanins A and B: New *C*-Methyl Flavonoids from *Diplomorpha canescens*. Chem. Pharm. Bull..

[49] Gimenez V.M.M., e Silva M.L.A., Cunha W.R., Januario A.H., Costa E.J.X., Pauletti P.M. (2020). Influence of Environmental, Geographic, and Seasonal Variations in the Chemical Composition of *Miconia* Species from Cerrado. Biochem. Syst. Ecol..

[50] Li X., Zhu L.-J., Chen J.-P., Shi C.-Y., Niu L.-T., Zhang X., Yao X.-S. (2019). C-Methylated Flavanones from the Rhizomes of *Matteuccia intermedia* and Their α-Glucosidase Inhibitory Activity. Fitoterapia.

[51] Tošović J., Marković S. (2017). Reproduction and Interpretation of the UV–Vis Spectra of Some Flavonoids. Chem. Pap..

[52] Slade D., Ferreira D., Marais J.P.J. (2005). Circular Dichroism, a Powerful Tool for the Assessment of Absolute Configuration of Flavonoids. Phytochemistry.

[53] Lecce M., Molfetta R., Milito N.D., Santoni A., Paolini R. (2020). FcεRI Signaling in the Modulation of Allergic Response: Role of Mast Cell-Derived Exosomes. Int. J. Mol. Sci..

[54] Theoharides T.C., Kalogeromitros D. (2006). The Critical Role of Mast Cells in Allergy and Inflammation. Ann. N. Y. Acad. Sci..

[55] Zou F., Du Q., Zhang Y., Zuo L., Sun Z. (2023). Pseudo-Allergic Reactions Induced by Chinese Medicine Injections: A Review. Chin. Med..

[56] Keem M.-J., Jo B.-G., Lee S.H., Kim T.-Y., Jung Y.S., Jeong E.-J., Kim K.H., Kim S.-N., Yang M.H. (2024). Ameliorative Effects of *Wikstroemia trichotoma* 95% EtOH Extract on a Mouse Model of DNCB-Induced Atopic Dermatitis. J. Ethnopharmacol..

[57] Alizadeh S.R., Ebrahimzadeh M.A. (2022). O-Substituted Quercetin Derivatives: Structural Classification, Drug Design, Development, and Biological Activities, a Review. J. Mol. Struct..

[58] Cen-Pacheco F., Ortiz-Celiseo A., Peniche-Cardeña A., Bravo-Ruiz O., López-Fentanes F.C., Valerio-Alfaro G., Fernández J.J. (2020). Studies on the Bioactive Flavonoids Isolated from *Azadirachta indica*. Nat. Prod. Res..

[59] Gębalski J., Graczyk F., Załuski D. (2022). Paving the Way towards Effective Plant-Based Inhibitors of Hyaluronidase and Tyrosinase: A Critical Review on a Structure–Activity Relationship. J. Enzyme Inhib. Med. Chem..

[60] Kim H.P., Son K.H., Chang H.W., Kang S.S. (2004). Anti-Inflammatory Plant Flavonoids and Cellular Action Mechanisms. J. Pharmacol. Sci..

[61] Hollman P.C.H., Bijsman M.N.C.P., Van Gameren Y., Cnossen E.P.J., De Vries J.H.M., Katan M.B. (1999). The Sugar Moiety Is a Major Determinant of the Absorption of Dietary Flavonoid Glycosides in Man. Free Radic. Res..

[62] Keegan A.D., Leonard W.J., Zhu J. (2021). Recent Advances in Understanding the Role of IL-4 Signaling. Fac. Rev..

[63] Huang S.-T., Lee Y., Gullen E.A., Cheng Y.-C. (2008). Impacts of Baicalein Analogs with Modification of the 6th Position of A Ring on the Activity toward NF-κB, AP-1 or CREB Mediated Transcription. Bioorg. Med. Chem. Lett..

[64] Shamsudin N.F., Ahmed Q.U., Mahmood S., Shah S.A.A., Sarian M.N., Khattak M.M.A.K., Khatib A., Sabere A.S.M., Yusoff Y.M., Latip J. (2022). Flavonoids as Antidiabetic and Anti-Inflammatory Agents: A Review on Structural Activity Relationship-Based Studies and Meta-Analysis. Int. J. Mol. Sci..

[65] Chen L., Teng H., Xie Z., Cao H., Cheang W.S., Skalicka-Woniak K., Georgiev M.I., Xiao J. (2018). Modifications of Dietary Flavonoids towards Improved Bioactivity: An Update on Structure–Activity Relationship. Crit. Rev. Food Sci. Nutr..

[66] Kumar S., Pandey A.K. (2013). Chemistry and Biological Activities of Flavonoids: An Overview. Sci. World J..

[67] Martínez G., Mijares M.R., De Sanctis J.B. (2019). Effects of Flavonoids and Its Derivatives on Immune Cell Responses. Recent Pat. Inflamm. Allergy Drug Discov..

