# Supplementary material for: Isolation, Characterization, and Anti-Allergic Evaluation of Phytochemicals from Wikstroemia trichotoma
Source: Nutrients. 2025 Apr 30;17(9):1552. doi: 10.3390/nu17091552 (PMC12073274; doi:10.3390/nu17091552)

SUPPLEMENTARY MATERIALS

Isolation, Characterization, and Anti-allergic Evaluation of phytochemicals from *Wikstroemia trichotoma*

Min-Ji Keem [1,†], Tae-Young Kim [1,†], No-June Park [2], SangHo Choi [3], Jin-Hyub Paik [3], Beom-Geun Jo [1], Taek-Hwan Kwon [1], Su-Nam Kim [2], Seoung Rak Lee [4,*], Min Hye Yang [1,*]

[1] Department of Pharmacy, College of Pharmacy and Research Institute for Drug Development, Pusan National University, Busan 46241, Republic of Korea
[2] Natural Products Research Institute, Korea Institute of Science and Technology, Gangneung 25451, Republic of Korea
[3] International Biological Material Research Center, Korea Research Institute of Bioscience and Biotechnology, Daejeon 34141, Republic of Korea
[4] Department of Manufacturing Pharmacy, College of Pharmacy and Research Institute for Drug Development, Pusan National University, Busan 46241, Republic of Korea
* Correspondence: srlee17@pusan.ac.kr (S.R.L.); mhyang@pusan.ac.kr (M.H.Y.); Tel.: +82-51-510-2803 (S.R.L.); +82-51-510-2811 (M.H.Y.); Fax: +82-33-650-3419 (S.-N.K.); +82-51-513-6754 (M.H.Y.)

Contents of Supplementary Materials

1. Experimental ···· S5

1.1. General Experimental Procedures ···· S5

1.2. Isolation and Purification of Compounds from $CHCl_3$ Fraction ···· S6

1.3. Isolation and Purification of Compounds from EtOAc Fraction ···· S8

1.4. Isolation and Purification of Compounds from *n*-BuOH Fraction ···· S10

List of Figures

Figure S1. Isolation of compounds from the $CHCl_3$ fraction of the *W. trichotoma* extract ···· S7

Figure S2. Isolation of compounds from the EtOAc fraction of the *W. trichotoma* extract ···· S9

Figure S3. Isolation of compounds from the *n*-BuOH fraction of the *W. trichotoma* extract ···· S12

Figure S4. The HR-ESI-MS data of compound **1** ···· S13

Figure S5. The ^{1}H NMR spectrum of compound **1** (DMSO-d_6, 400 MHz) ···· S14

Figure S6. The ^{13}C NMR spectrum of compound **1** (DMSO-d_6, 100 MHz) ···· S15

Figure S7. The ^{1}H-^{1}H COSY spectrum of compound **1** (DMSO-d_6) ···· S16

Figure S8. The HSQC spectrum of compound **1** (DMSO-d_6) ···· S17

Figure S9. The HMBC spectrum of compound **1** (DMSO-d_6) ···· S18

Figure S10. The ECD spectrum of compound **1** ···· S19

Figure S11. The HR-ESI-MS data of compound **2** ···· S20

Figure S12. The ^{1}H NMR spectrum of compound **2** (DMSO-d_6, 400 MHz) ···· S21

Figure S13. The ^{13}C NMR spectrum of compound **2** (DMSO-d_6, 100 MHz) ···· S22

Figure S14. The ^{1}H-^{1}H COSY spectrum of compound **2** (DMSO-d_6) ···· S23

Figure S15. The HSQC spectrum of compound **2** (DMSO-d_6) ··· S24

Figure S16. The HMBC spectrum of compound **2** (DMSO-d_6) ··· S25

Figure S17. The ECD spectrum of compound **2** ··· S26

Figure S18. The ^{1}H NMR spectrum of compound **3** (DMSO-d_6, 400 MHz) ··· S27

Figure S19. The ^{1}H NMR spectrum of compound **4** (DMSO-d_6, 400 MHz) ··· S28

Figure S20. The ^{1}H NMR spectrum of compound **5** (DMSO-d_6, 400 MHz) ··· S29

Figure S21. The ^{1}H NMR spectrum of compound **6** (DMSO-d_6, 400 MHz) ··· S30

Figure S22. The ^{1}H NMR spectrum of compound **7** (DMSO-d_6, 400 MHz) ··· S31

Figure S23. The ^{1}H NMR spectrum of compound **8** (DMSO-d_6, 400 MHz) ··· S32

Figure S24. The ^{1}H NMR spectrum of compound **9** (DMSO-d_6, 400 MHz) ··· S33

Figure S25. The ^{1}H NMR spectrum of compound **10** (DMSO-d_6, 400 MHz) ··· S34

Figure S26. The ^{1}H NMR spectrum of compound **11** (DMSO-d_6, 400 MHz) ··· S35

Figure S27. The ^{1}H NMR spectrum of compound **12** (DMSO-d_6, 400 MHz) ··· S36

Figure S28. The ^{1}H NMR spectrum of compound **13** (DMSO-d_6, 400 MHz) ··· S37

Figure S29. The ^{1}H NMR spectrum of compound **14** (DMSO-d_6, 400 MHz) ··· S38

Figure S30. The ^{1}H NMR spectrum of compound **15** (DMSO-d_6, 400 MHz) ··· S39

Figure S31. The ^{1}H NMR spectrum of compound **16** (DMSO-d_6, 400 MHz) ··· S40

Figure S32. The ^{1}H NMR spectrum of compound **17** (DMSO-d_6, 400 MHz) ··· S41

Figure S33. The ^{1}H NMR spectrum of compound **18** (DMSO-d_6, 400 MHz) ··· S42

Figure S34. The ^{1}H NMR spectrum of compound **19** (DMSO-d_6, 400 MHz) ··· S43

Figure S35. The ^{1}H NMR spectrum of compound **20** (DMSO-d_6, 400 MHz) ··· S44

Figure S36. The ^{1}H NMR spectrum of compound **21** (DMSO-d_6, 400 MHz) ··· S45

Figure S37. The ^{1}H NMR spectrum of compound **22** (DMSO-d_6, 400 MHz) ········ S46
Figure S38. The ^{1}H NMR spectrum of compound **23** (Acetone-d_6, 400 MHz) ········ S47
Figure S39. The ^{1}H NMR spectrum of compound **24** (DMSO-d_6, 400 MHz) ········ S48
Figure S40. The ^{1}H NMR spectrum of compound **25** (DMSO-d_6, 400 MHz) ········ S49
Figure S41. The ^{1}H NMR spectrum of compound **26** (DMSO-d_6, 400 MHz) ········ S50
Figure S42. The ^{1}H NMR spectrum of compound **27** (DMSO-d_6, 400 MHz) ········ S51
Figure S43. The ^{1}H NMR spectrum of compound **28** (DMSO-d_6, 400 MHz) ········ S52
Figure S44. The ^{1}H NMR spectrum of compound **29** (DMSO-d_6, 400 MHz) ········ S53
Figure S45. The ^{1}H NMR spectrum of compound **30** (DMSO-d_6, 400 MHz) ········ S54
Figure S46. The ^{1}H NMR spectrum of compound **31** (DMSO-d_6, 400 MHz) ········ S55
Figure S47. The ^{1}H NMR spectrum of compound **32** (DMSO-d_6, 400 MHz) ········ S56
Figure S48. The ^{1}H NMR spectrum of compound **33** (DMSO-d_6, 400 MHz) ········ S57
Figure S49. The ^{1}H NMR spectrum of compound **34** (DMSO-d_6, 400 MHz) ········ S58
Figure S50. The ^{1}H NMR spectrum of compound **35** (DMSO-d_6, 400 MHz) ········ S59
Figure S51. The ^{1}H NMR spectrum of compound **36** (DMSO-d_6, 400 MHz) ········ S60
Figure S52. The ^{1}H NMR spectrum of compound **37** (DMSO-d_6, 400 MHz) ········ S61
Figure S53. The ^{1}H NMR spectrum of compound **38** (DMSO-d_6, 400 MHz) ········ S62
Figure S54. The ^{1}H NMR spectrum of compound **39** (DMSO-d_6, 400 MHz) ········ S63
Figure S55. The ^{1}H NMR spectrum of compound **40** (DMSO-d_6, 400 MHz) ········ S64
Figure S56. The ^{1}H NMR spectrum of compound **41** (DMSO-d_6, 400 MHz) ········ S65
Figure S57. The ^{1}H NMR spectrum of compound **42** (DMSO-d_6, 400 MHz) ········ S66

1. Experimental

1.1. General Experimental Procedures

NMR spectra were acquired on a JNM-ECZ 400S instrument (JEOL, Tokyo, Japan) using acetone-d_6 and dimethyl sulfoxide (DMSO-d_6) as solvents. ECD measurements were carried out with a J-1500 CD spectrometer (JASCO, Tokyo, Japan), and LC-MS was performed on a 1290 Infinity II ultra-HPLC system (Agilent Technologies, CA, USA) coupled to ZenoTOF 7600 mass spectrometer (SCIEX, MA, USA). HPLC was per-formed using an INNO C18 column (10.0 mm × 250 mm I.D., 10 µm, Youngjin Biochem, Seongnam, Republic of Korea), an LC-20AT pump (Shimadzu, Kyoto, Japan), and a SPD-20A UV/vis detector (Shimadzu, Kyoto, Japan) or a Gilson HPLC system (GILSON Inc., Wisconsin, USA) equipped with 305/307 pumps, an 811C dynamic mixer, and a Watchers 120 ODS-BP column (10.0 mm × 250 mm I.D., 10 µm, Isu Industry Corp., Seoul, Republic of Korea). For column chromatography, Silica gel 60 (Merck, Darmstadt, Germany) and Sephadex™ LH-20 (25–100 µm particle size, GE Healthcare, IL, USA) was used as the resin. Thin-layer chromatography was performed using silica gel 60 F254 plates (0.25 mm, Merck, Darmstadt, Germany), and developed spots were visualized under UV light using sulfuric *p*-anisaldehyde as the detection reagent.

1.2. Isolation and Purification of Compounds from $CHCl_3$ Fraction

The $CHCl_3$ fraction (41.5 g) of *W. trichotoma* was subjected to silica gel column chromatography (CC) and fractionated with hexane-EtOAc (3:1–0:1) to obtain nine fractions (WTC1 ~ WTC9). Fraction WTC4 (940.3 mg) was recrystallized from MeOH to obtain compound **35** (717.9 mg). Fraction WTC5 (6.7 g) was also recrystallized from MeOH and separated by silica gel CC (hexane-EtOAc, 3:1–0:1) to yield compound **34** (2.0 g). Fraction WTC6 (4.4 g) was separated by silica gel CC (hexane-EtOAc, 5:1–0:1) into nine subfractions (WTC6-1 ~ WTC6-9). Subfraction WTC6-3 (937.3 mg) was further separated by silica gel CC (hexane-EtOAc, 2:1–0:1) into four subfractions (WTC6-3-1 ~ WTC6-3-4). Subfraction WTC6-3-4 (96.2 mg) was purified by semi-prep HPLC (Shimadzu system, UV wavelength at 254 and 330 nm, 2 mL/min, Watchers 120 ODS-BP column, 10 μm, 10.0 mm × 250 mm) using 0.1% formic acid in CH_3CN-0.1% formic acid in H_2O (18:82) to obtain compound **6** (t_R = 20.1 min, 0.9 mg). Fraction WTC7 (4.8 g) was separated by silica gel CC using CH_2Cl_2-MeOH (40:1–0:1) as eluent to obtain five subfractions (WTC7-1 ~ WTC7-5). Subfraction WTC7-2 (3.5 g) was recrystallized from MeOH to obtain compound **23** (22.8 mg), and the remainder of the WTC7-2 supernatant was subjected to silica gel CC with hexane-EtOAc (5:1–0:1) to yield eight subfractions (WTC7-2-1 ~ WTC7-2-8). Subfraction WTC7-2-3 (123.6 mg) was further separated into five subfractions (WTC7-2-3-1 ~ WTC7-2-3-5) using Sephadex LH-20 CC with MeOH. Subfraction WTC7-2-3-3 (56.4 mg) was separated by semi-prep HPLC (Shimadzu system, UV wavelength at 254 and 330 nm, 2 mL/min, Watchers 120 ODS-BP column, 10 μm, 10.0 mm × 250 mm) using 0.1% formic acid in CH_3CN-0.1% formic acid in H_2O (22:78) as eluent to yield compounds **14** (t_R = 25.4 min, 3.0 mg), **12** (t_R = 31.5 min, 18.9 mg), and **3** (t_R = 65.6 min, 1.5 mg). Subfraction WTC7-2-5 (296.6 mg) was divided into four subfractions (WTC7-2-5-1 ~ WTC7-2-5-4) using Sephadex LH-20 CC and acetone as eluent. Subfraction WTC7-2-5-2 (200.9 mg) was further separated by silica gel CC (hexane-EtOAc, 1:2–0:1) to yield six subfractions (WTC7-2-5-2-1 ~ WTC7-2-5-2-6), which included compounds **18** (30.2 mg) and **17** (8.9 mg). Subfraction WTC7-2-8 (211.0 mg) was separated using silica gel CC (hexane-EtOAc, 3:1–0:1) to yield four subfractions (WTC7-2-8-1 ~ WTC7-2-8-4) and compound **16** (t_R = 10.8 min, 1.4 mg) was identified in WTC7-2-8-1 (60.3 mg).

Figure S1. Isolation of compounds from the $CHCl_3$ fraction of the *W. trichotoma* extract.

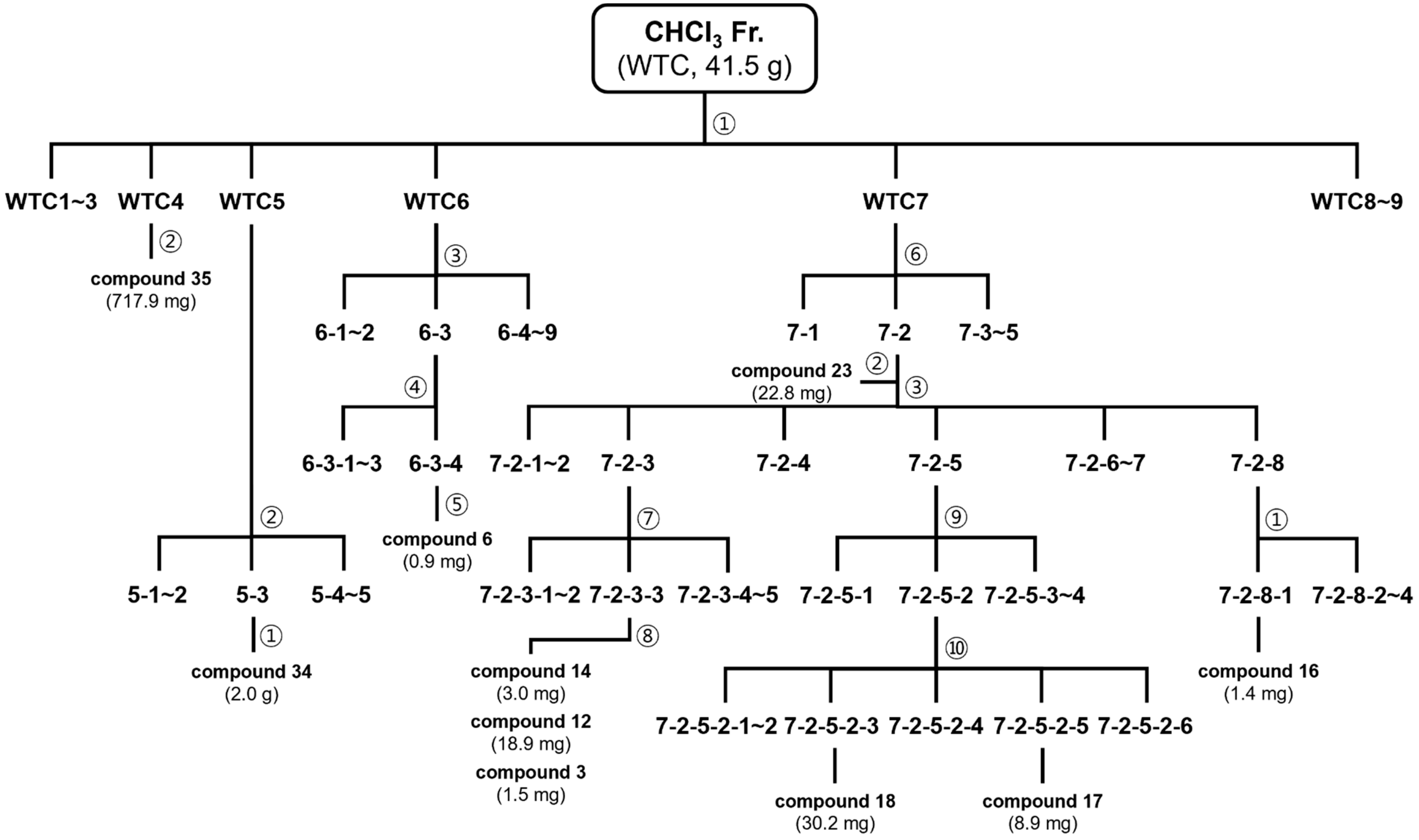

① Silica gel CC (hexane:EtOAc = 3:1 → 0:1)
② Recrystallization (MeOH)
③ Silica gel CC (hexane:EtOAc = 5:1 → 0:1)
④ Silica gel CC (hexane:EtOAc = 2:1 → 0:1)
⑤ semi-prep HPLC (0.1% formic acid in CH_3CN: 0.1% formic acid in H_2O = 18:82)
⑥ Silica gel CC (CH_2Cl_2:MeOH = 40:1 → 0:1)
⑦ Sephadex LH-20 (100% MeOH)
⑧ semi-prep HPLC (0.1% formic acid in CH_3CN: 0.1% formic acid in H_2O = 22:78)
⑨ Sephadex LH-20 (100% Acetone)
⑩ Silica gel CC (hexane:EtOAc = 1:2 → 0:1)

1.3. Isolation and Purification of Compounds from EtOAc Fraction

The EtOAc fraction (186.8 g) was recrystallized from MeOH and purified by semi-prep HPLC (Shimadzu system, UV wavelength at 254 and 330 nm, 2 mL/min, Watchers 120 ODS-BP column, 10 µm, 10.0 mm × 250 mm) using 0.1% formic acid in CH_3CN-0.1% formic acid in H_2O (35:65) as eluent to yield compounds **39** (t_R = 11.2 min, 90.3 mg) and **42** (t_R = 46.5 min, 13.5 mg). The remaining fraction was fractionated by silica gel CC using CH_2Cl_2-MeOH (7:1–0:1) to obtain six fractions (WTE1 ~ WTE6). Fraction WTE1 (1.5 g) was further fractionated by silica gel CC with hexane-EtOAc (1:2–0:1) into seven subfractions (WTE1-1 ~ WTE1-7). Subfraction WTE1-2 (48.1 mg) was processed by prep-TLC with hexane-EtOAc (1:1) and yielded five subfractions (WTE1-2-1 ~ WTE1-2-5). Compound **21** (R_f = 0.63, 26.1 mg) was identified in WTE 1-2-4, and compounds **26** (t_R = 11.2 min, 3.3 mg), **27** (t_R = 16.7 min, 0.4 mg), **29** (t_R = 25.1 min, 0.4 mg), and **28** (t_R = 30.3 min, 1.6 mg) were isolated from WTE1-2-5 (11.1 mg) by semi-prep HPLC (Shimadzu system, UV wavelength at 254 and 330 nm, 2 mL/min, Watchers 120 ODS-BP column, 10 µm, 10.0 mm × 250 mm) using 0.1% formic acid in CH_3CN-0.1% formic acid in H_2O (45:55) as eluent. Subfraction WTE1-3 (57.2 mg) was recrystallized from MeOH to obtain compound **22** (4.8 mg). Fraction WTE3 (31.6 g) was separated by silica gel CC using EtOAc-MeOH (10:1–0:1) into five subfractions (WTE3-1 ~ WTE3-5). Subfraction WTE3-1 (1.2 g) was processed by Sephadex LH-20 CC with MeOH and yielded six subfractions (WTE3-1-1 ~ WTE3-1-6). Subfraction WTE3-1-2 (257.0 mg) was purified by semi-prep HPLC (Shimadzu system, UV wavelength at 254 and 340 nm, 2 mL/min, Watchers 120 ODS-BP column, 10 µm, 10.0 mm × 250 mm) using 0.1% formic acid in CH_3CN-0.1% formic acid in H_2O (35:65) as eluent to obtain compounds **36** (t_R = 14.6 min, 11.6 mg) and **13** (t_R = 15.0 min, 2.2 mg). Subfraction WTE3-2 (8.0 g) was recrystallized from MeOH, yielding compound **40** (3.2 g). Fraction WTE4 (21.9 g) was separated by silica gel CC with EtOAc-MeOH (10:1–0:1), yielding eight subfractions (WTE4-1 ~ WTE4-8). Subfraction WTE4-6 (894.3 mg) was separated by Sephadex LH-20 CC using MeOH to isolate compound **41** (5.5 mg).

Figure S2. Isolation of compounds from the EtOAc fraction of the *W. trichotoma* extract.

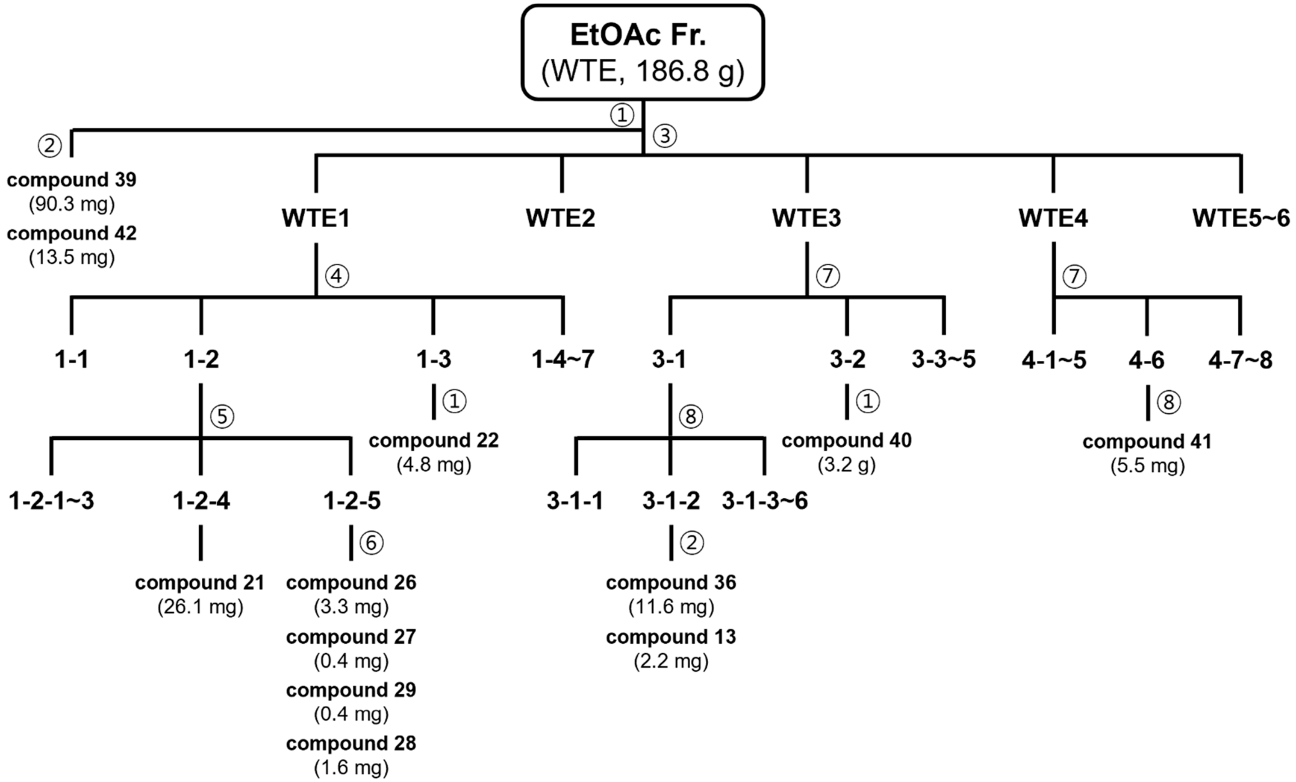

① Recrystallization (MeOH)
② semi-prep HPLC (0.1% formic acid in CH_3CN: 0.1% formic acid in H_2O = 35:65)
③ Silica gel CC (CH_2Cl_2:MeOH = 7:1 → 0:1)
④ Silica gel CC (hexane:EtOAc = 1:2 → 0:1)
⑤ prep-TLC (hexane:EtOAc = 1:1)
⑥ semi-prep HPLC (0.1% formic acid in CH_3CN: 0.1% formic acid in H_2O = 45:55)
⑦ Silica gel CC (EtOAc:MeOH = 10:1 → 0:1)
⑧ Sephadex LH-20 (100% MeOH)

1.4. Isolation and Purification of Compounds from n-BuOH Fraction

The *n*-BuOH fraction (182.2 g) was subjected to silica gel CC using EtOAc-MeOH (10:1–0:1), resulting in seven fractions (WTB1 ~ WTB7). Fraction WTB3 (3.6 g) was further fractionated by silica gel CC using $CHCl_3$-MeOH (7:1–0:1) and yielded eight subfractions (WTB3-1 ~ WTB3-8). Subfraction WTB3-4 (210.1 mg) was subjected to Sephadex LH-20 CC with MeOH and purified by semi-prep HPLC (Shimadzu system, UV wavelength at 280 and 350 nm, 2 mL/min, Watchers 120 ODS-BP column, 10 µm, 10.0 mm × 250 mm) using MeOH (A)-H_2O (B) (0-5 min, 5% A; 5-50 min, 5-95% A; 50-60 min, 95% A), yielding compounds **19** (t_R = 31.0 min, 35.0 mg) and **1** (t_R = 53.4 min, 32.9 mg). Subfraction WTB3-5 (589.0 mg) was recrystallized from MeOH to isolate compound **8** (134.8 mg). Fraction WTB4 (8.5 g) was further separated by silica gel CC using $CHCl_3$-MeOH (30:1–0:1), resulting in ten subfractions (WTB4-1 ~ WTB4-10). Subfractions WTB4-2 (50.6 mg) and WTB4-6 (61.7 mg) were purified by semi-prep HPLC (Shimadzu system, UV wavelength at 280 and 350 nm, 2 mL/min, Watchers 120 ODS-BP column, 10 µm, 10.0 mm × 250 mm) using MeOH (A)-H_2O (B) (0-10 min, 25% A; 10-45 min, 25-95% A; 45-55 min, 95% A) and yielded compounds **20** (t_R = 28.8 min, 1.5 mg), **4** (t_R = 37.6 min, 6.0 mg), **7** (t_R = 40.9 min, 3.2 mg), and **37** (t_R = 47.5 min, 4.8 mg). Fraction WTB5 (89.1 g) was separated by silica gel CC using EtOAc-MeOH (5:1–0:1) and yielded seven subfractions (WTB5-1 ~ WTB5-7). Subfraction WTB5-1 (49.4 mg) was purified by semi-prep HPLC (Shimadzu system, UV wavelength at 254 and 330 nm, 2 mL/min, Watchers 120 ODS-BP column, 10 µm, 10.0 mm × 250 mm) using 0.1% formic acid in CH_3CN (A)-0.1% formic acid in H_2O (B) (0-10 min, 20% A; 10-55 min, 20-95% A; 55-65 min, 95% A) to yield compound **5** (t_R = 46.4 min, 1.2 mg). Subfractions WTB5-4 (948.4 mg) and WTB5-5 (5.5 g) were purified by semi-prep HPLC (Gilson system, 2 mL/min, INNO C18 column, 10 µm, 10.0 mm × 250 mm) using MeOH (A)-H_2O (B) (0-10 min, 5% A; 10-50 min, 5-100% A) and yielded compounds **38** (9.4 mg) and **15** (2.7 mg). Subfraction WTB5-6 (46.3 g) was recrystallized from MeOH to isolate compound **24** (29.3 mg). Fraction WTB6 (11.4 g) was separated by semi-prep HPLC (Gilson system, 2 mL/min, INNO C18 column, 10 µm, 10.0 mm × 250 mm) using MeOH (A)-H_2O (B) (0-10 min, 10% A; 10-60 min, 10-100% A) to give six subfractions (WTB6-1 ~ WTB6-6). Subfraction WTB6-1 (596.7 mg) was purified by semi-prep HPLC (Shimadzu system, UV wavelength at 254 and 330 nm, 2 mL/min, Watchers 120 ODS-BP column, 10 µm, 10.0 mm × 250 mm) using 0.1% formic acid in CH_3CN-0.1% formic acid in H_2O (7:93), to yield compounds **11** (t_R = 47.2 min, 1.4 mg), **9** (t_R = 86.7 min, 4.0 mg), and **10** (t_R = 100.2 min, 0.4 mg). Subfraction WTB6-3 (637.5 mg) was subjected to

semi-prep HPLC (Shimadzu system, UV wavelength at 254 and 330 nm, 2 mL/min, Watchers 120 ODS-BP column, 10 µm, 10.0 mm × 250 mm) using 0.1% formic acid in CH_3CN-0.1% formic acid in H_2O (22:78), to yield compounds **30** (t_R = 17.4 min, 0.6 mg), **32** (t_R = 18.9 min, 82.8 mg), **25** (t_R = 25.3 min, 18.1 mg), **33** (t_R = 30.1 min, 64.5 mg), and **31** (t_R = 37.5 min, 30.5 mg). Subfraction WTB6-4 (189.1 mg) was purified by semi-prep HPLC (Shimadzu system, UV wavelength at 280 and 350 nm, 2 mL/min, Watchers 120 ODS-BP column, 10 µm, 10.0 mm × 250 mm) using 0.1% formic acid in CH_3CN (A)-0.1% formic acid in H_2O (B) (0-10 min, 23% A; 10-15 min, 23-28% A; 15-50 min, 28-35% A; 50-60 min, 35-90% A; 60-65 min, 90% A) to yield compound **2** (t_R = 13.7 min, 5.0 mg).

Figure S3. Isolation of compounds from the *n*-BuOH fraction of the *W. trichotoma* extract.

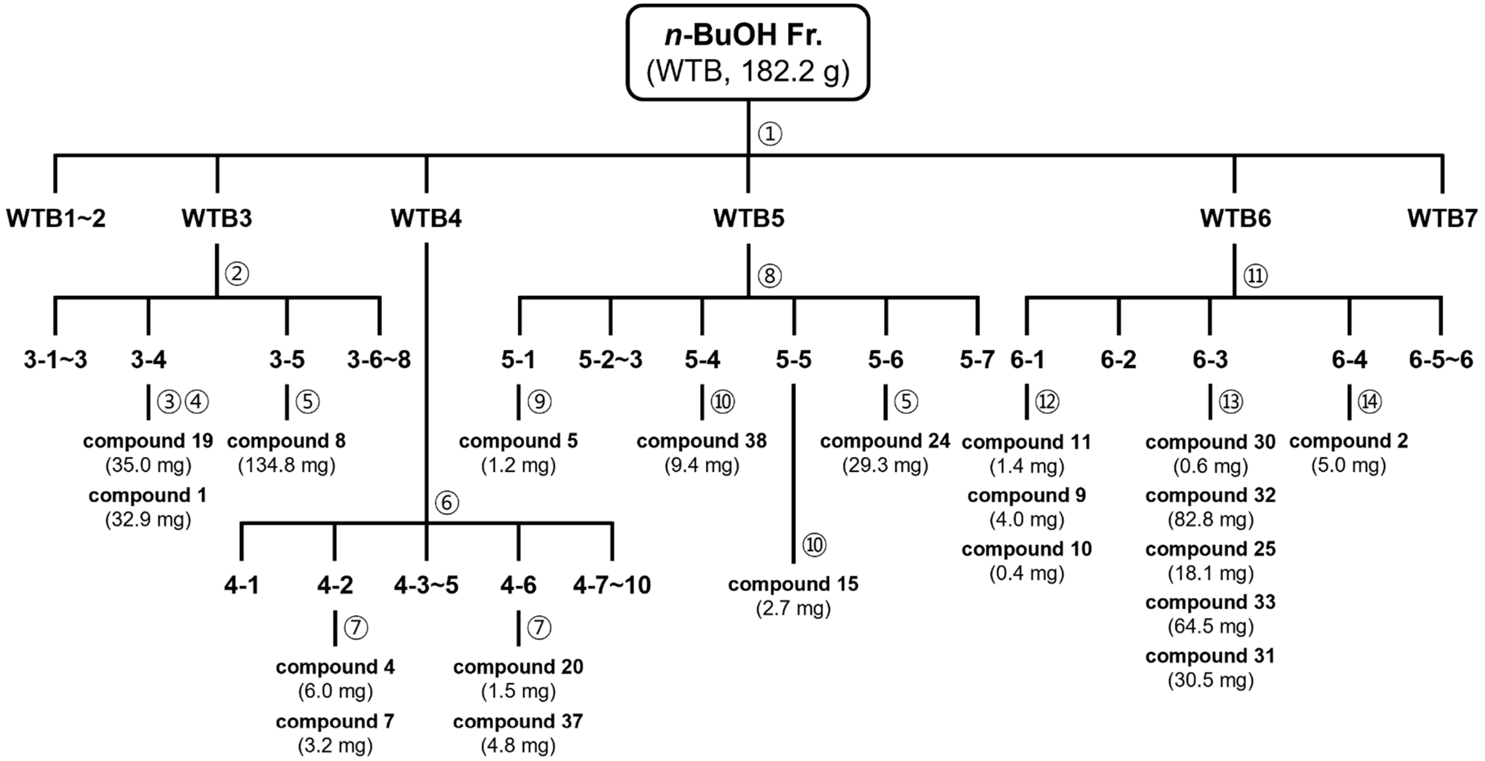

① Silica gel CC (EtOAc:MeOH = 10:1 → 0:1)
② Silica gel CC ($CHCl_3$:MeOH = 7:1 → 0:1)
③ Sephadex LH-20 (100% MeOH)
④ semi-prep HPLC (MeOH:H_2O = 5:95 → 95:5)
⑤ Recrystallization (MeOH)
⑥ Silica gel CC ($CHCl_3$:MeOH = 30:1 → 0:1)
⑦ semi-prep HPLC (MeOH:H_2O = 25:75 → 95:5)
⑧ Silica gel CC (EtOAc:MeOH = 5:1 → 0:1)
⑨ semi-prep HPLC (0.1% formic acid in CH_3CN: 0.1% formic acid in H_2O = 20:80 → 95:5)
⑩ semi-prep HPLC (MeOH:H_2O = 5:95 → 1:0)
⑪ semi-prep HPLC (MeOH:H_2O = 10:90 → 1:0)
⑫ semi-prep HPLC (0.1% formic acid in CH_3CN: 0.1% formic acid in H_2O = 7:93)
⑬ semi-prep HPLC (0.1% formic acid in CH_3CN: 0.1% formic acid in H_2O = 22:78)
⑭ semi-prep HPLC (0.1% formic acid in CH_3CN: 0.1% formic acid in H_2O = 23:77 → 90:10)

Figure S4. The HR-ESI-MS data of compound **1**.

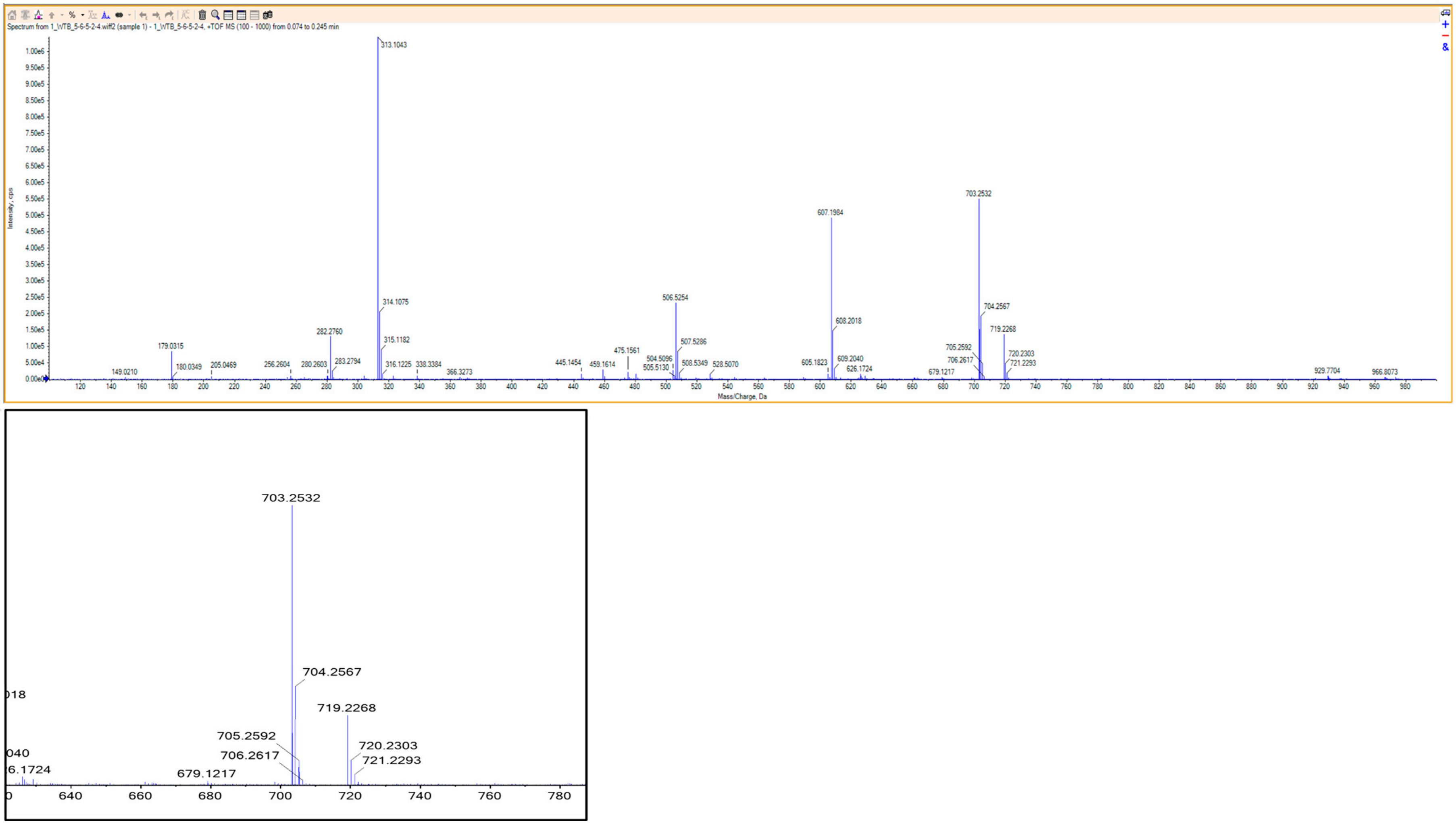

Figure S5. The ^{1}H NMR spectrum of compound **1** (DMSO-d_6, 400 MHz).

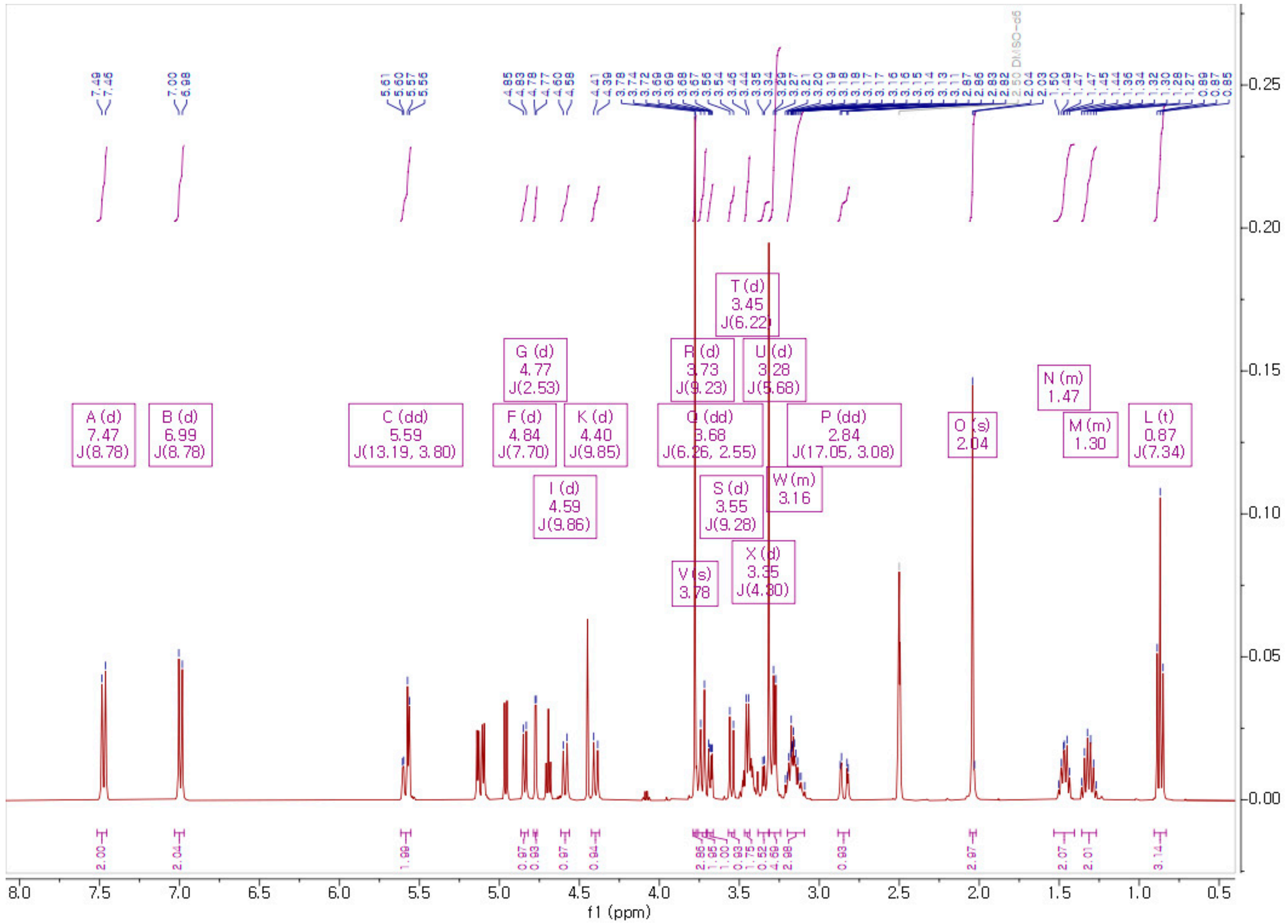

Figure S6. The ^{13}C NMR spectrum of compound **1** (DMSO-d_6, 100 MHz).

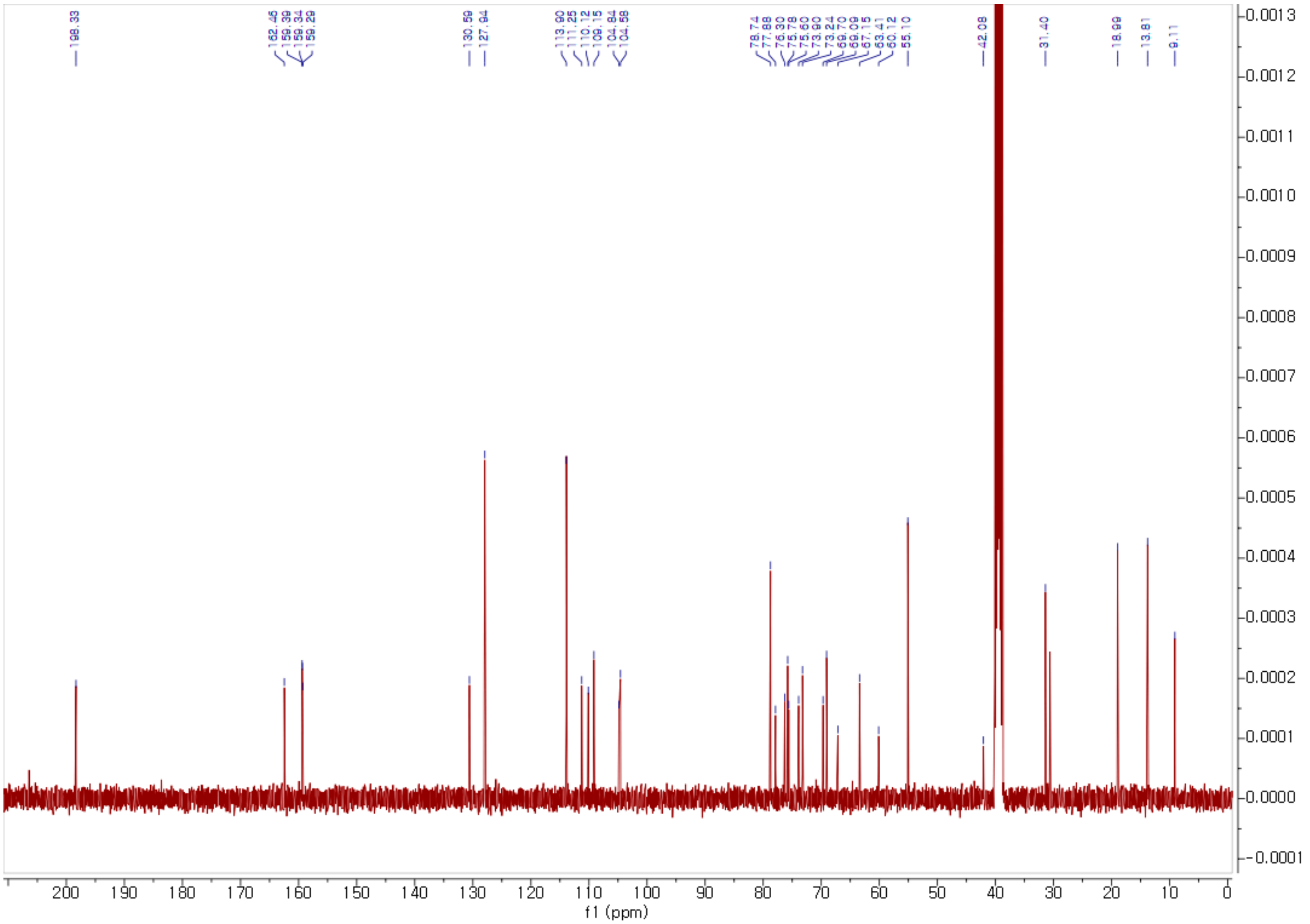

Figure S7. The ^{1}H-^{1}H COSY spectrum of compound **1** (DMSO-d_6).

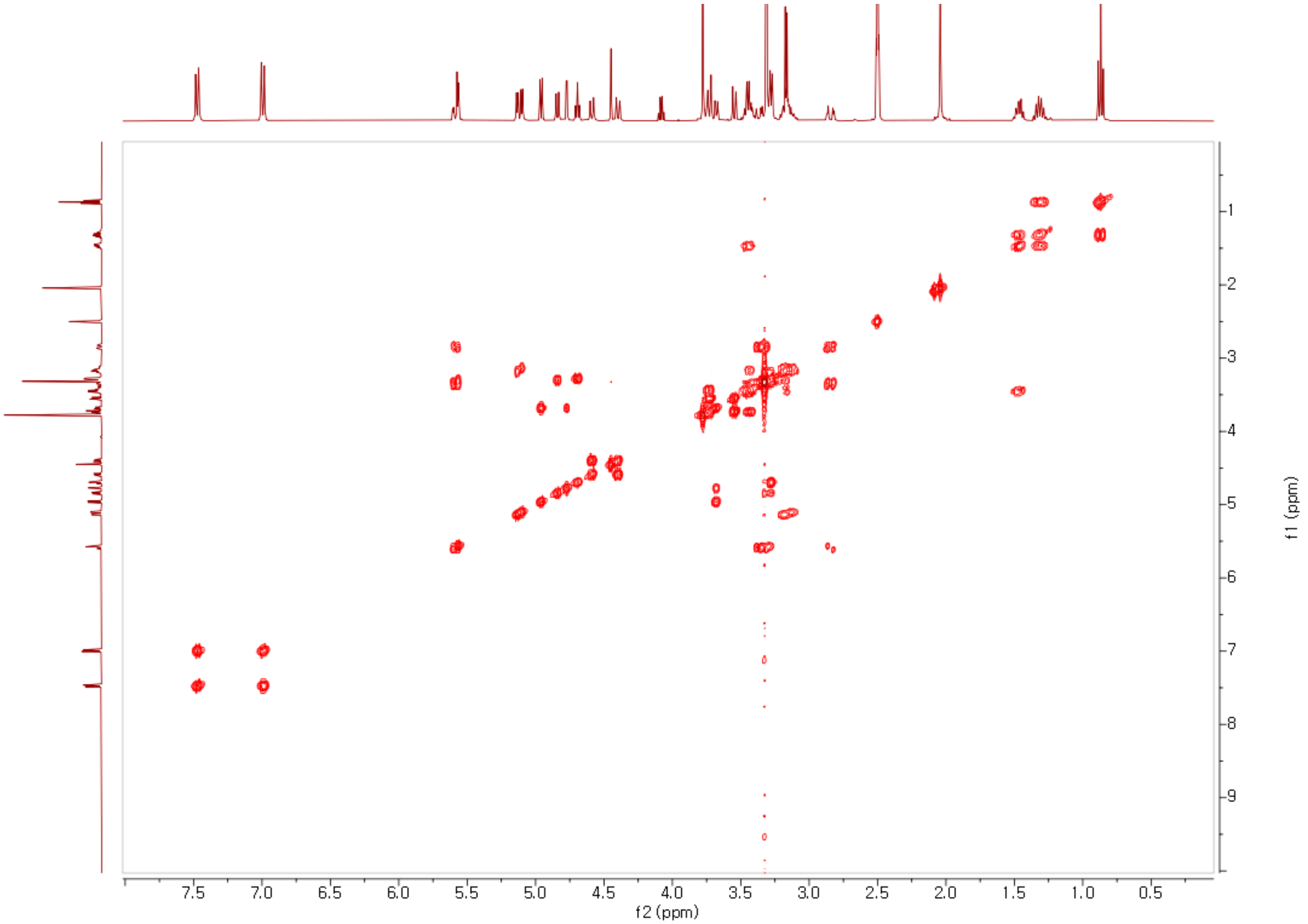

Figure S8. The HSQC spectrum of compound **1** (DMSO-d_6).

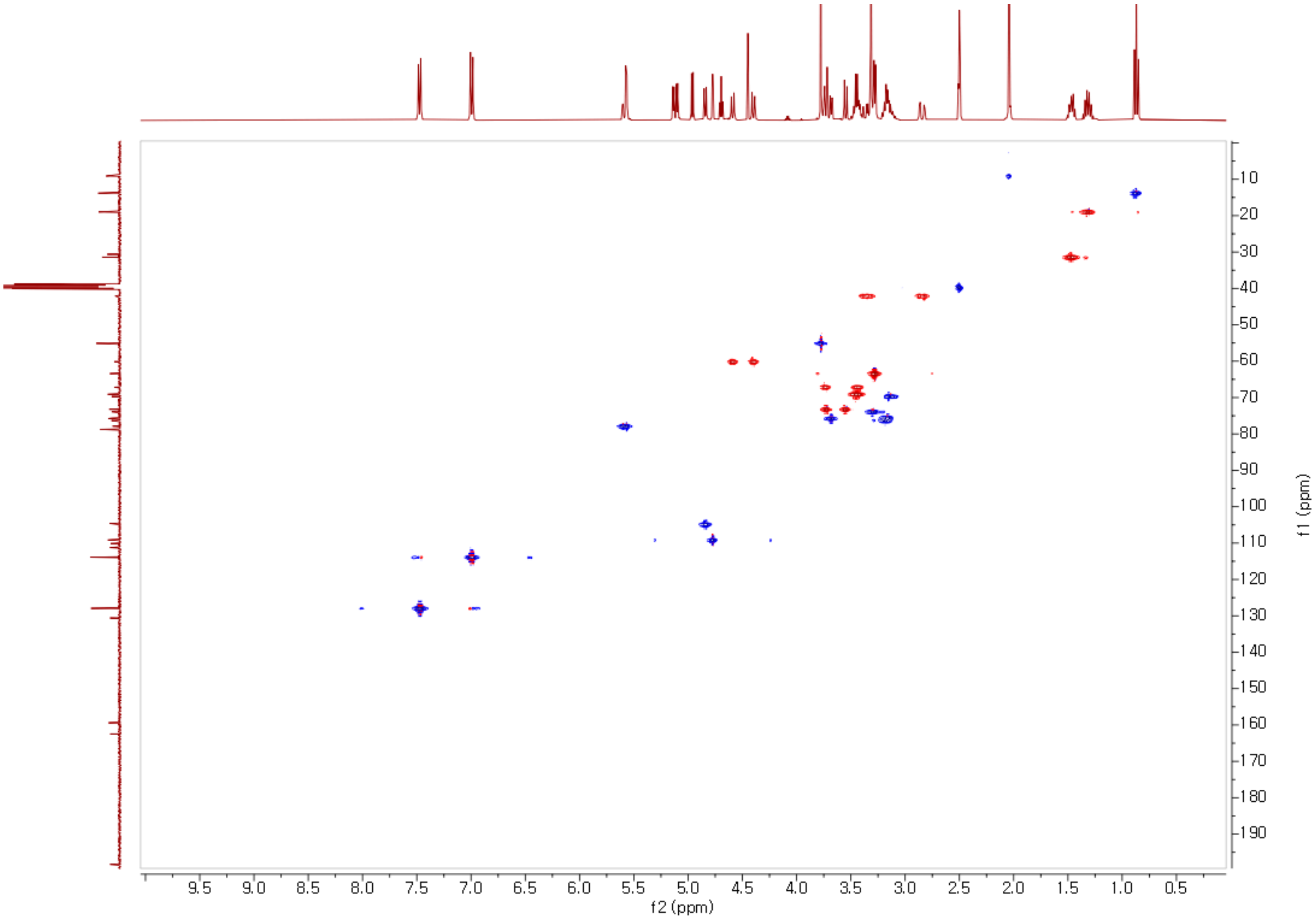

Figure S9. The HMBC spectrum of compound **1** (DMSO-d_6).

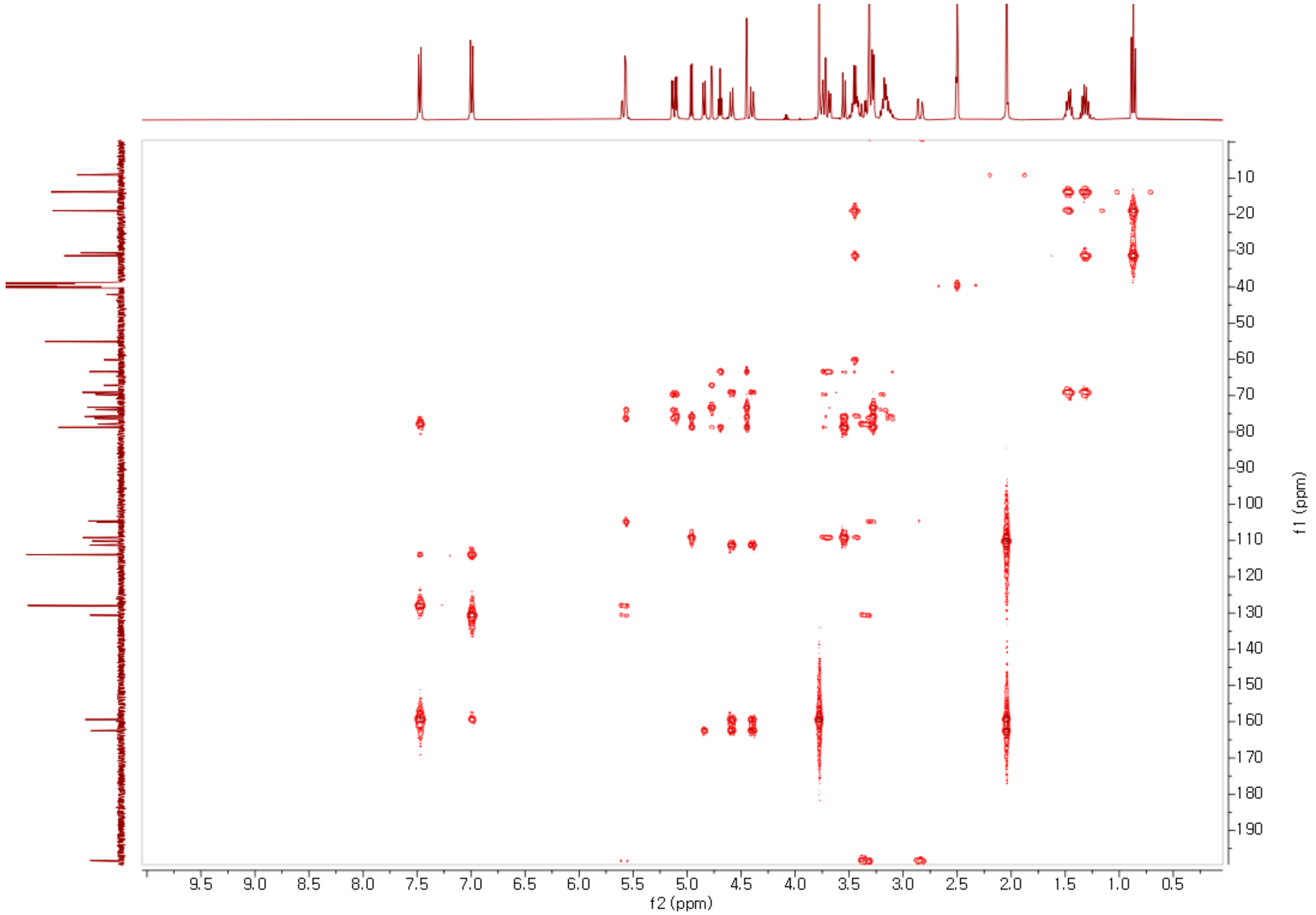

Figure S10. The ECD spectrum of compound **1**.

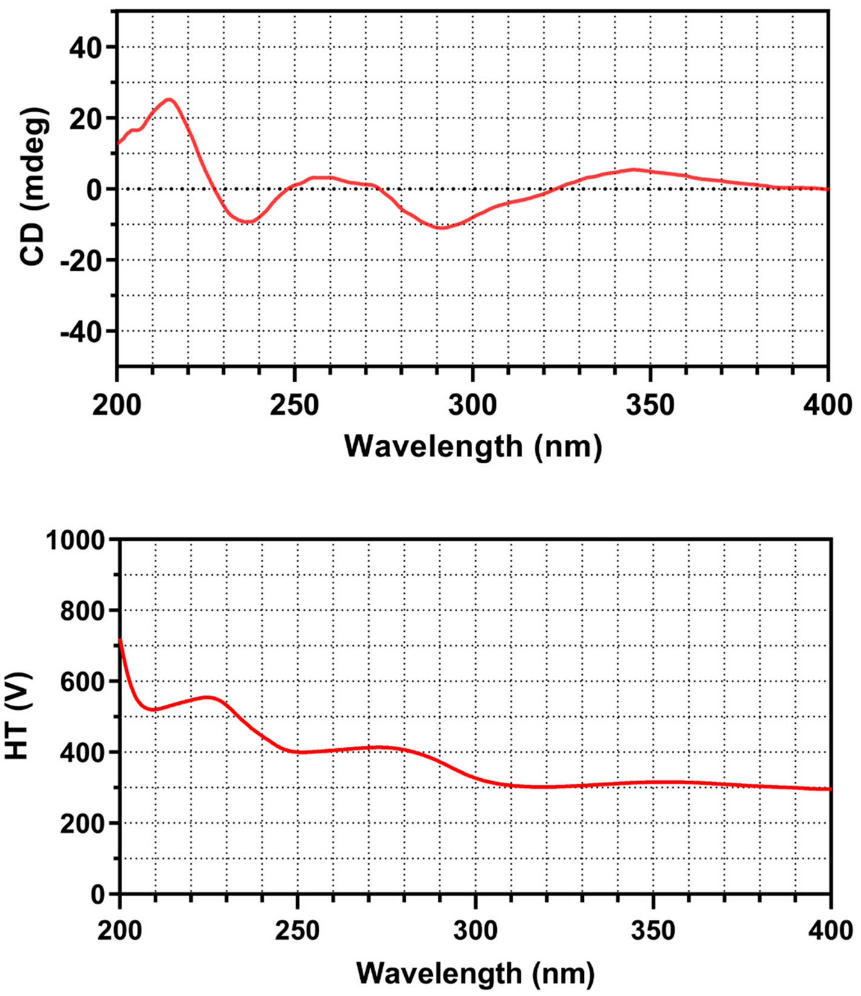

Figure S11. The HR-ESI-MS data of compound **2**.

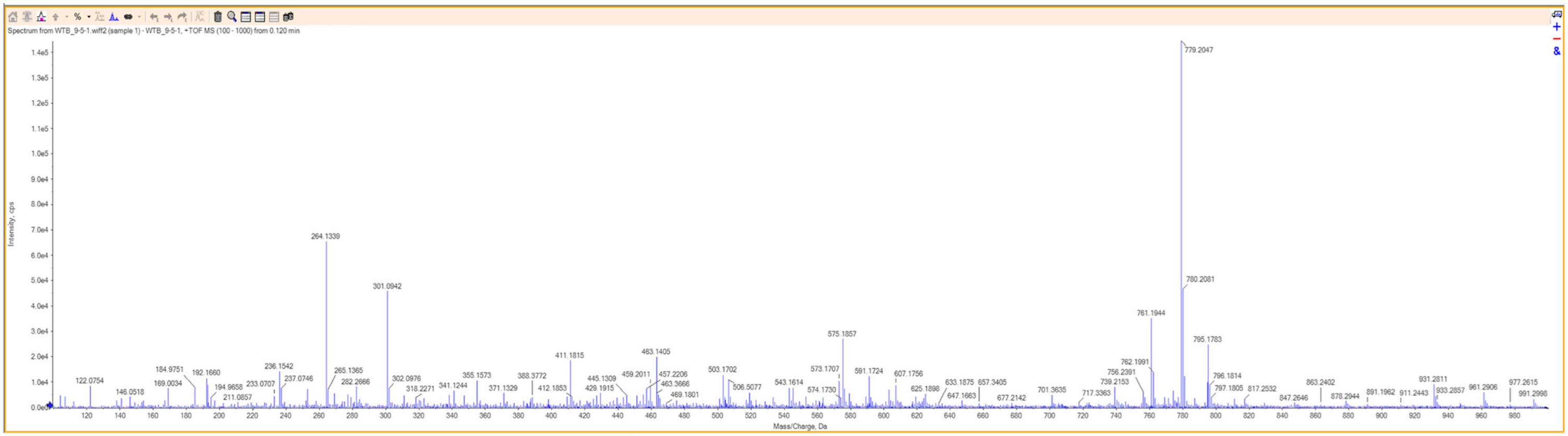

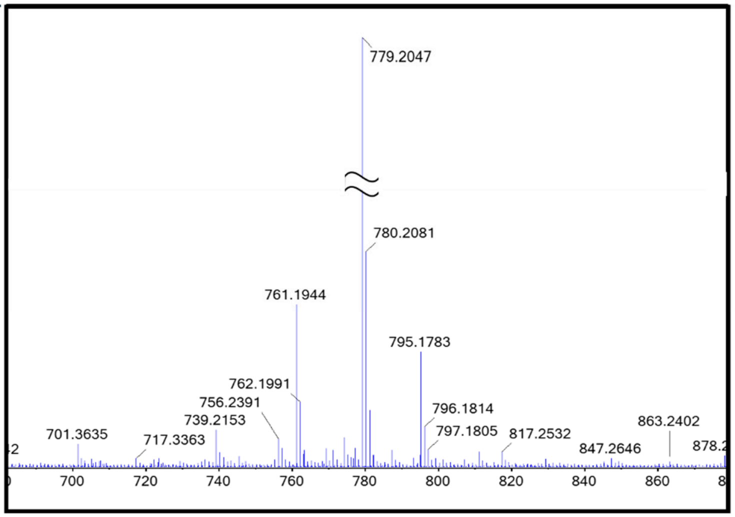

Figure S12. The ^{1}H NMR spectrum of compound **2** (DMSO-d_6, 400 MHz).

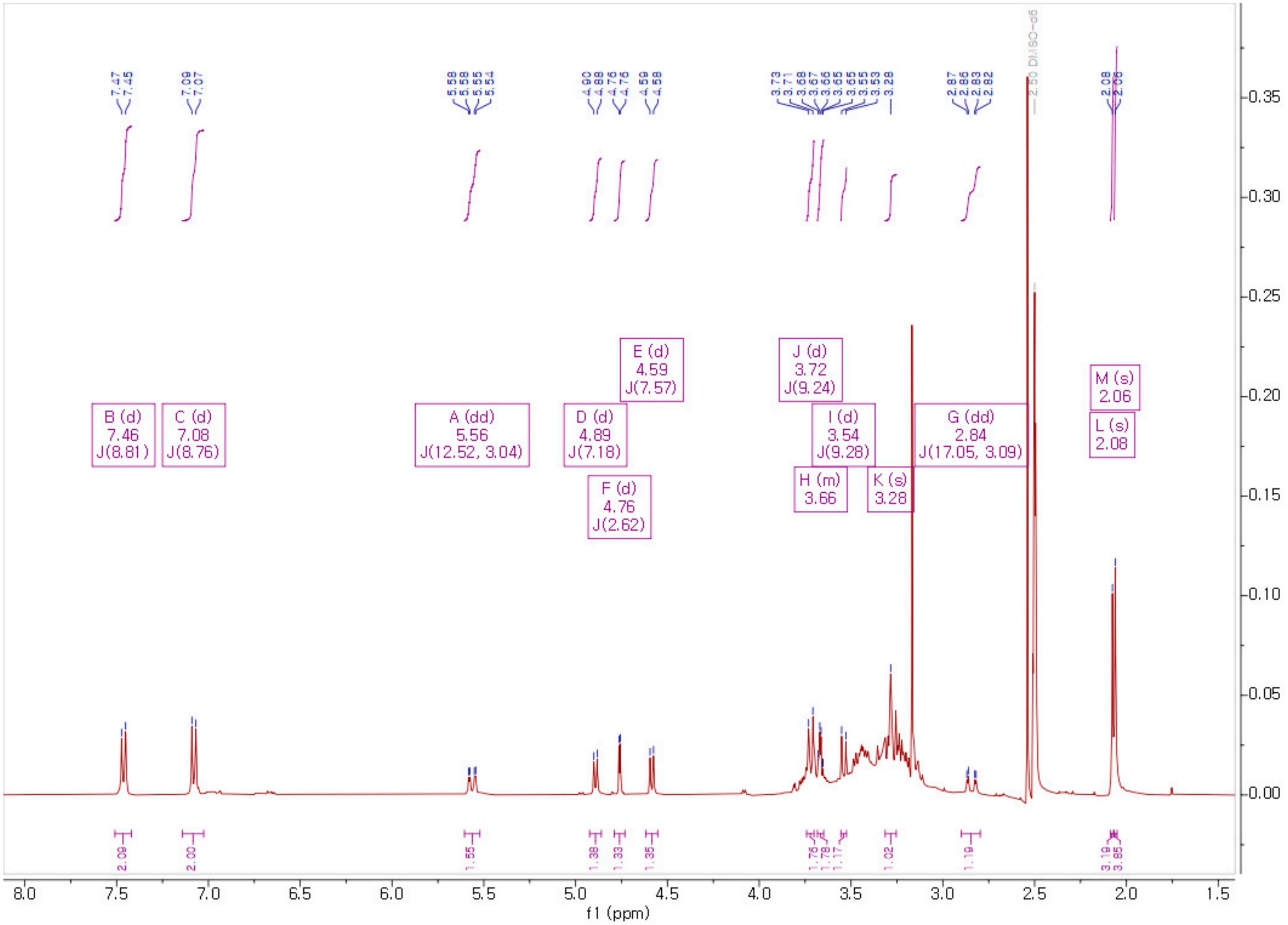

Figure S13. The ^{13}C NMR spectrum of compound **2** (DMSO-d_6, 100 MHz).

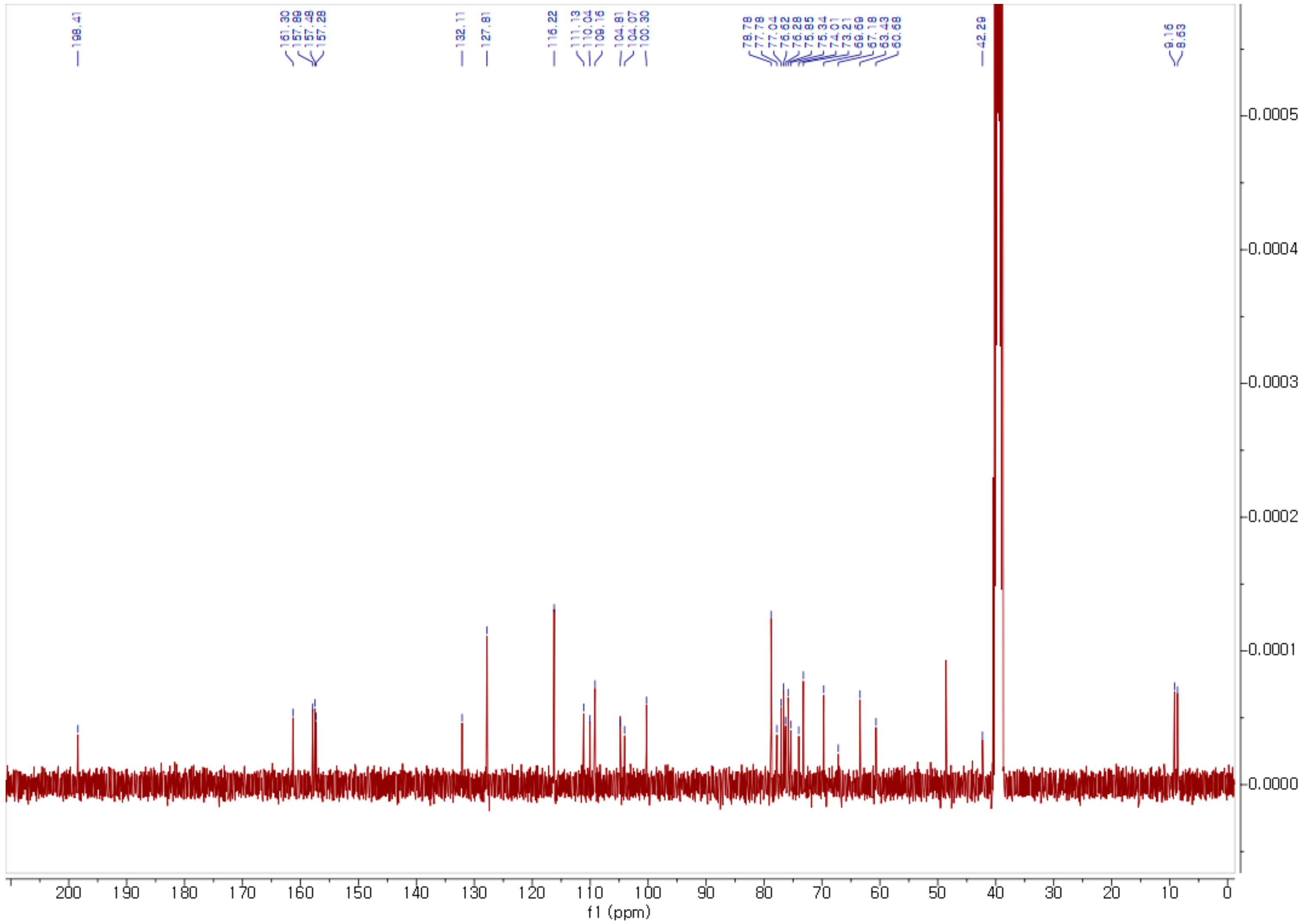

Figure S14. The ^{1}H-^{1}H COSY spectrum of compound **2** (DMSO-d_6).

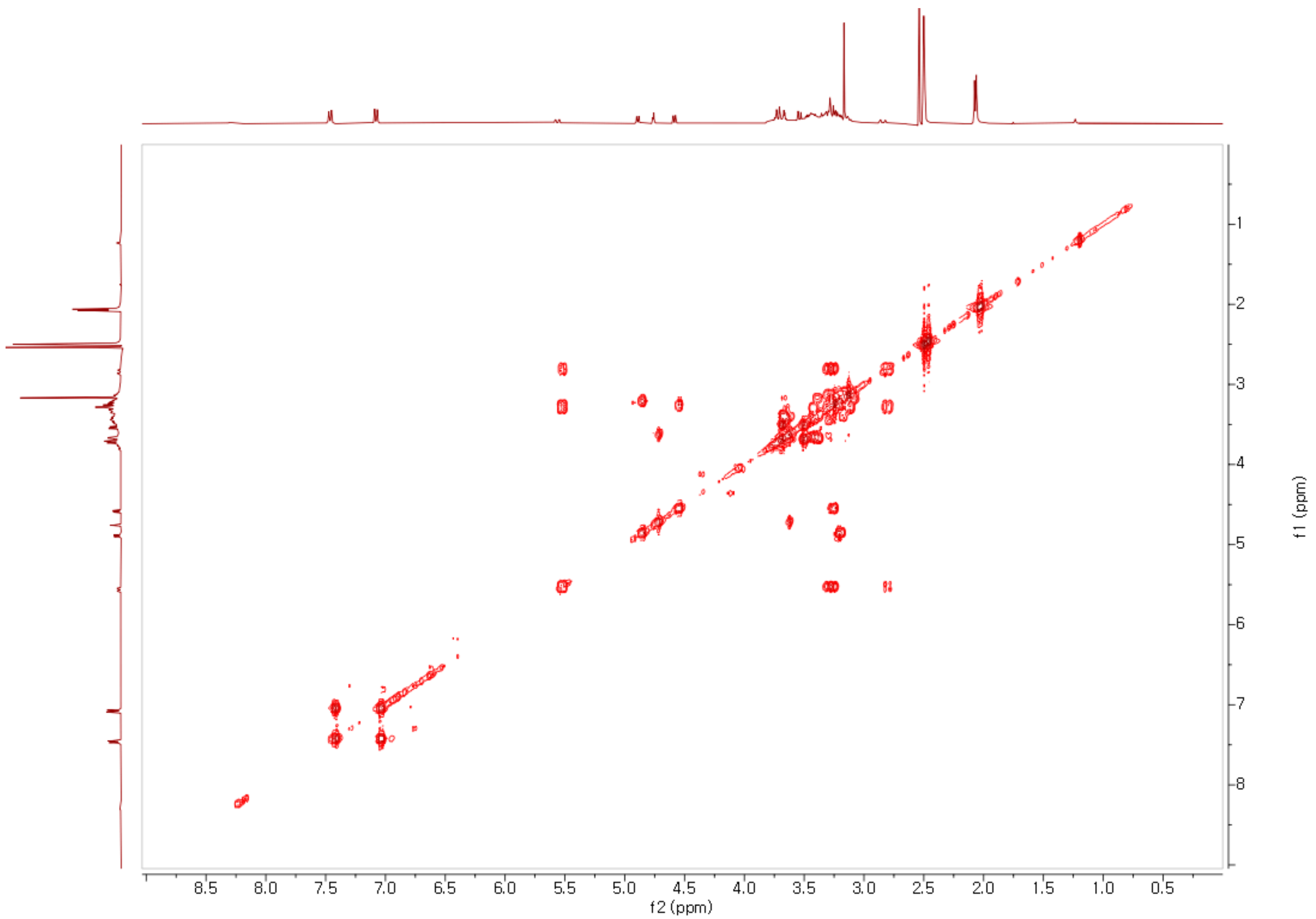

Figure S15. The HSQC spectrum of compound **2** (DMSO-d_6).

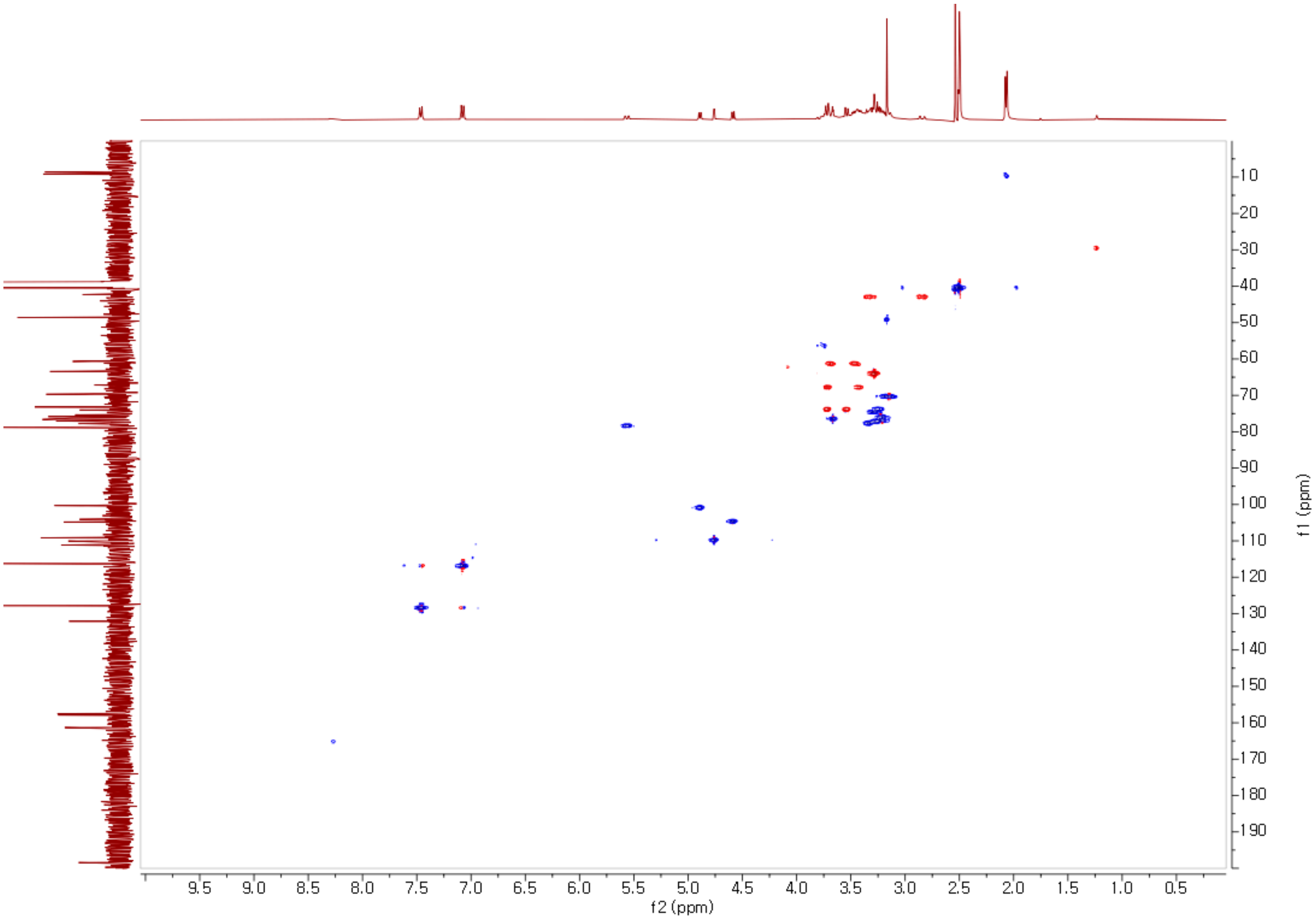

Figure S16. The HMBC spectrum of compound **2** (DMSO-d_6).

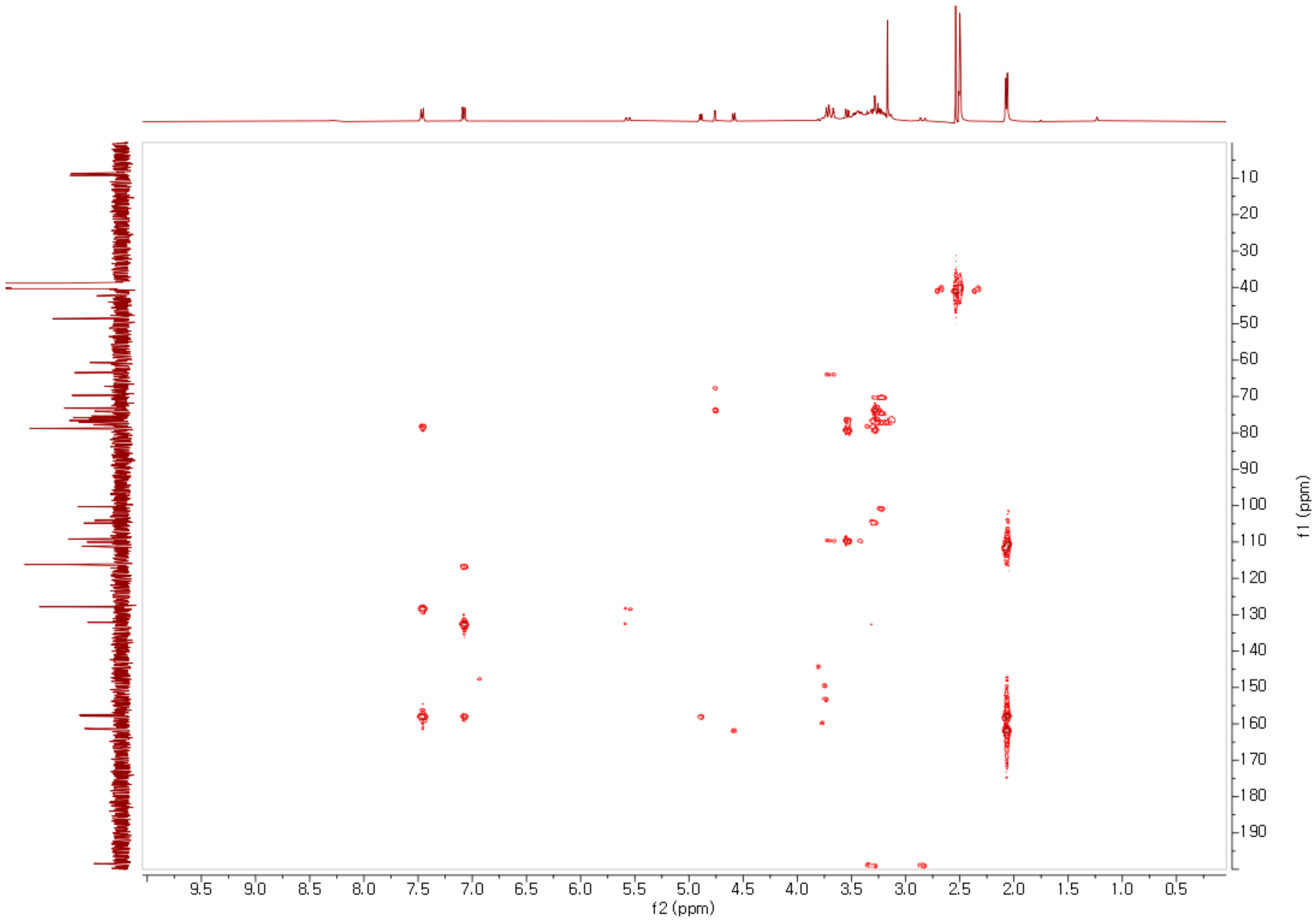

Figure S17. The ECD spectrum of compound **2**.

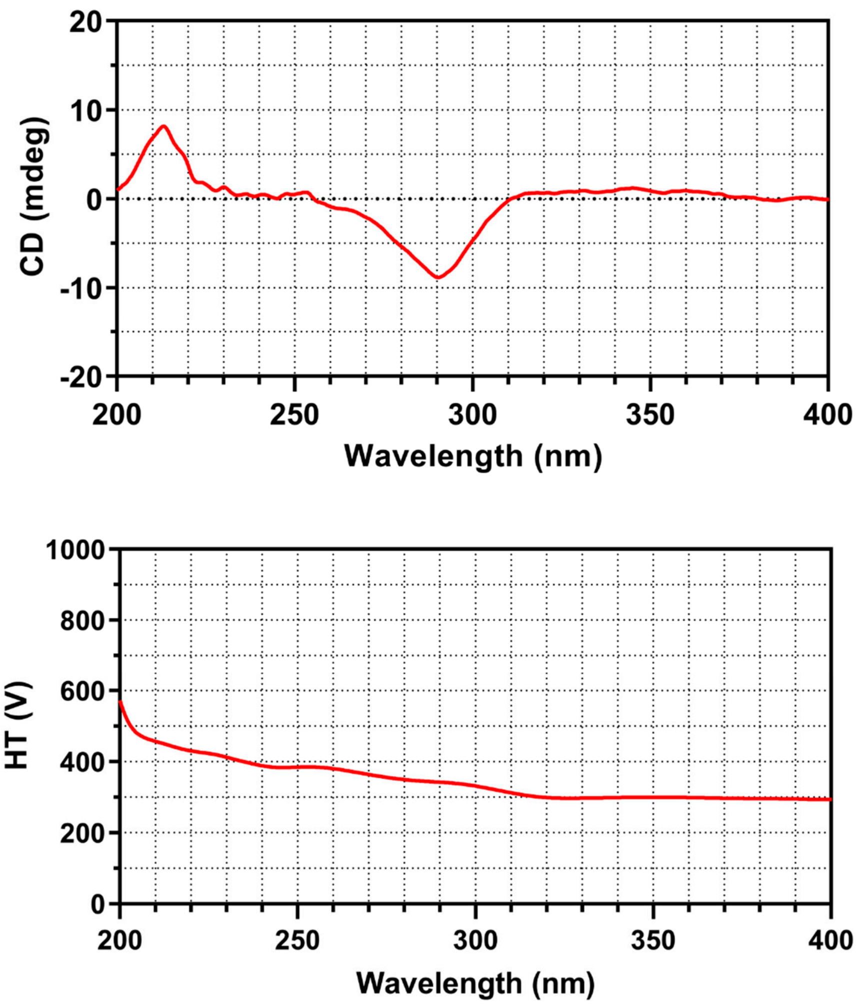

Figure S18. The ^{1}H NMR spectrum of compound **3** (DMSO-d_6, 400 MHz).

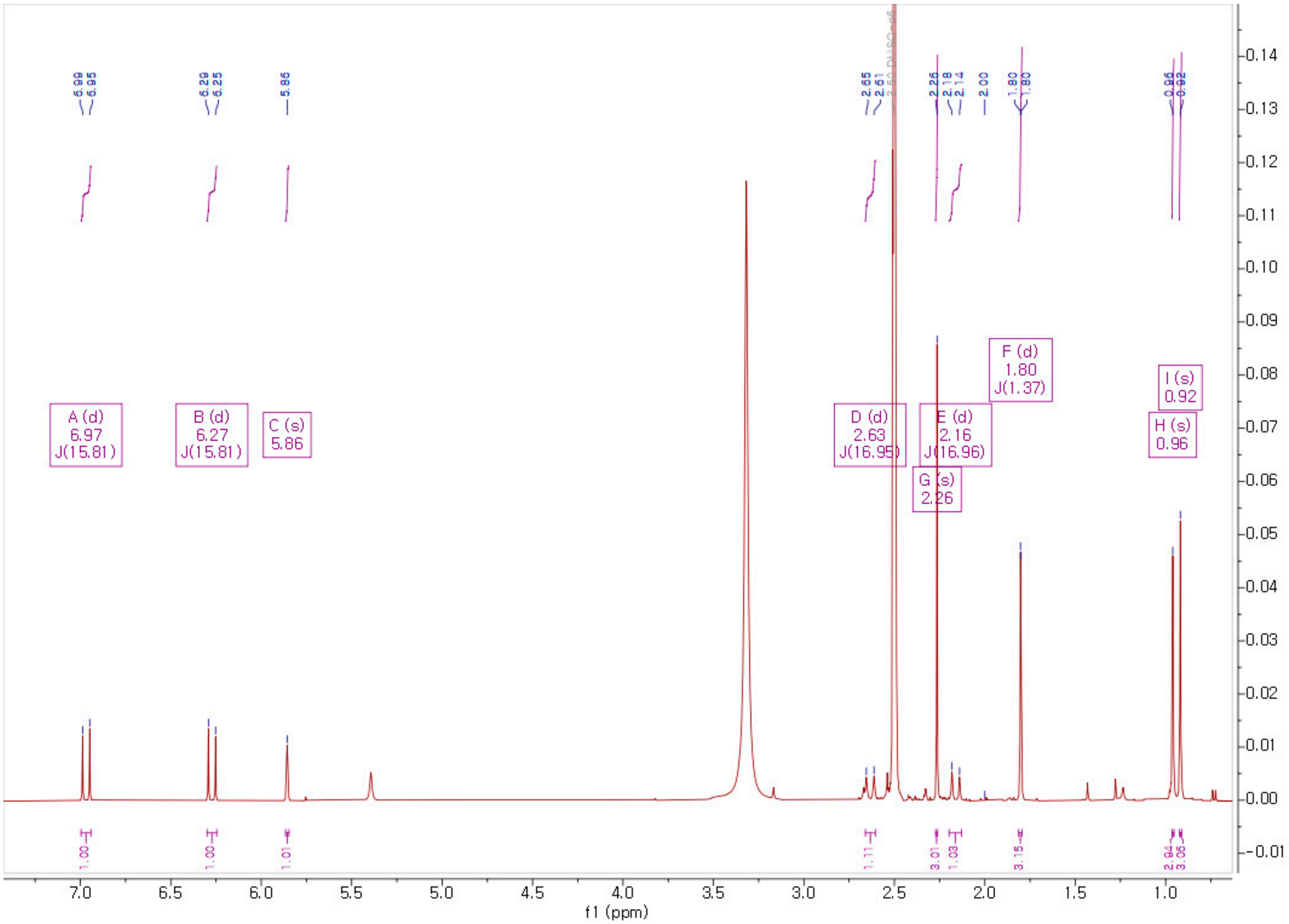

Figure S19. The ^{1}H NMR spectrum of compound **4** (DMSO-d_6, 400 MHz).

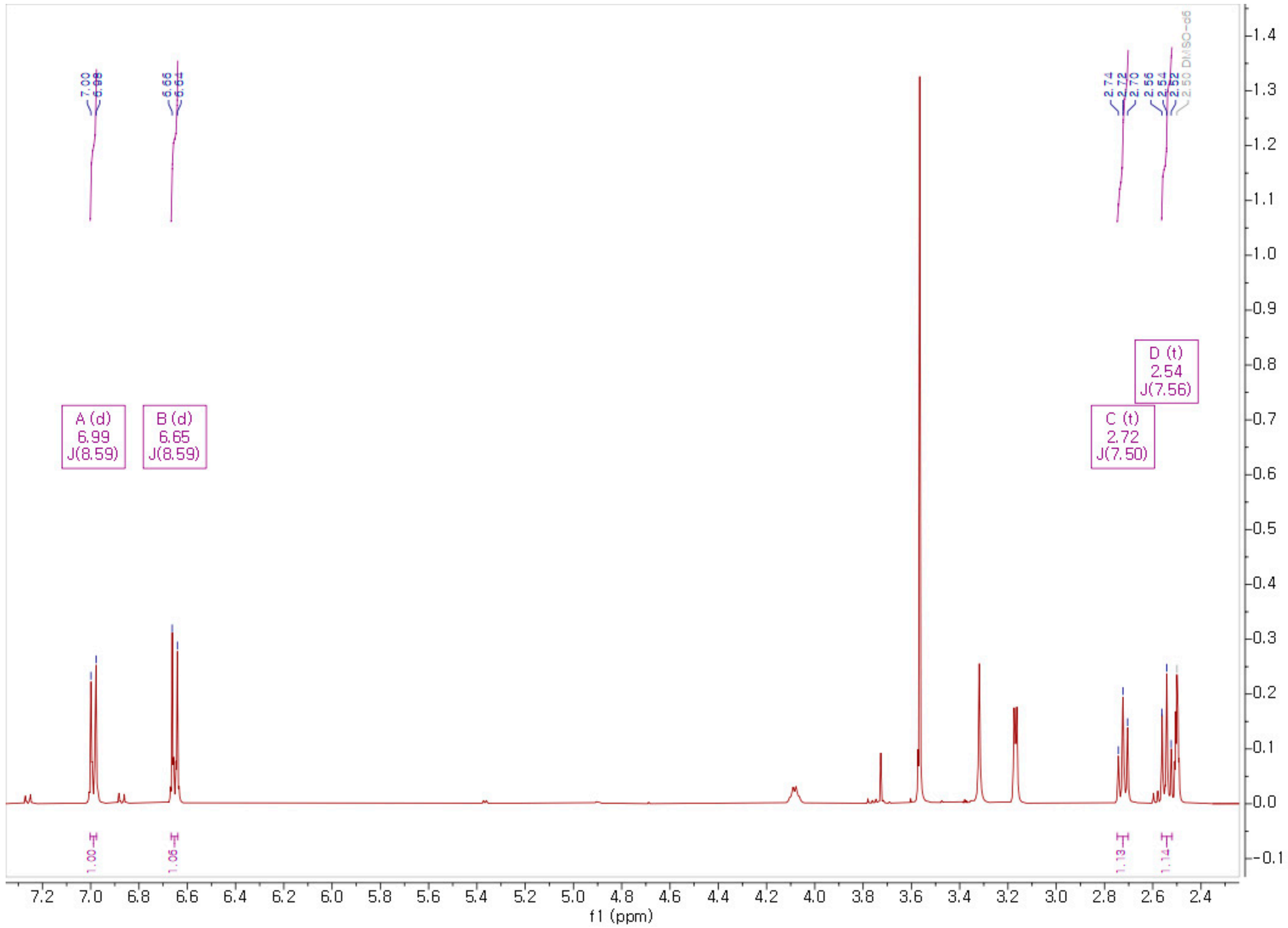

Figure S20. The ^{1}H NMR spectrum of compound **5** (DMSO-d_6, 400 MHz).

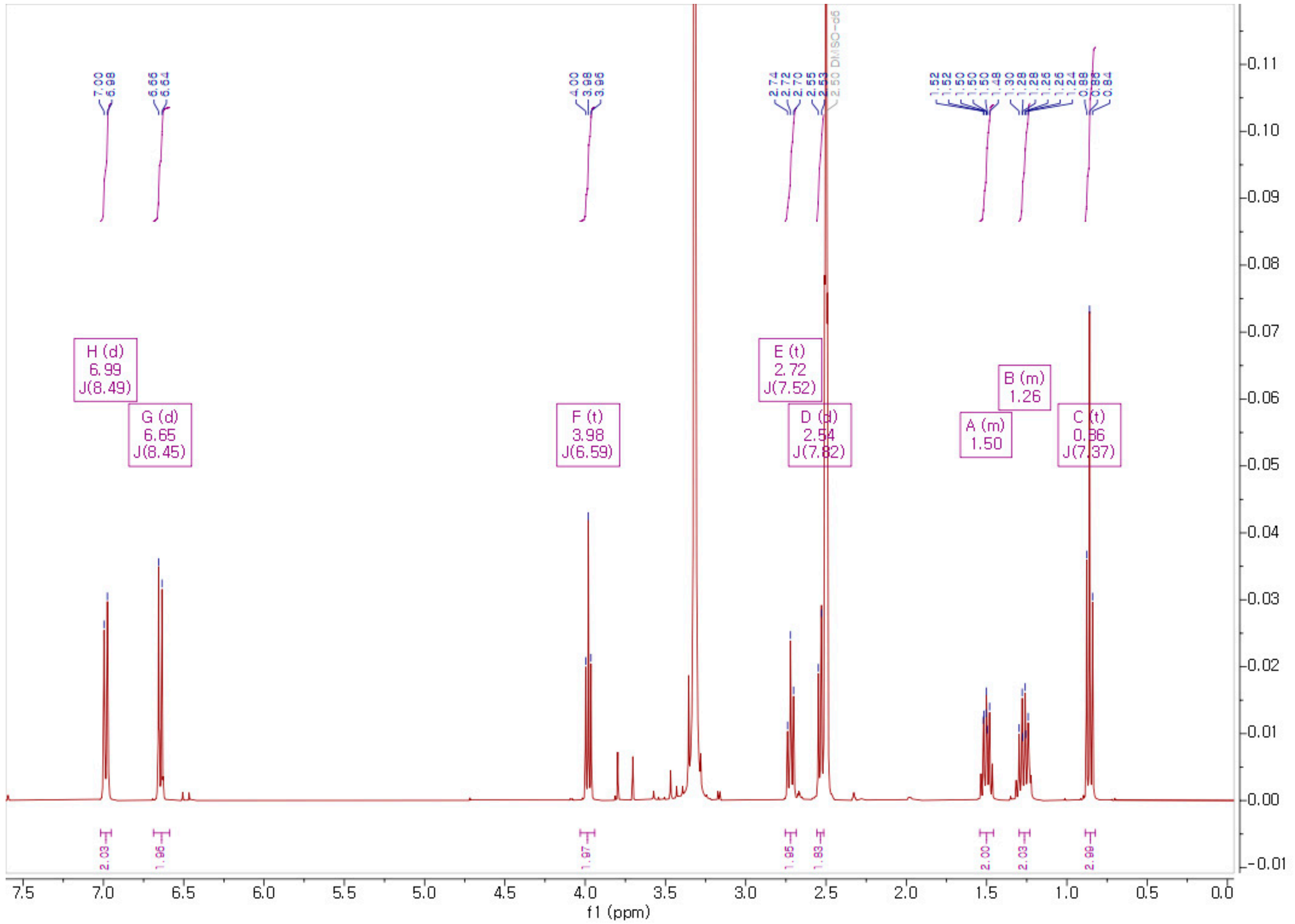

Figure S21. The ^{1}H NMR spectrum of compound **6** (DMSO-d_6, 400 MHz).

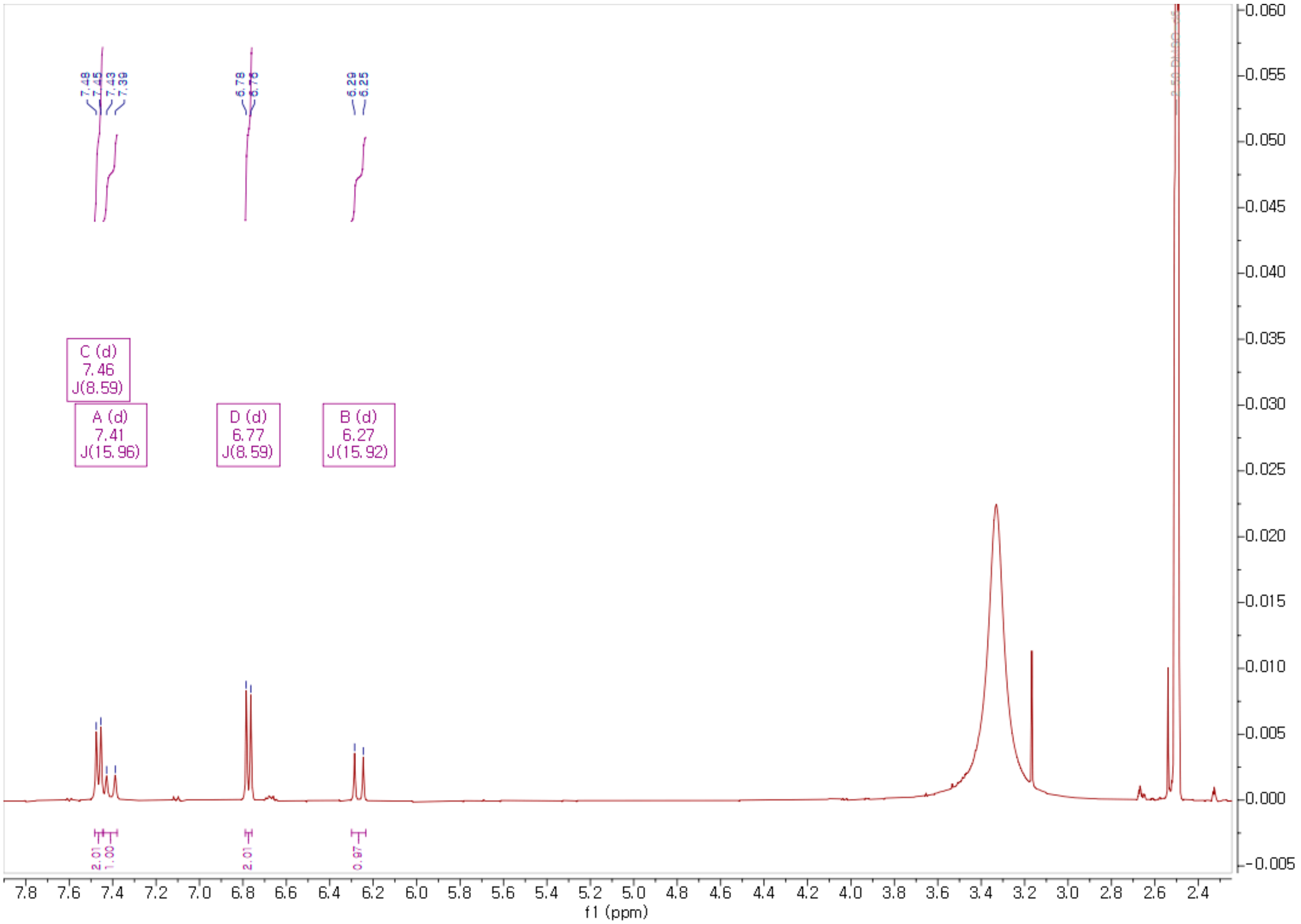

Figure S22. The ^{1}H NMR spectrum of compound **7** (DMSO-d_6, 400 MHz).

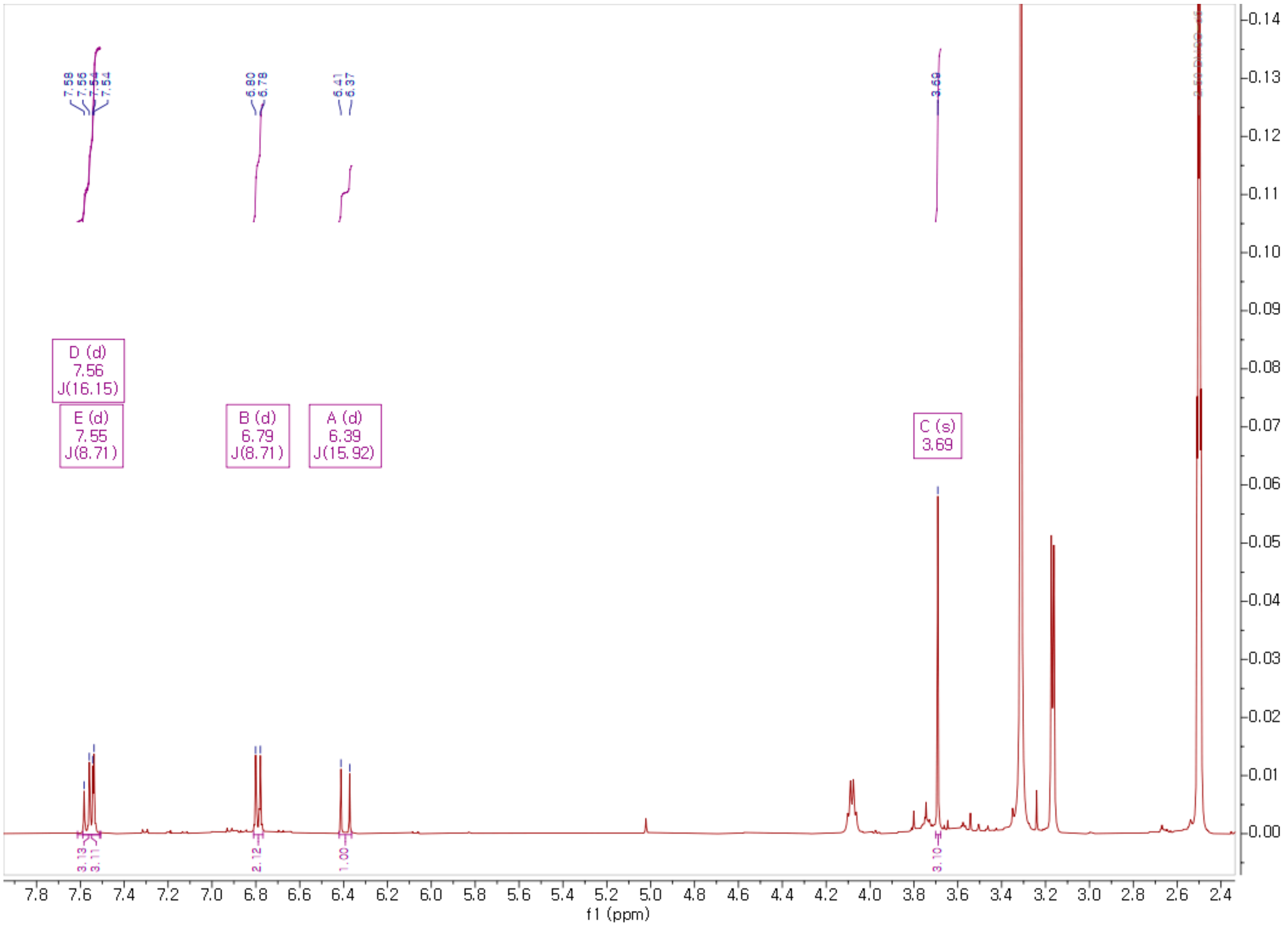

Figure S23. The ^{1}H NMR spectrum of compound **8** (DMSO-d_6, 400 MHz).

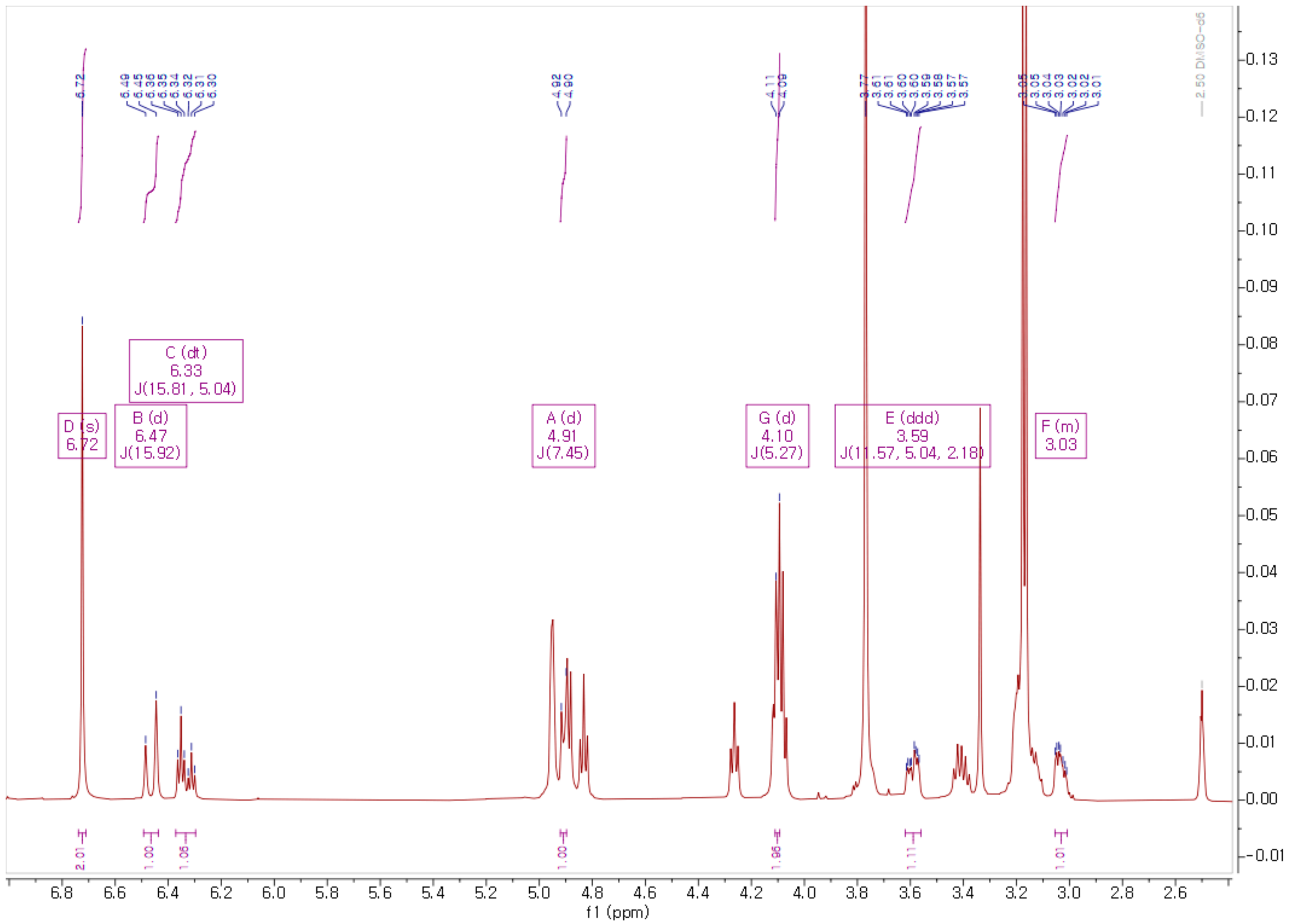

Figure S24. The ^{1}H NMR spectrum of compound **9** (DMSO-d_6, 400 MHz).

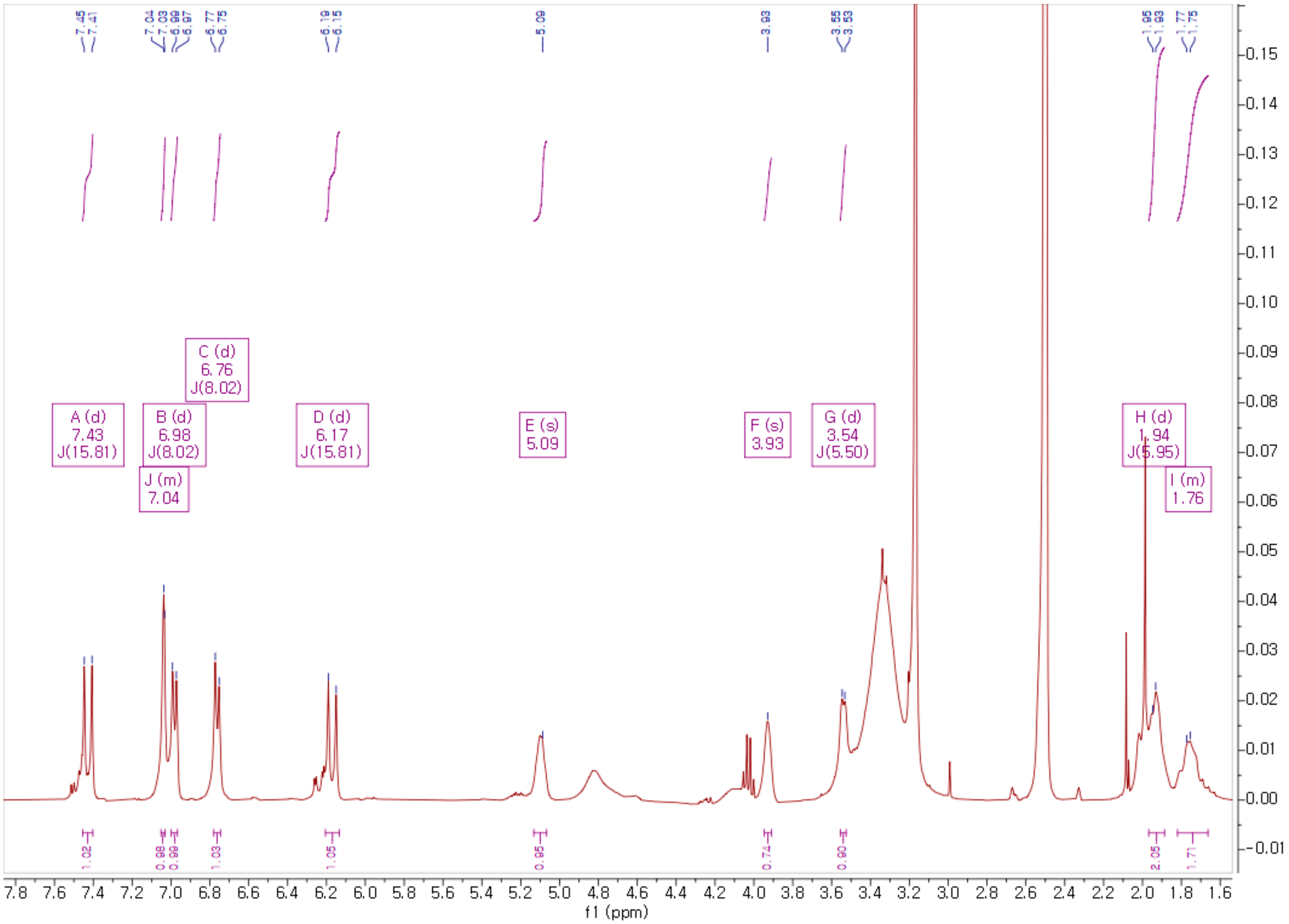

Figure S25. The ^{1}H NMR spectrum of compound **10** (DMSO-d_6, 400 MHz).

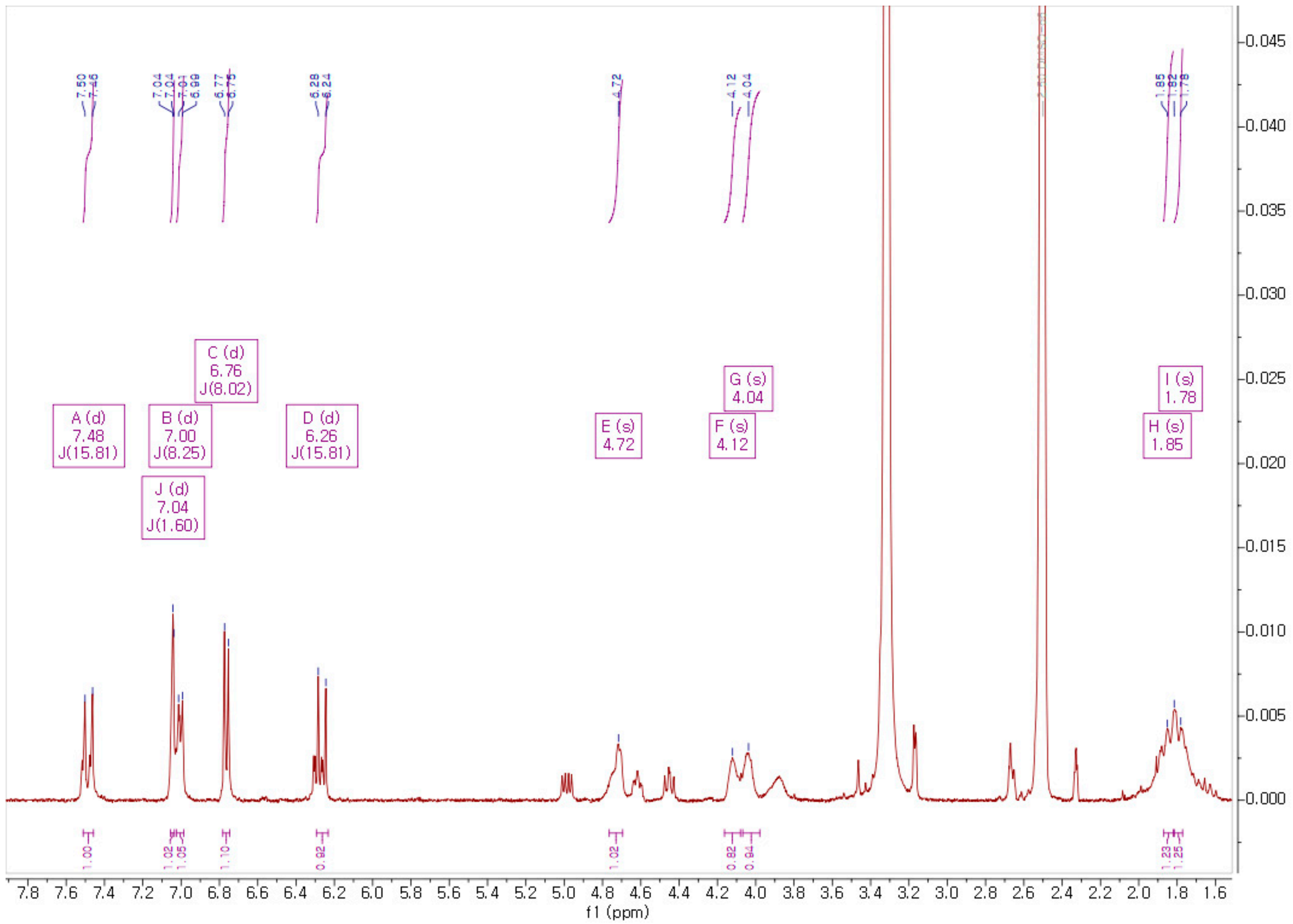

Figure S26. The ^{1}H NMR spectrum of compound **11** (DMSO-d_6, 400 MHz).

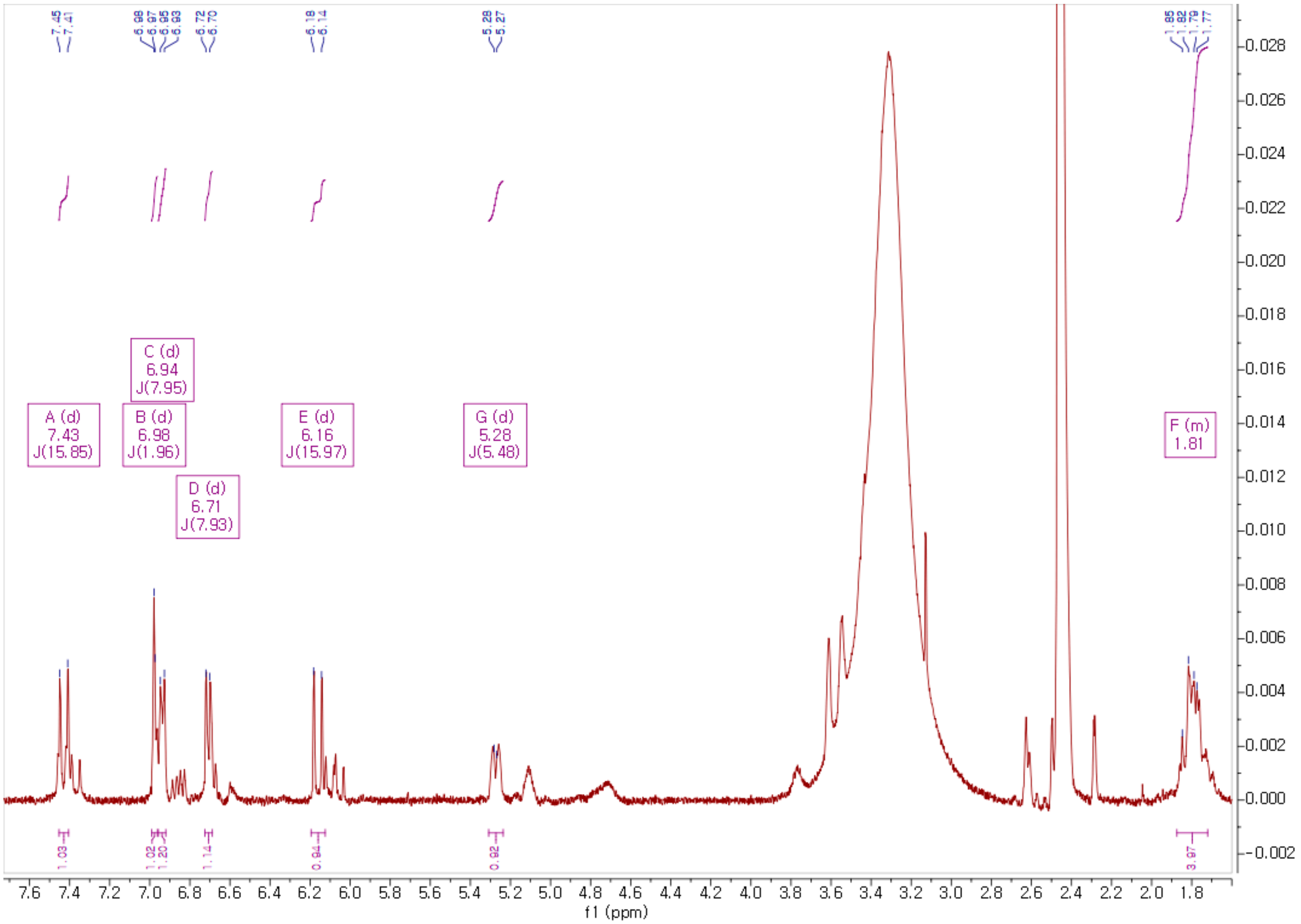

Figure S27. The ^{1}H NMR spectrum of compound **12** (DMSO-d_6, 400 MHz).

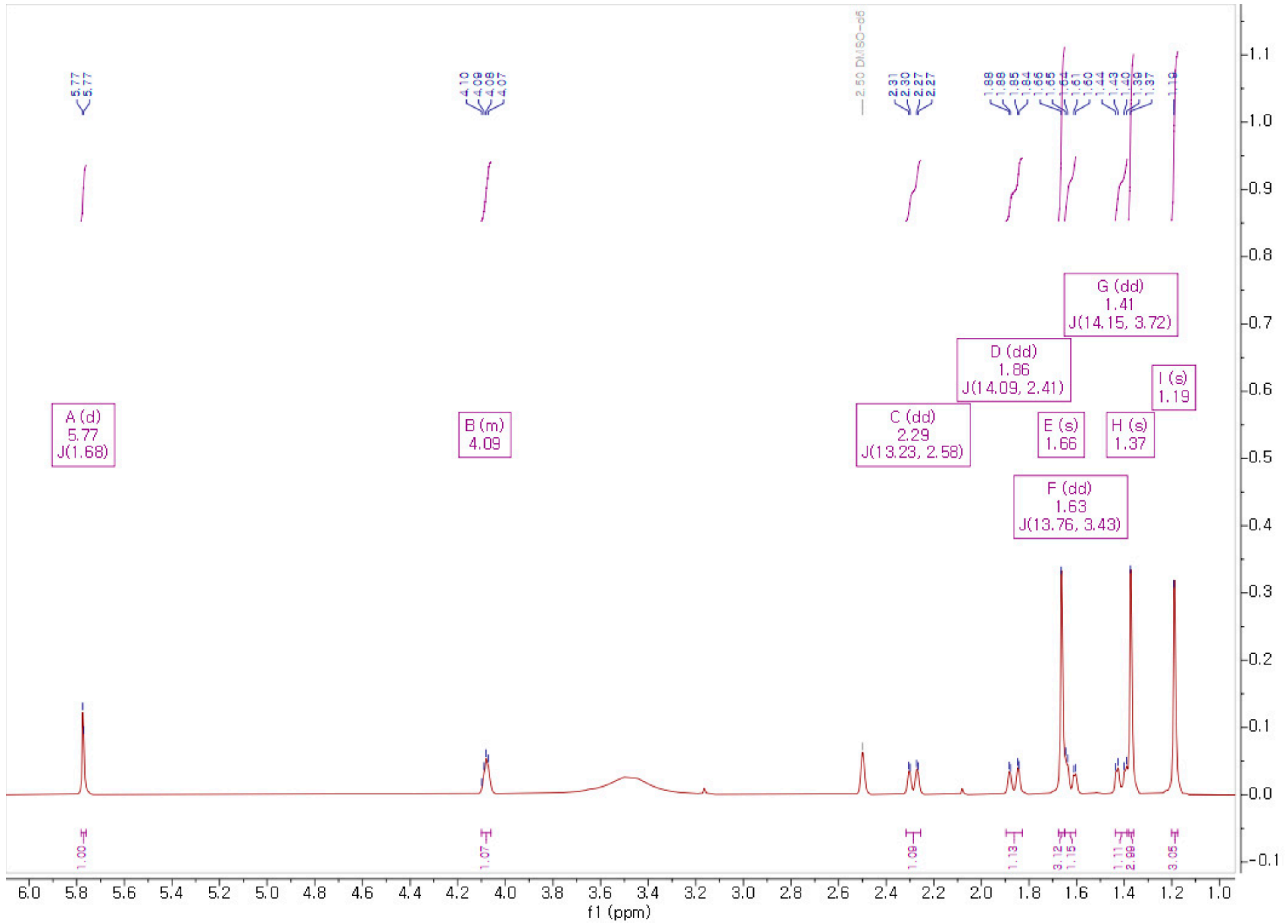

Figure S28. The ^{1}H NMR spectrum of compound **13** (DMSO-d_6, 400 MHz).

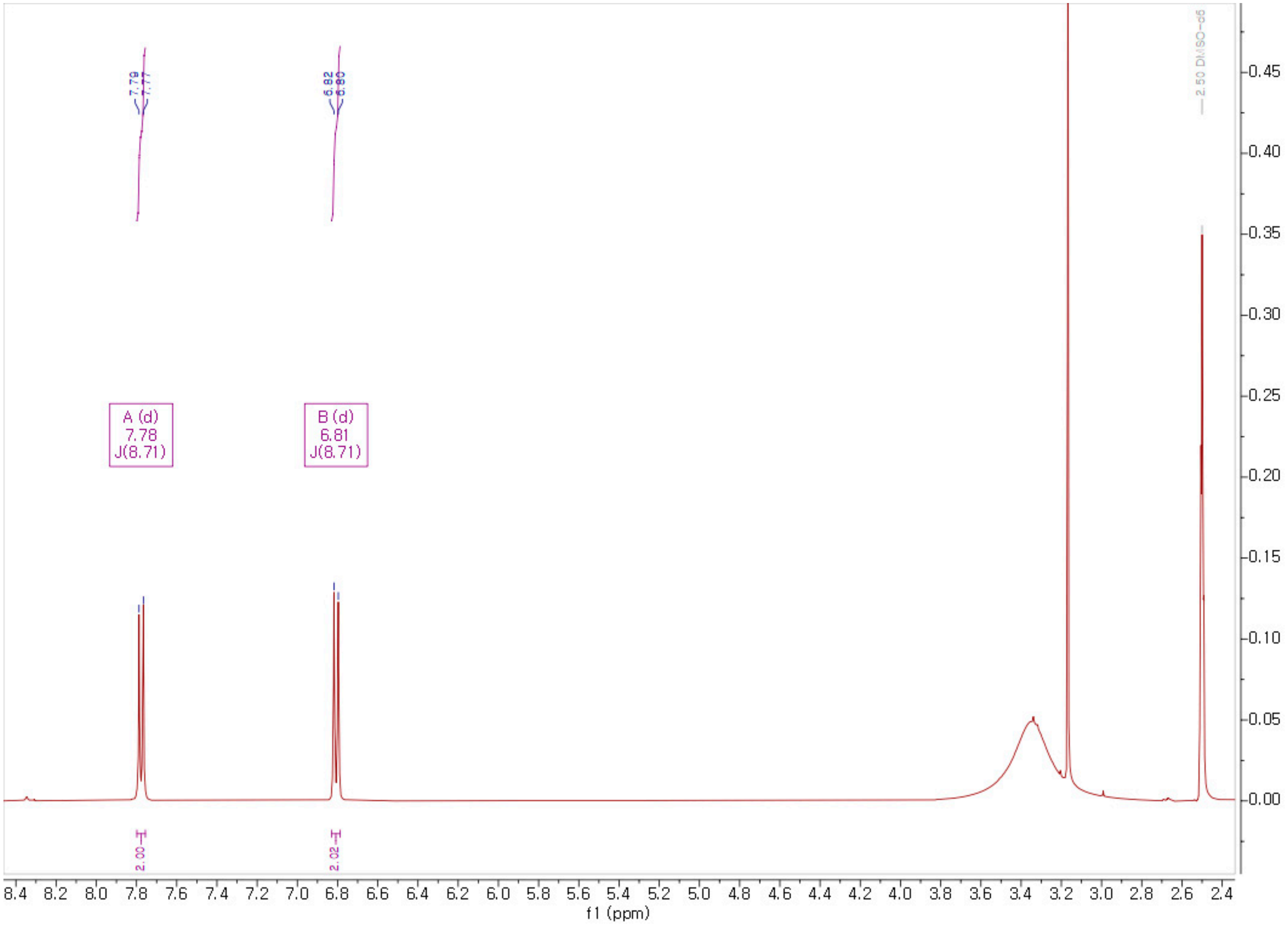

Figure S29. The ^{1}H NMR spectrum of compound **14** (DMSO-d_6, 400 MHz).

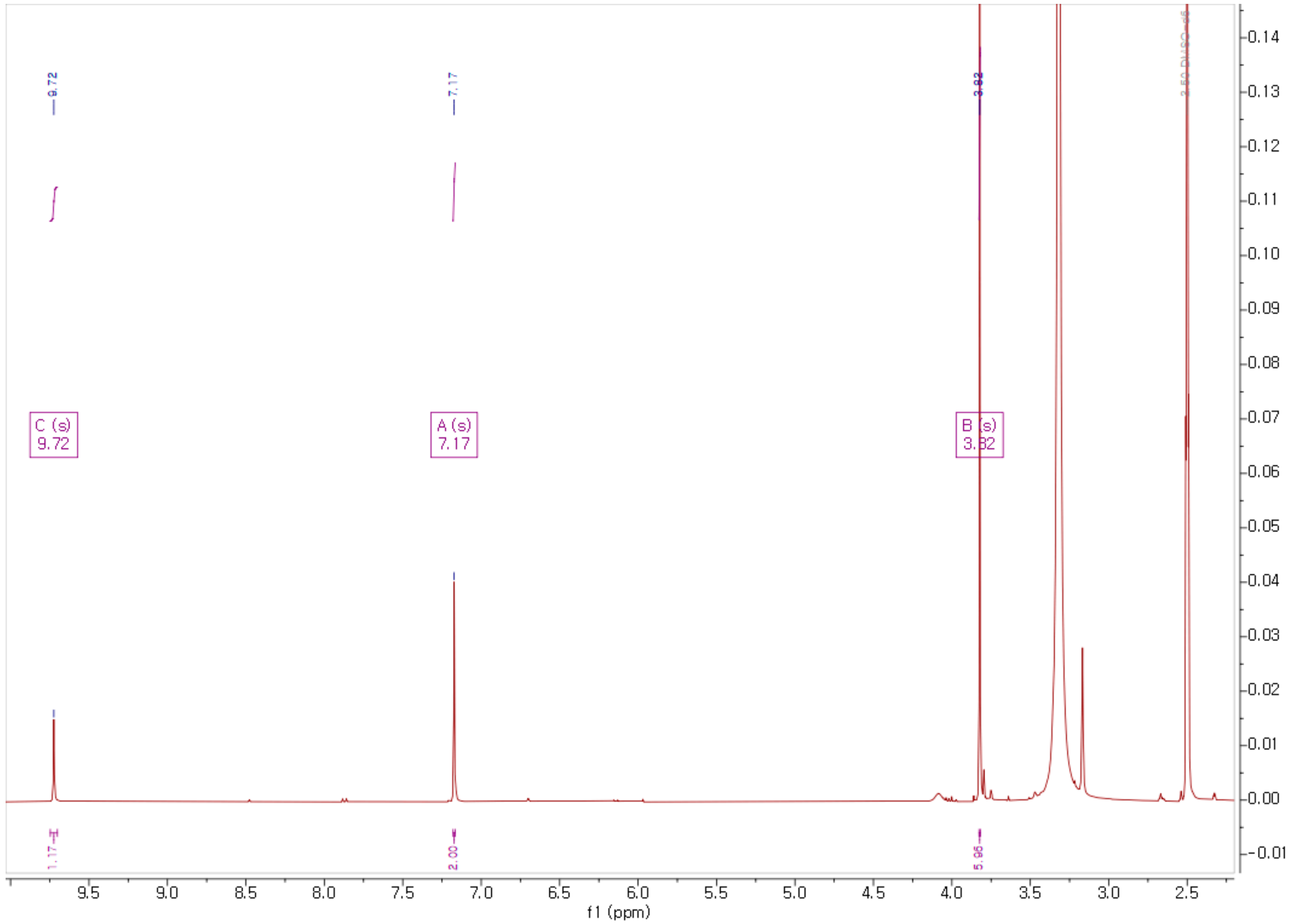

Figure S30. The ^{1}H NMR spectrum of compound **15** (DMSO-d_6, 400 MHz).

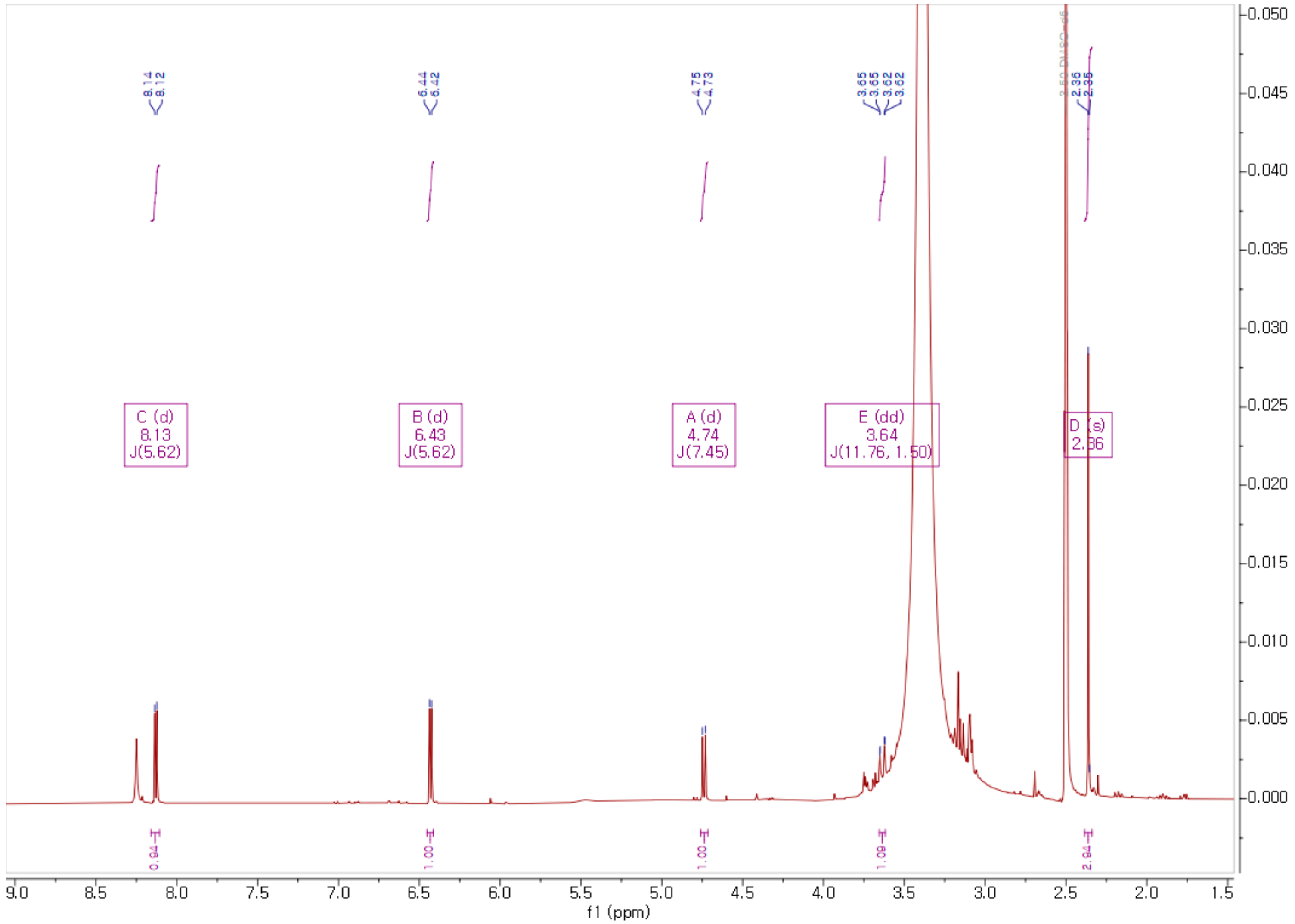

Figure S31. The ^{1}H NMR spectrum of compound **16** (DMSO-d_6, 400 MHz).

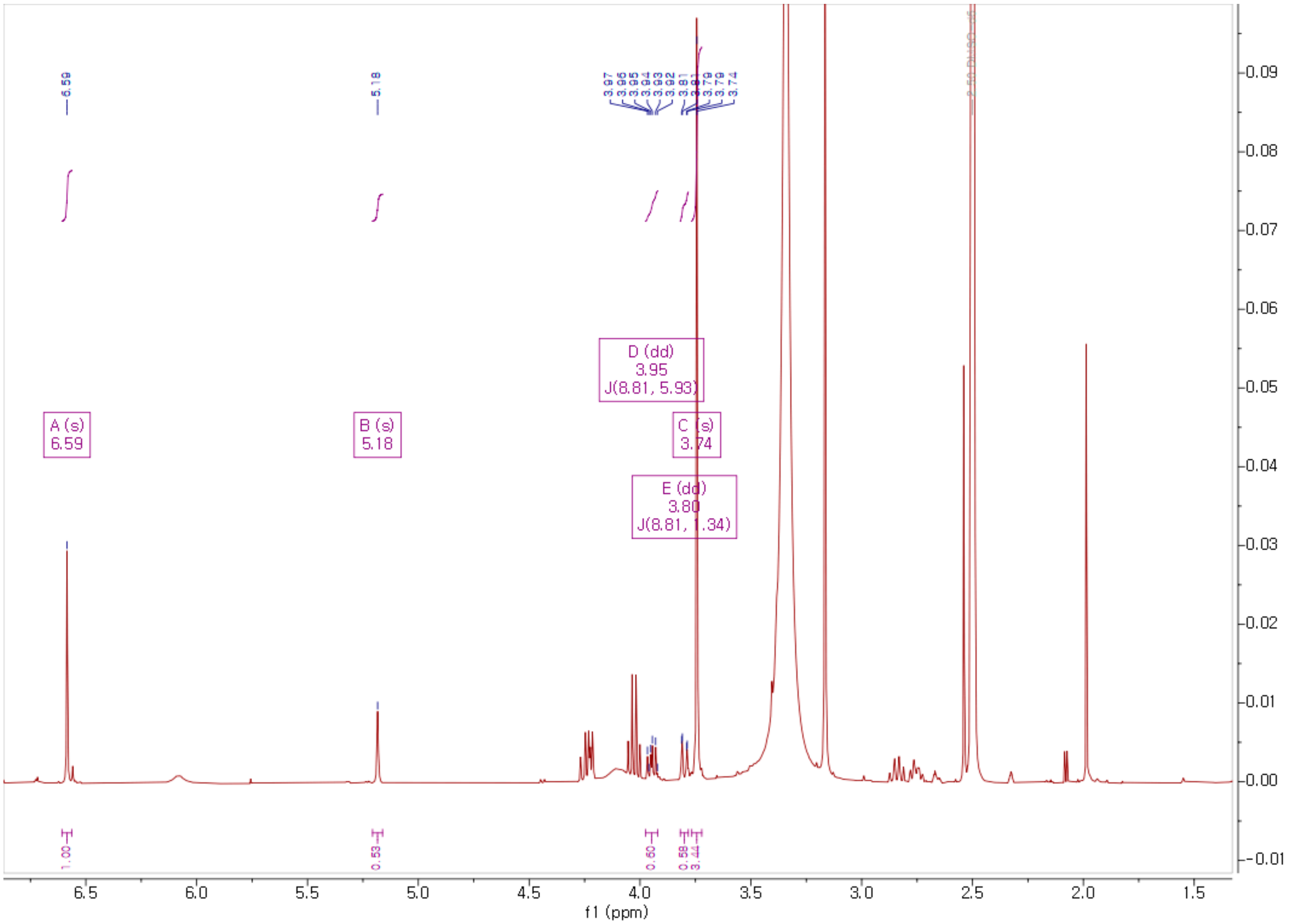

Figure S32. The ^{1}H NMR spectrum of compound **17** (DMSO-d_6, 400 MHz).

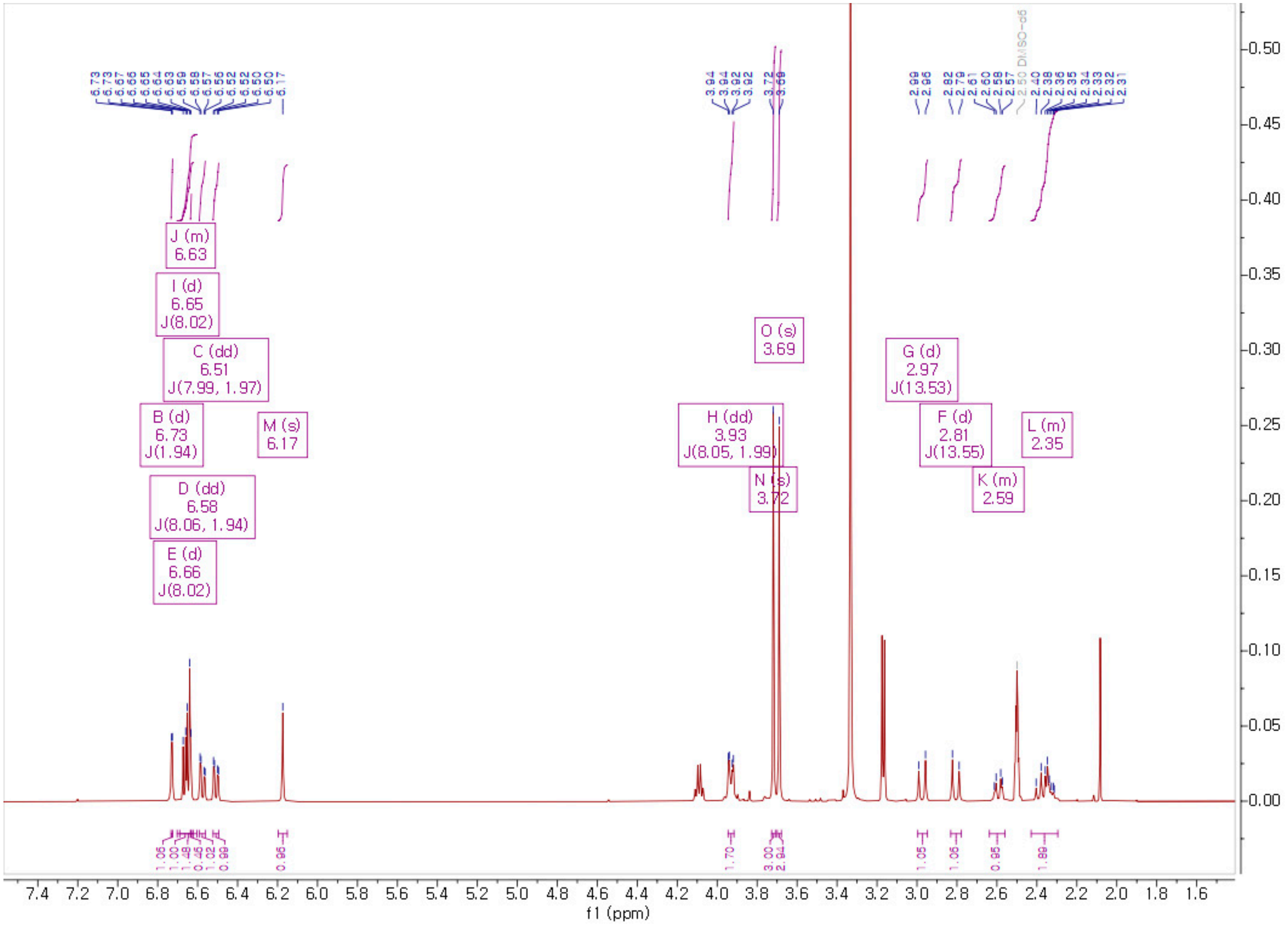

Figure S33. The ^{1}H NMR spectrum of compound **18** (DMSO-d_6, 400 MHz).

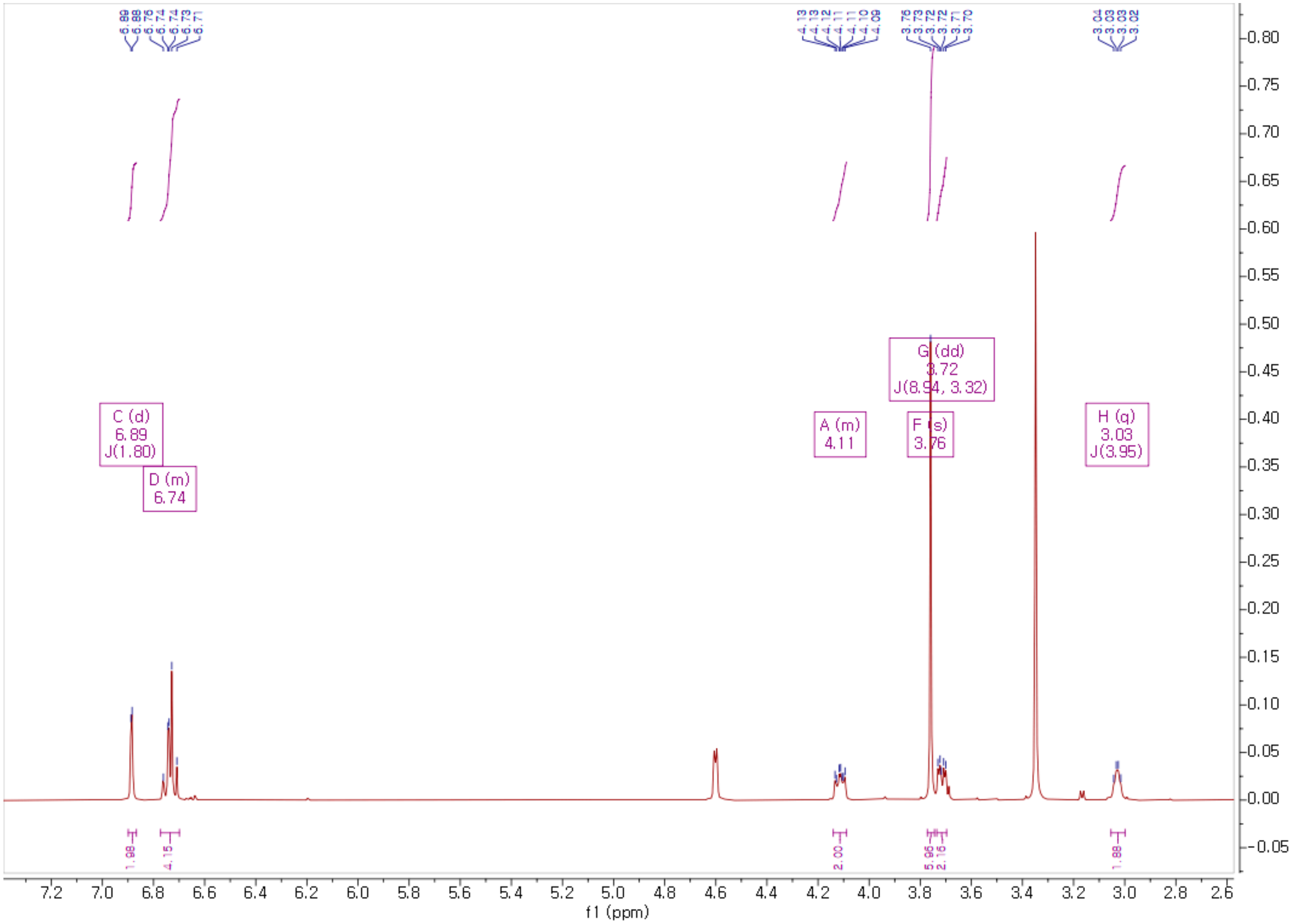

Figure S34. The ^{1}H NMR spectrum of compound **19** (DMSO-d_6, 400 MHz).

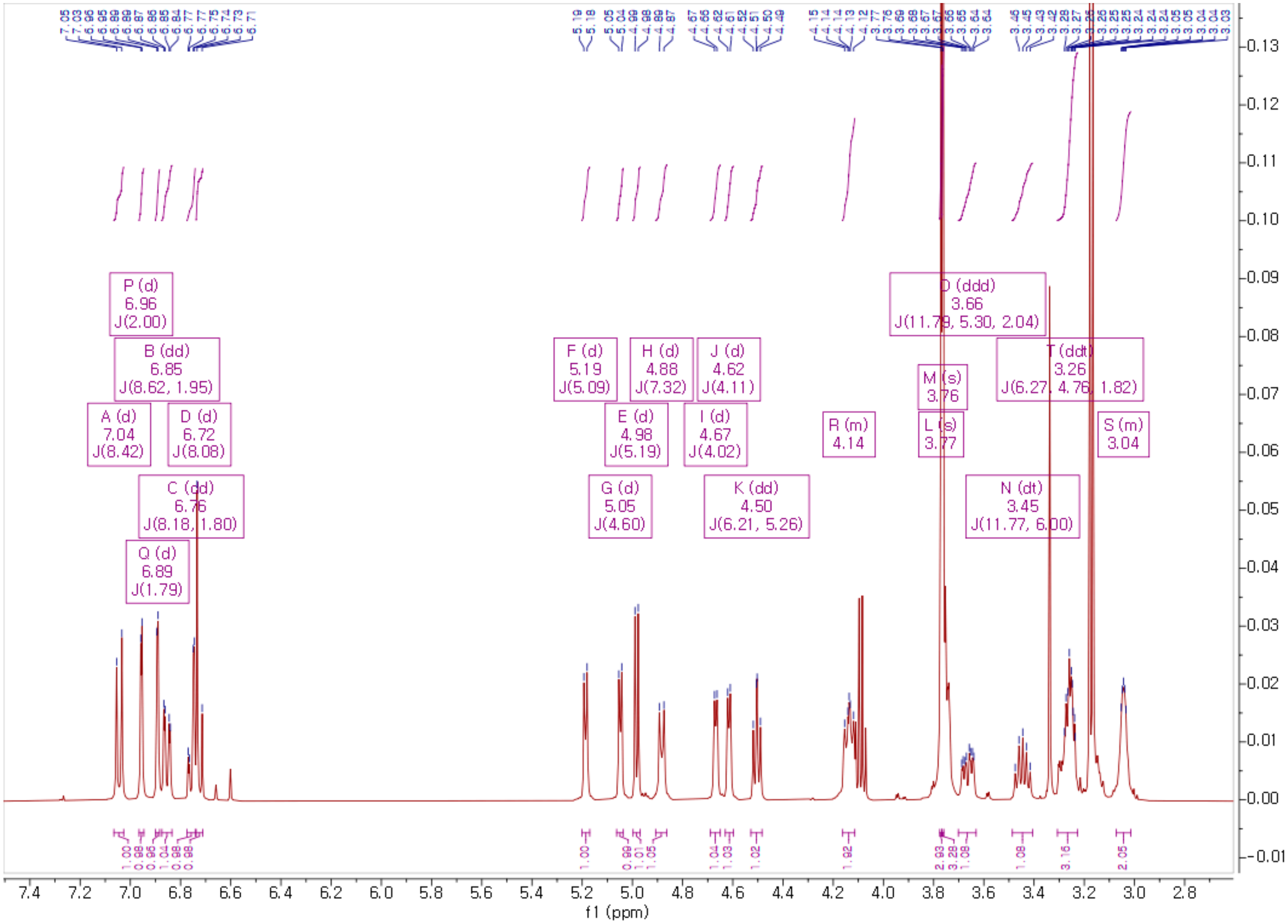

Figure S35. The ^{1}H NMR spectrum of compound **20** (DMSO-d_6, 400 MHz).

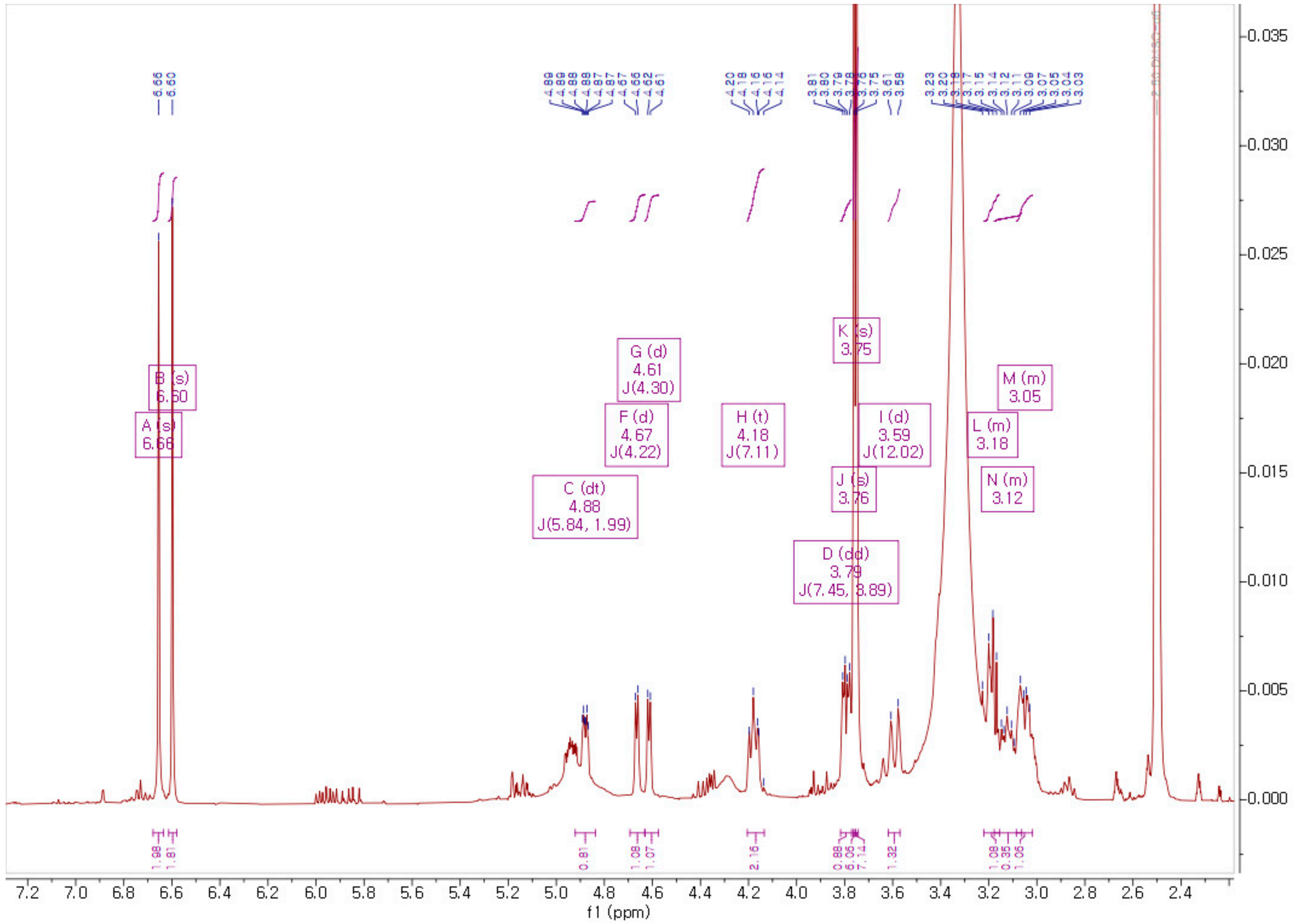

Figure S36. The ^{1}H NMR spectrum of compound **21** (DMSO-d_6, 400 MHz).

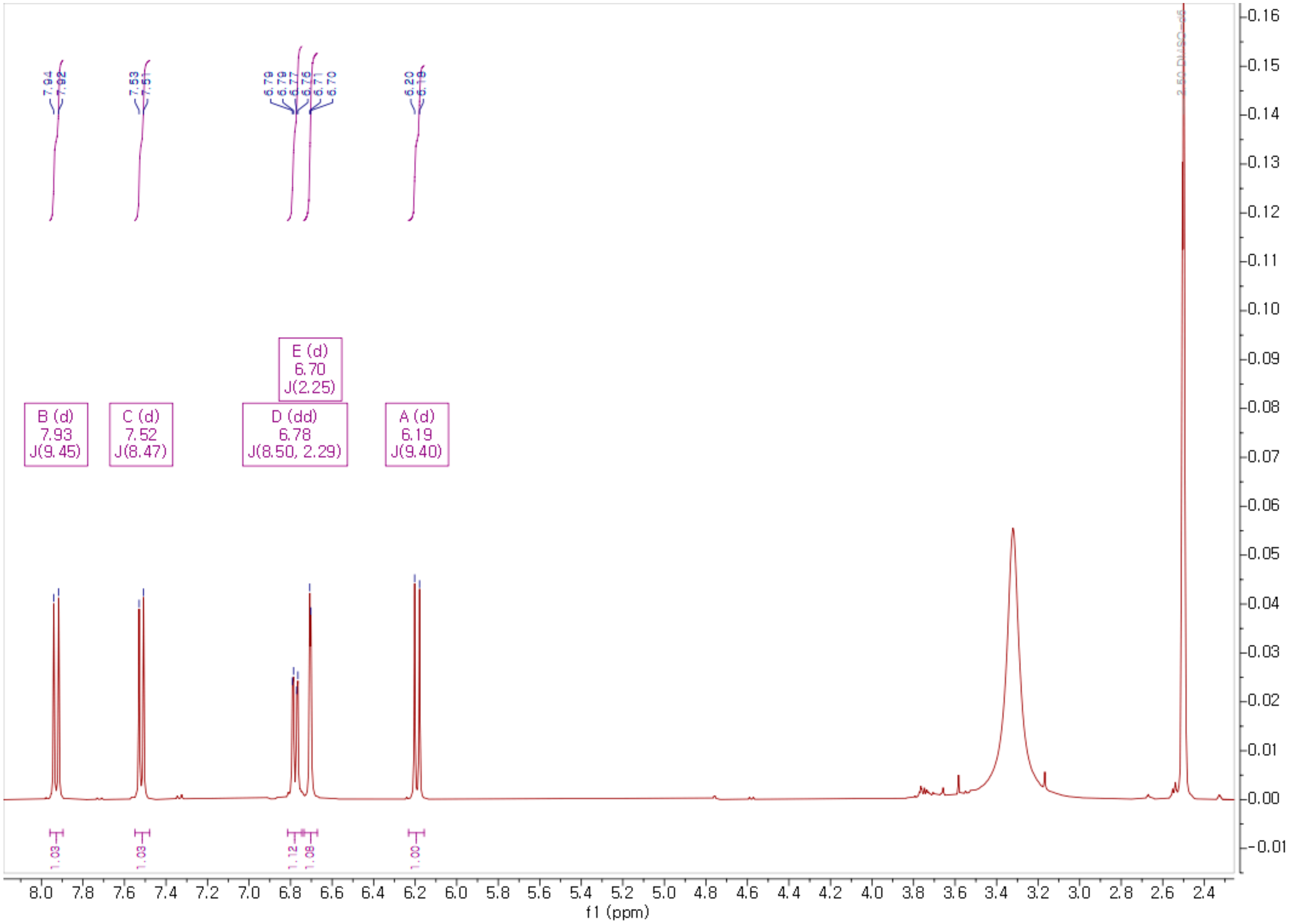

Figure S37. The ^{1}H NMR spectrum of compound **22** (DMSO-d_6, 400 MHz).

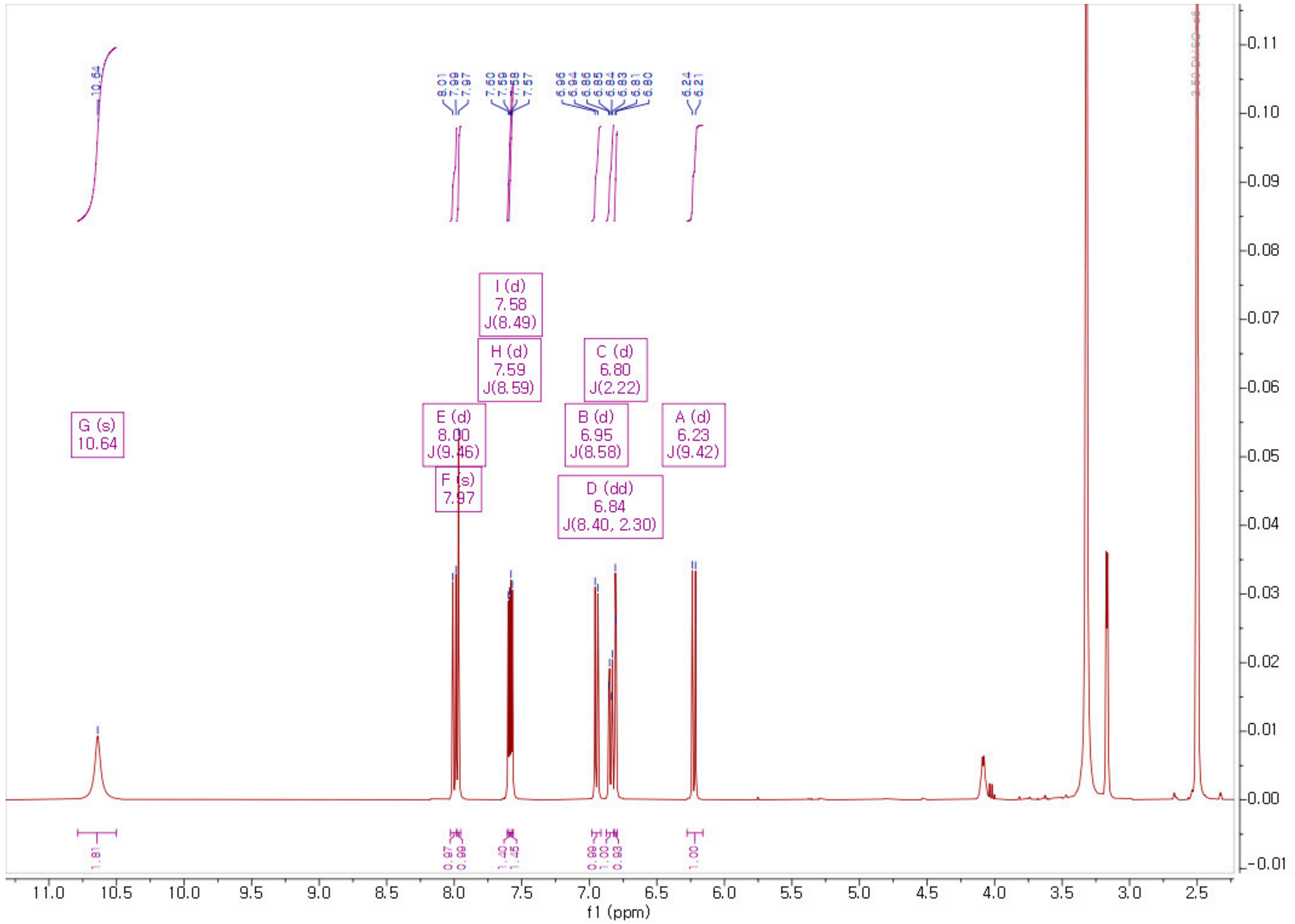

Figure S38. The ^{1}H NMR spectrum of compound **23** (Acetone-d_6, 400 MHz).

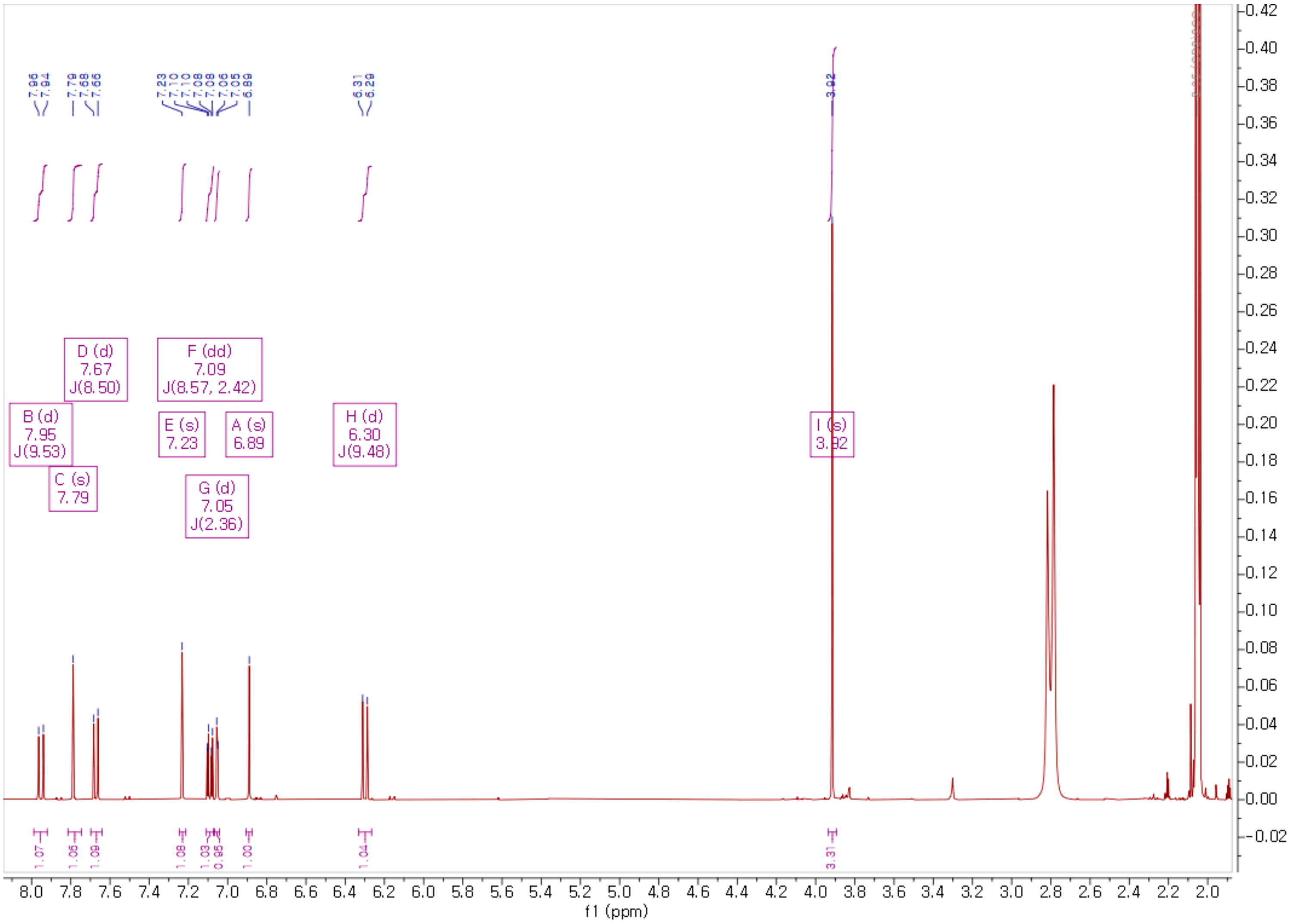

Figure S39. The ^{1}H NMR spectrum of compound **24** (DMSO-d_6, 400 MHz).

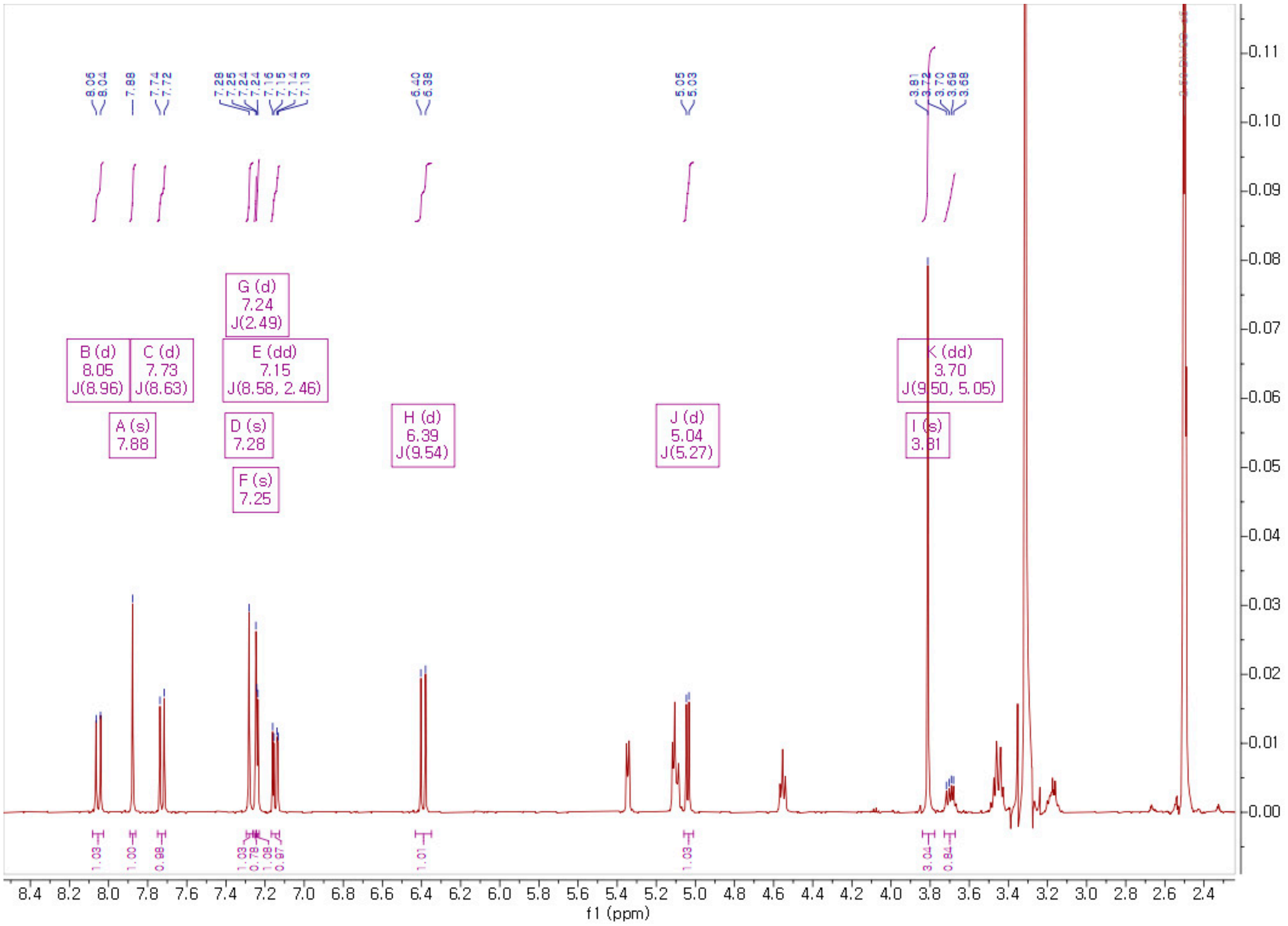

Figure S40. The ^{1}H NMR spectrum of compound **25** (DMSO-d_6, 400 MHz).

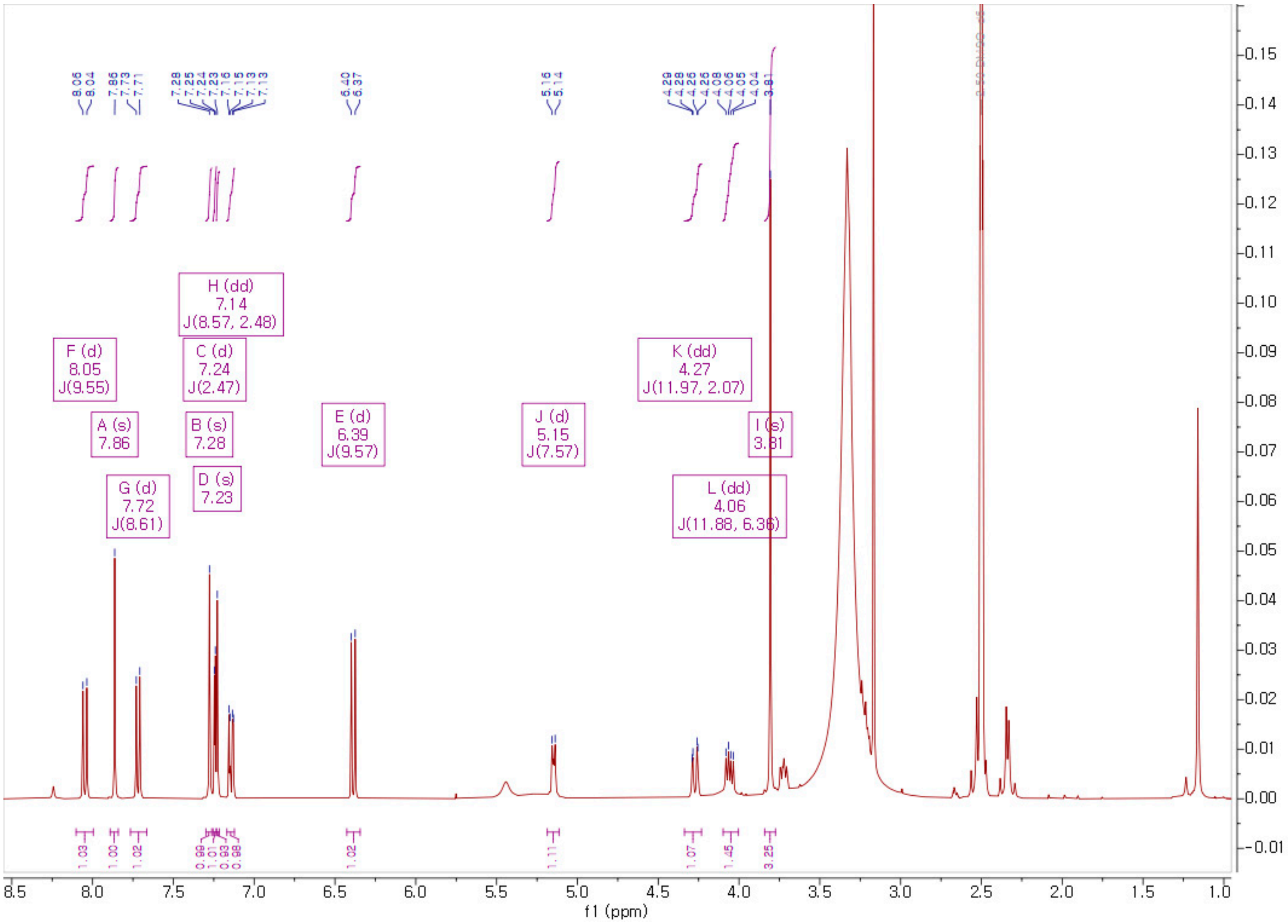

Figure S41. The ^{1}H NMR spectrum of compound **26** (DMSO-d_6, 400 MHz).

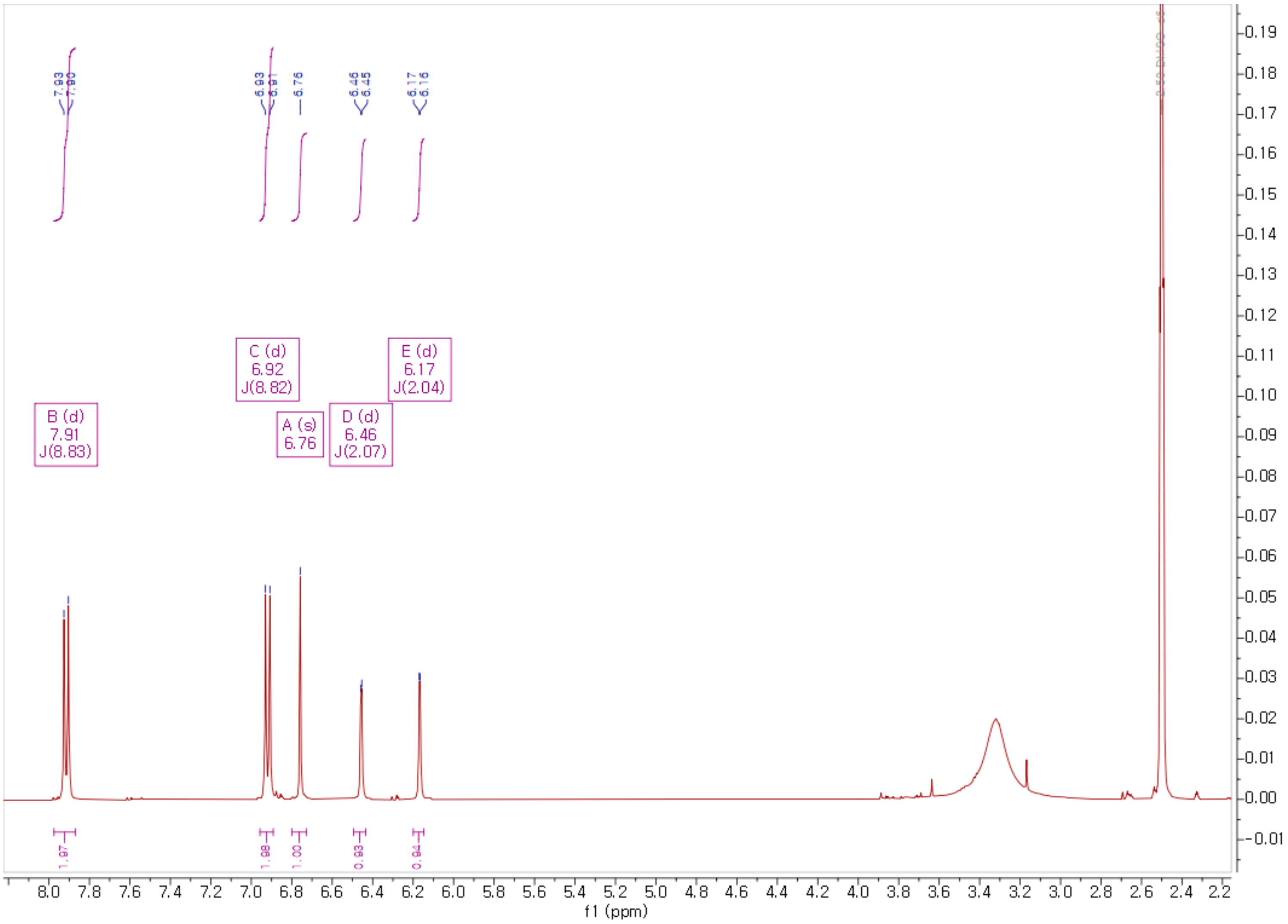

Figure S42. The ^{1}H NMR spectrum of compound **27** (DMSO-d_6, 400 MHz).

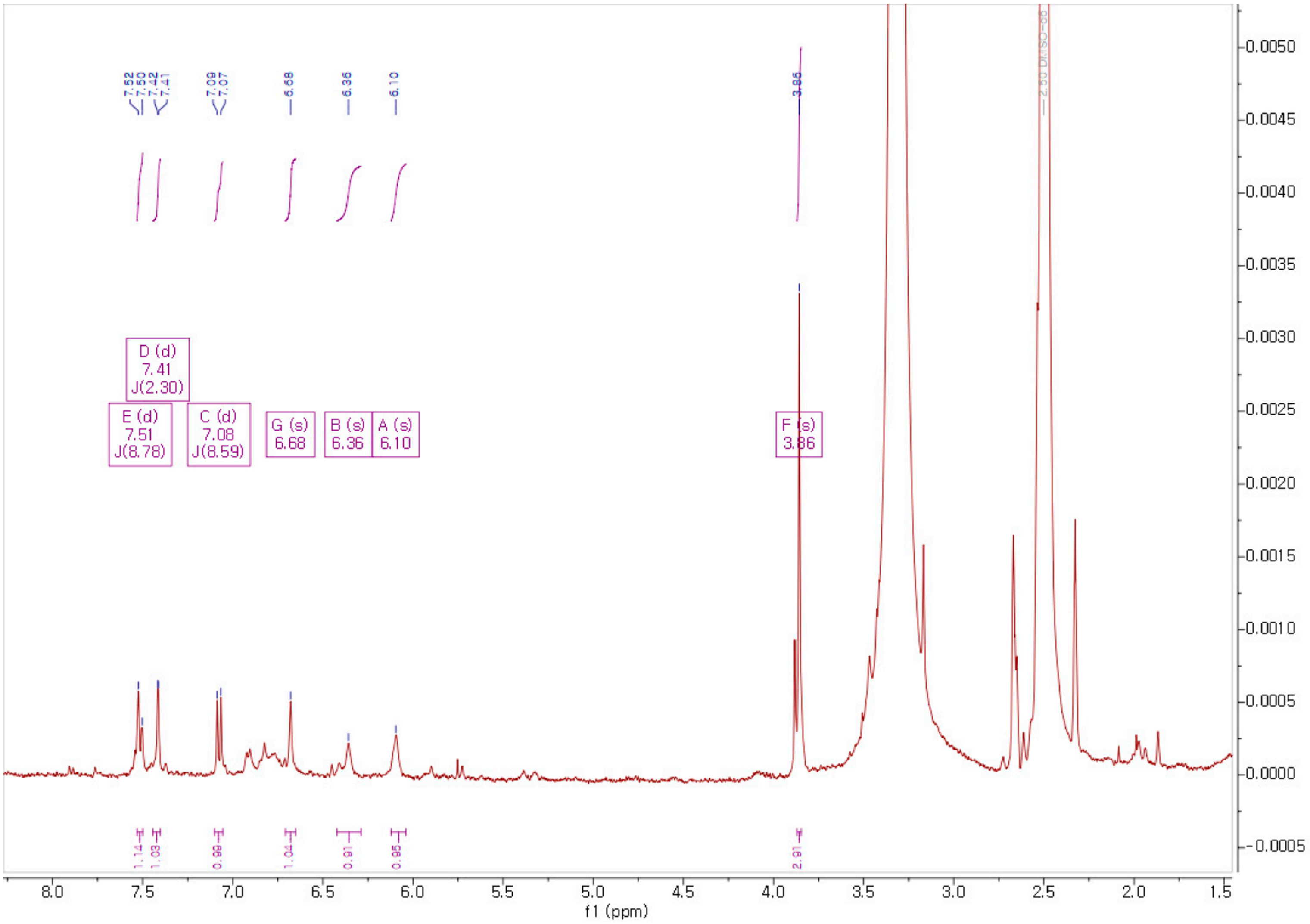

Figure S43. The ^{1}H NMR spectrum of compound **28** (DMSO-d_6, 400 MHz).

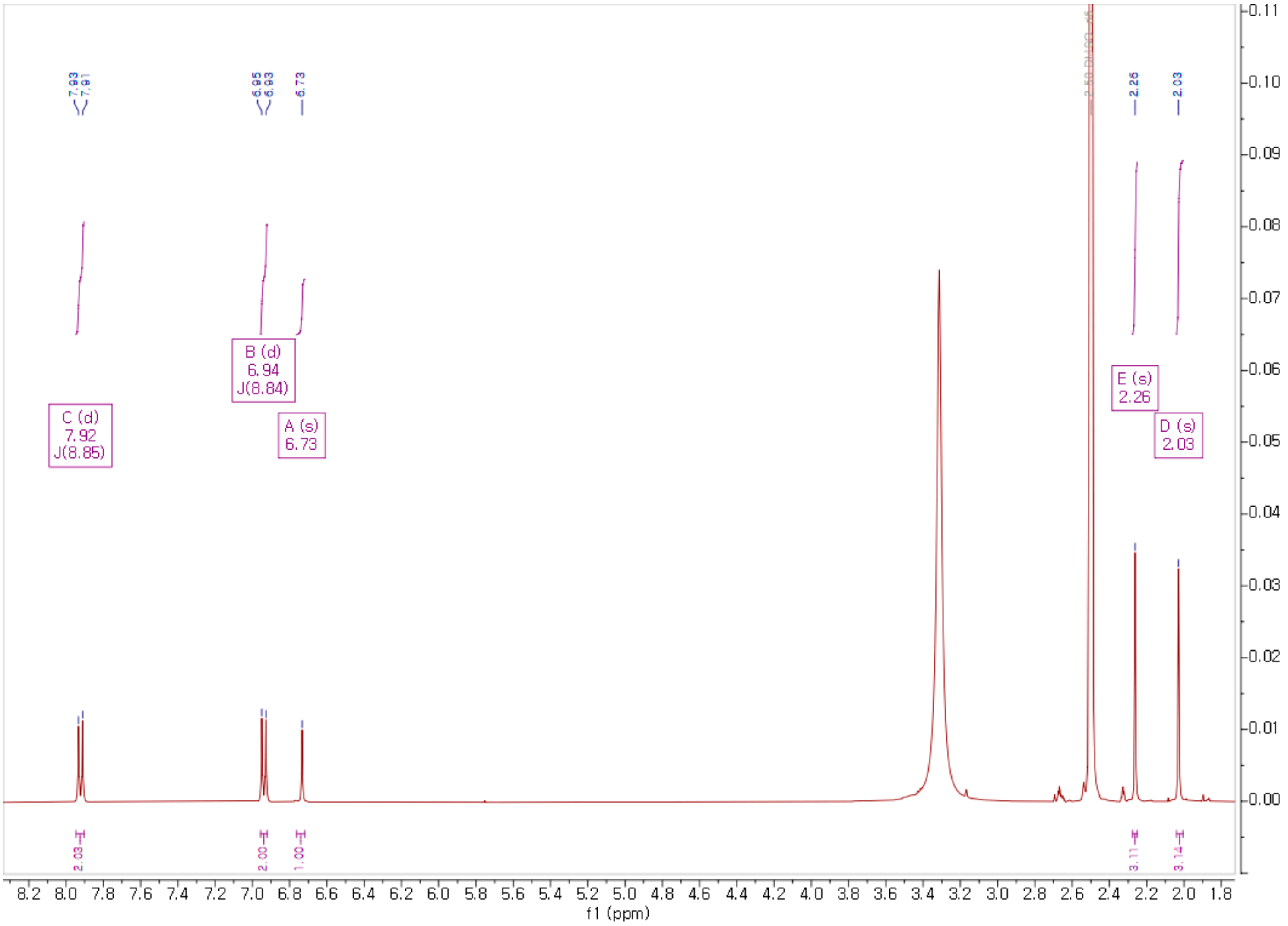

Figure S44. The ^{1}H NMR spectrum of compound **29** (DMSO-d_6, 400 MHz).

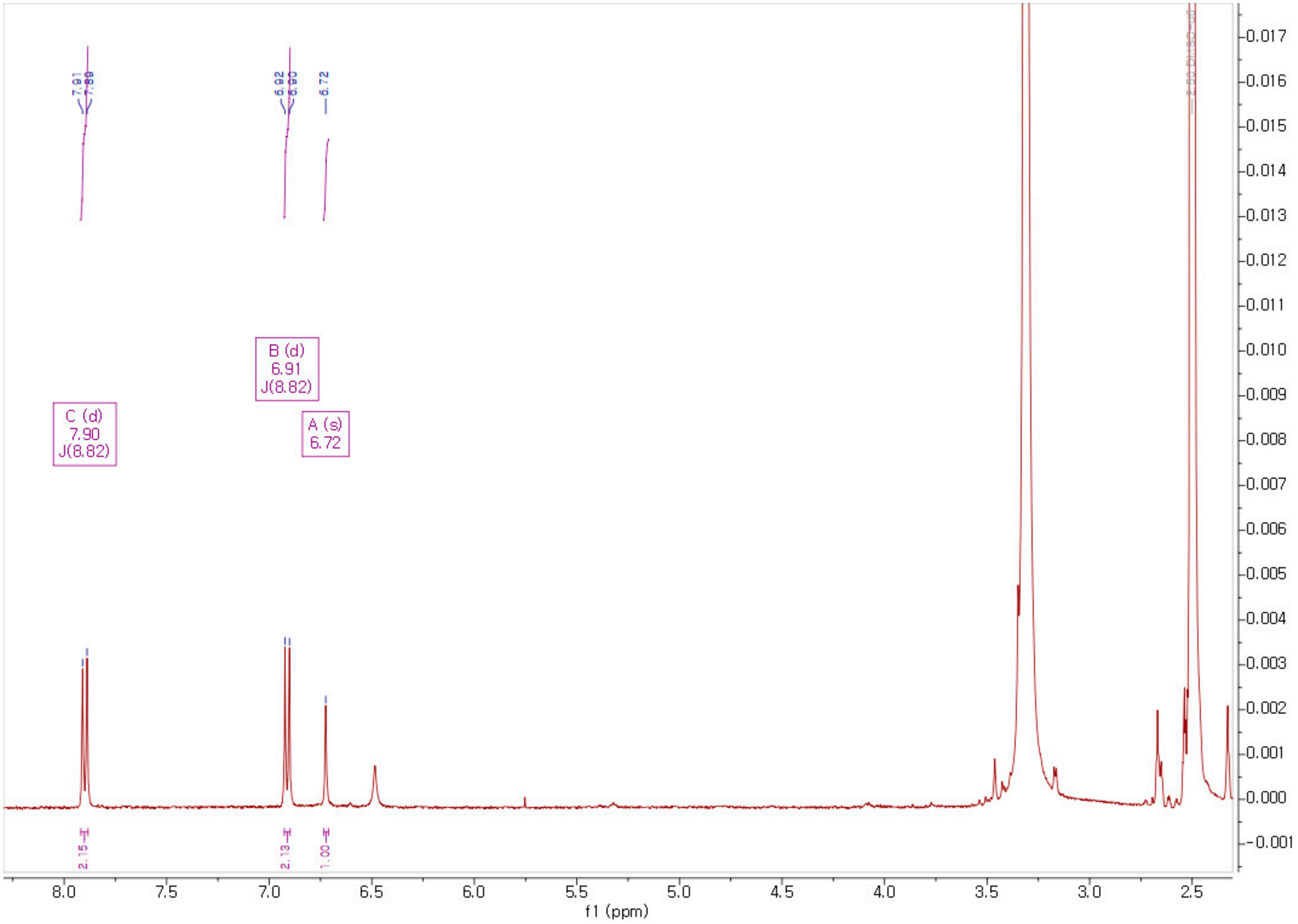

Figure S45. The ^{1}H NMR spectrum of compound **30** (DMSO-d_6, 400 MHz).

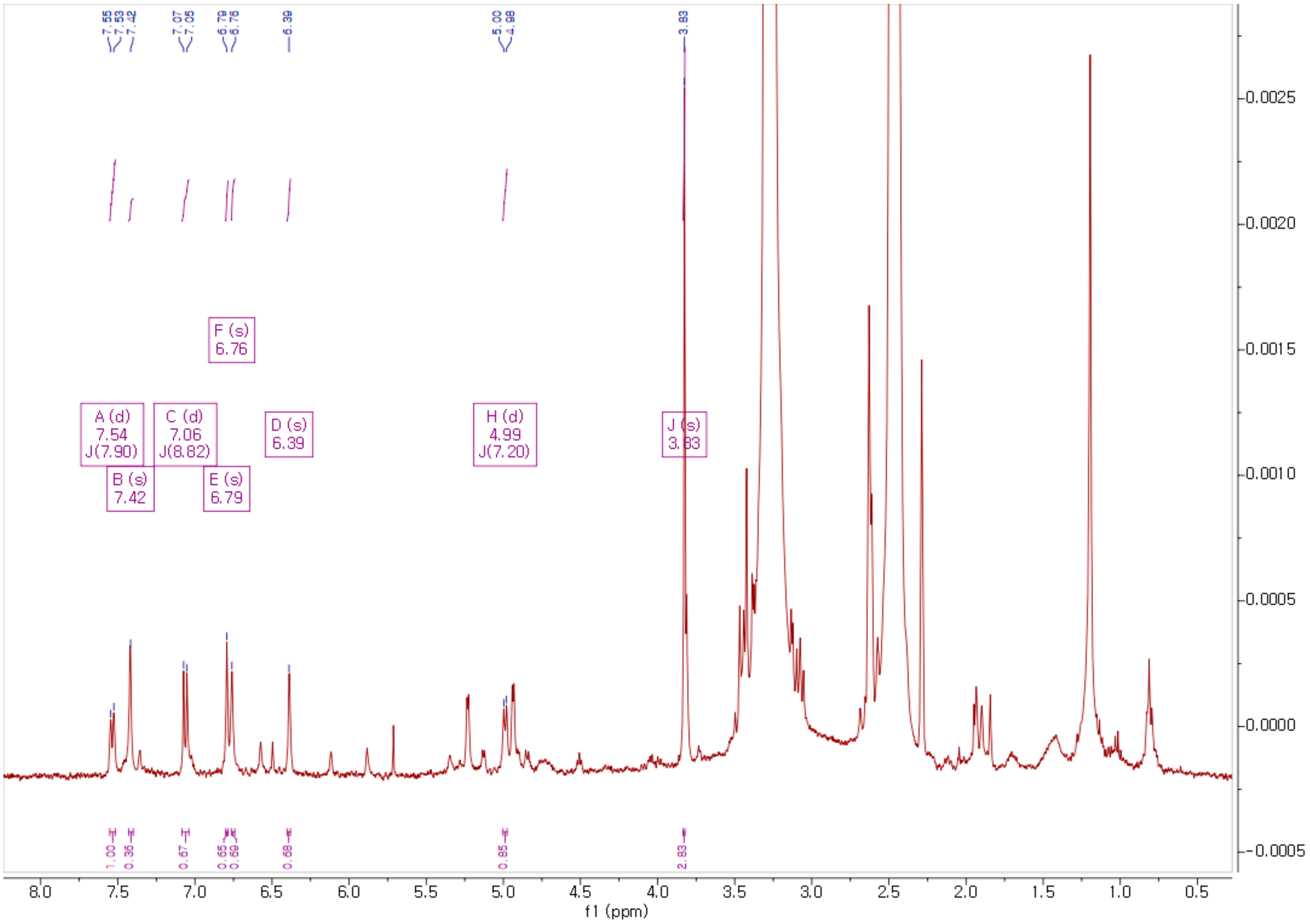

Figure S46. The ^{1}H NMR spectrum of compound **31** (DMSO-d_6, 400 MHz).

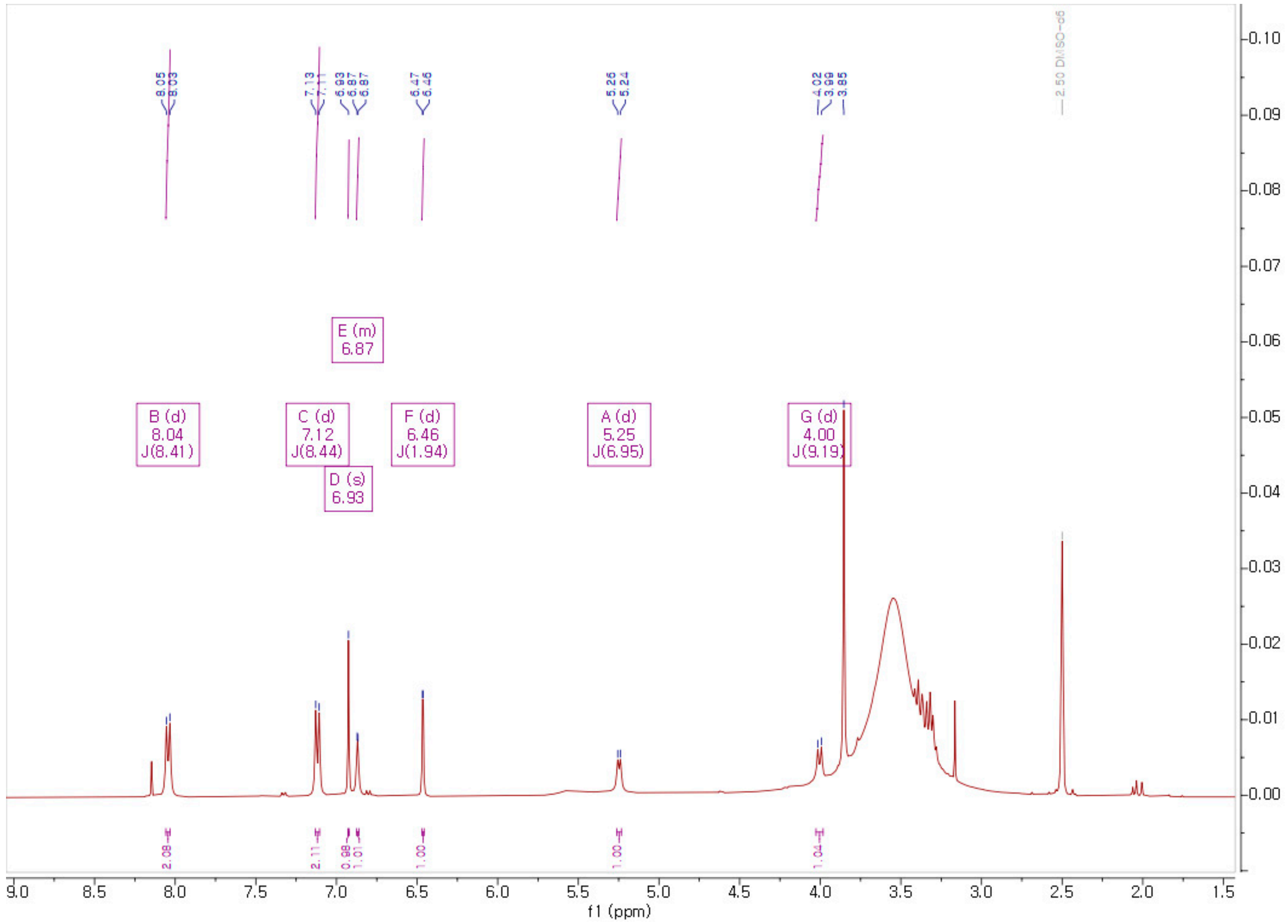

Figure S47. The ^{1}H NMR spectrum of compound **32** (DMSO-d_6, 400 MHz).

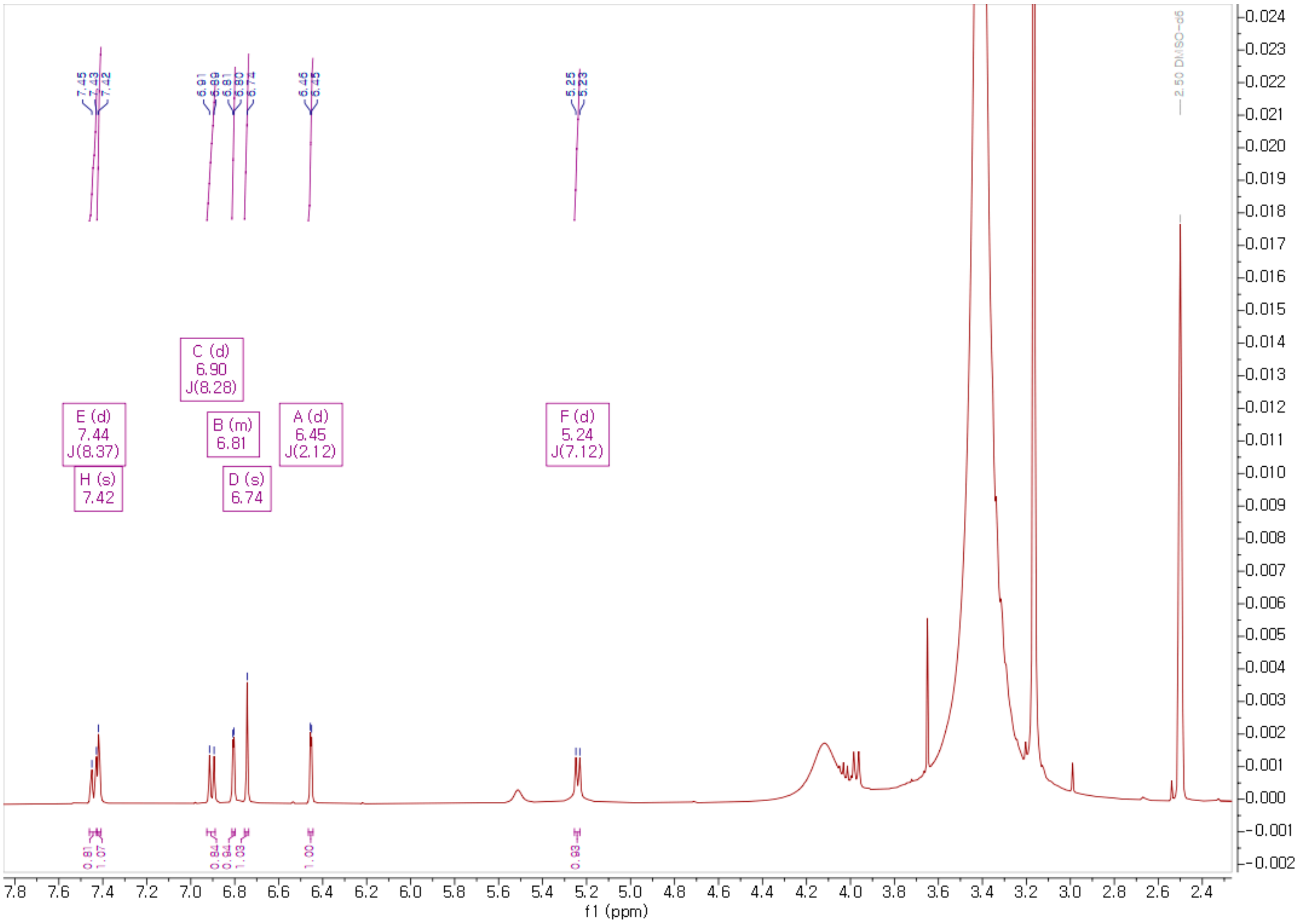

Figure S48. The ^{1}H NMR spectrum of compound **33** (DMSO-d_6, 400 MHz).

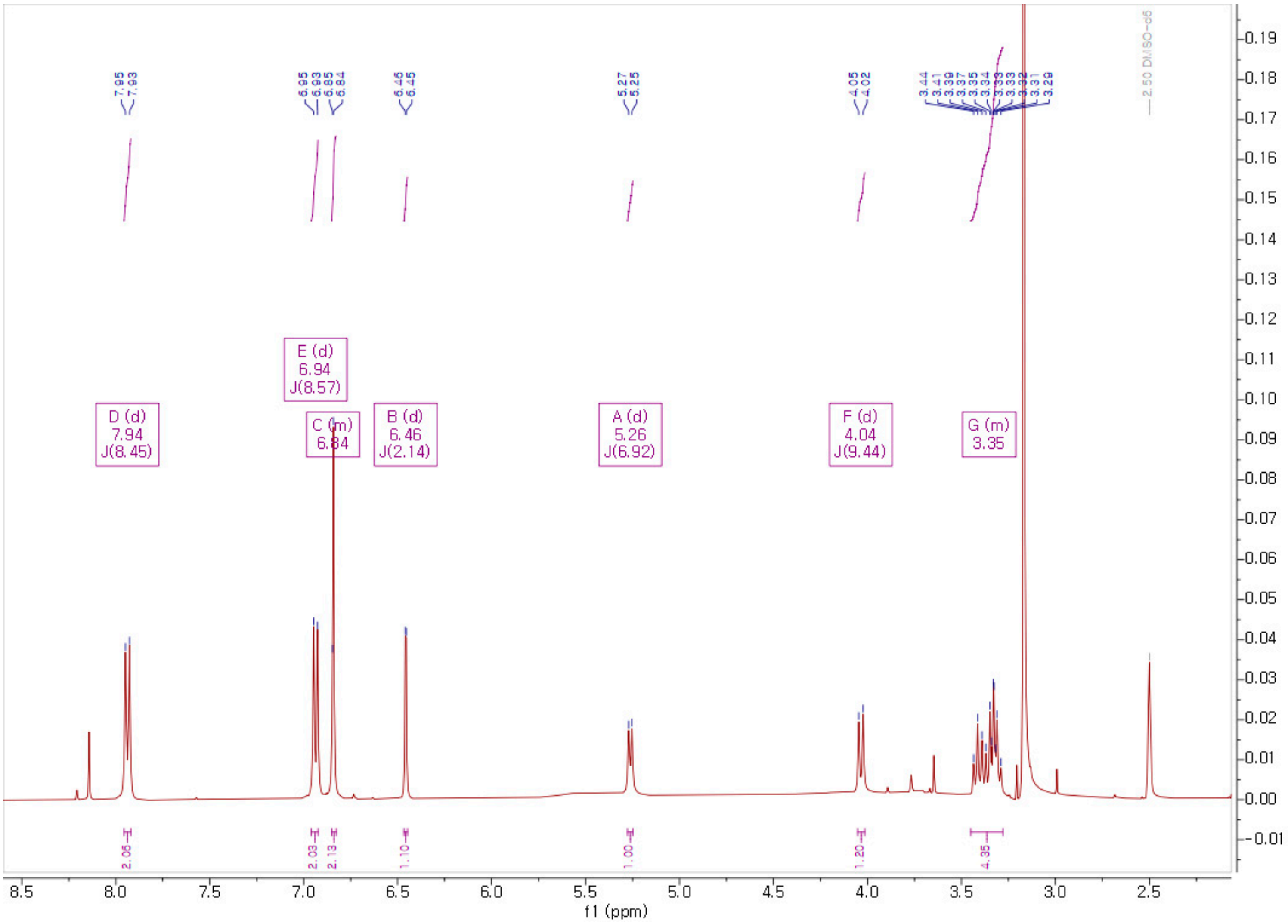

Figure S49. The ^{1}H NMR spectrum of compound **34** (DMSO-d_6, 400 MHz).

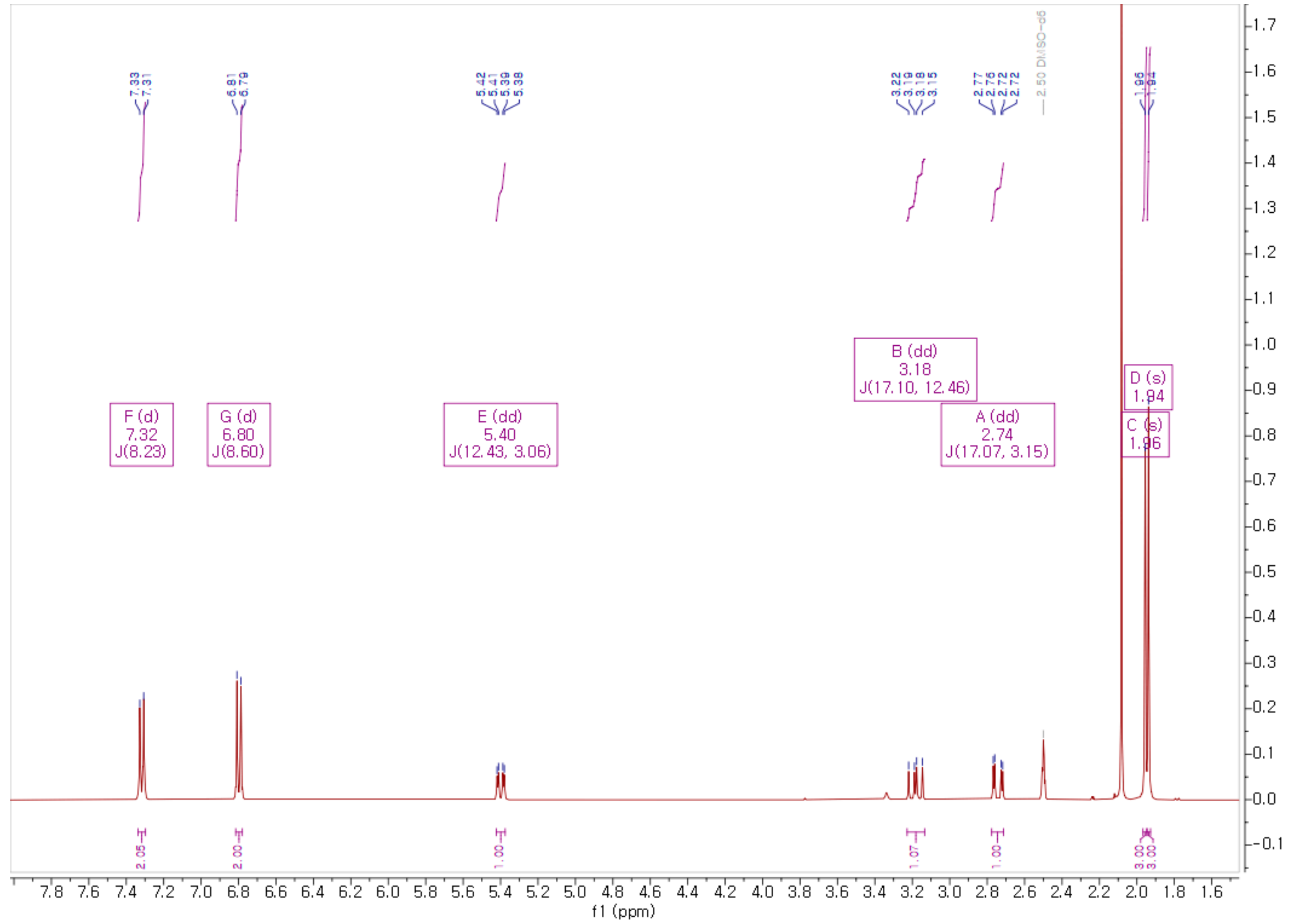

Figure S50. The ^{1}H NMR spectrum of compound **35** (DMSO-d_6, 400 MHz).

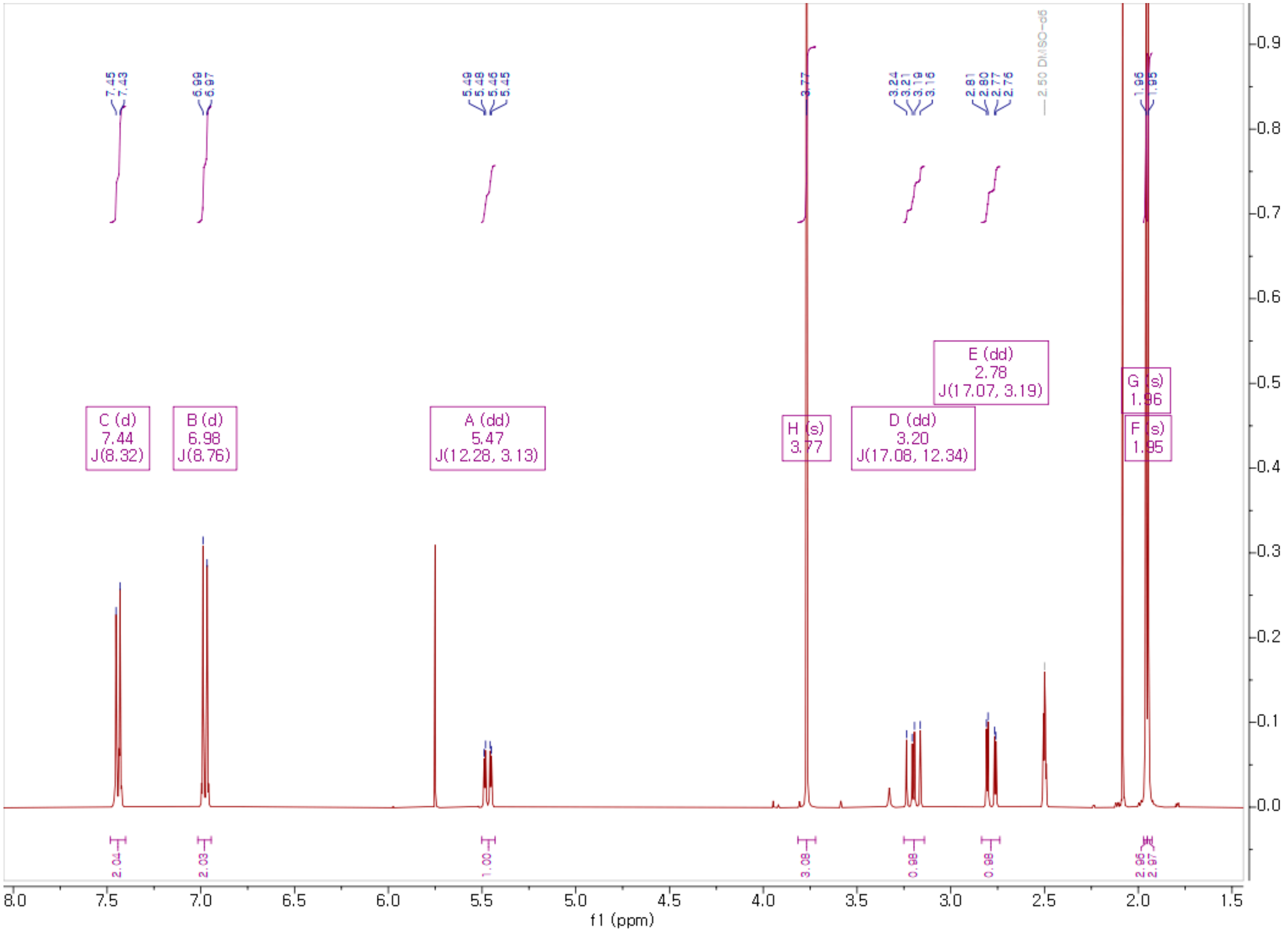

Figure S51. The ¹H NMR spectrum of compound **36** (DMSO-d_6, 400 MHz).

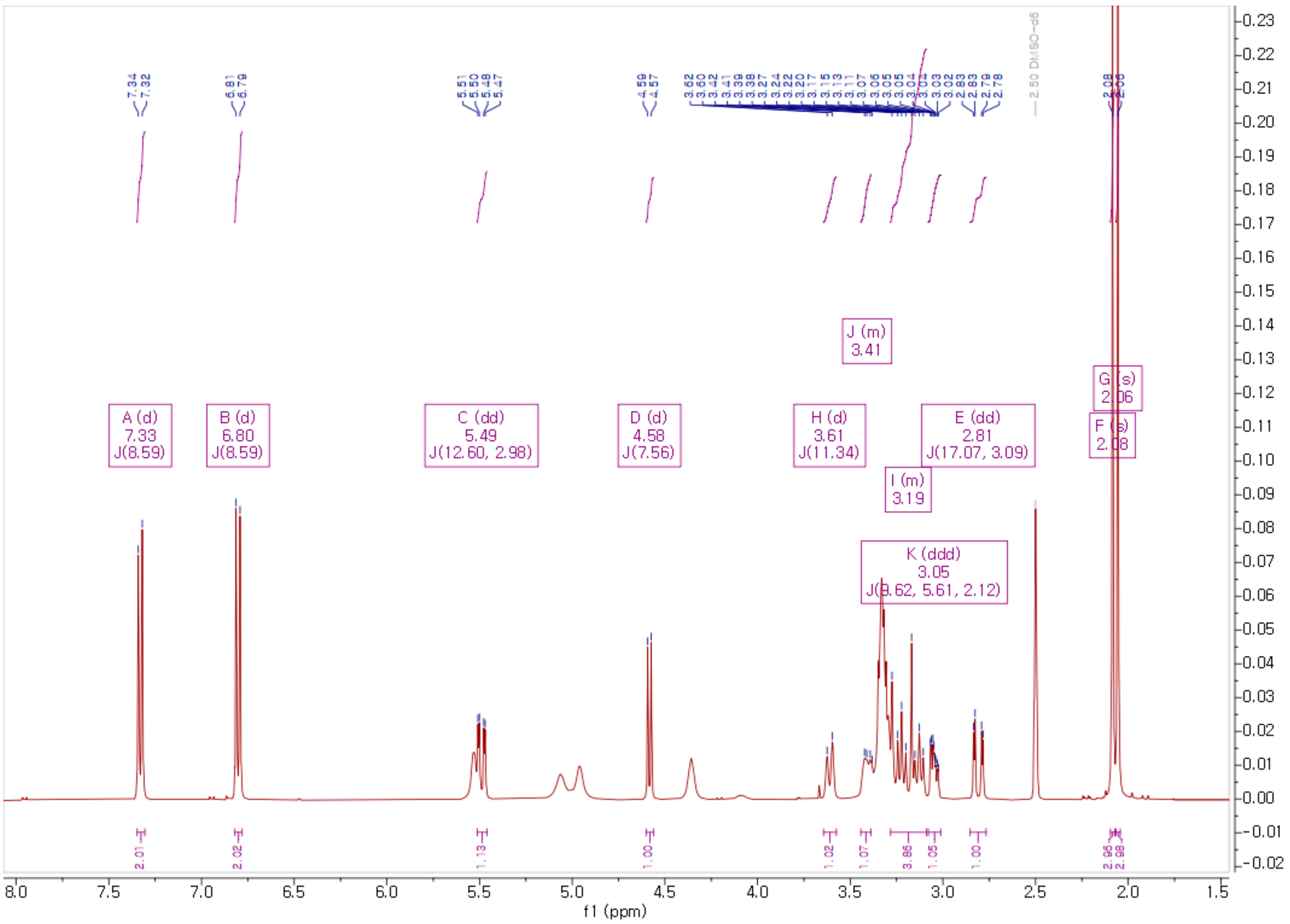

Figure S52. The ^{1}H NMR spectrum of compound **37** (DMSO-d_6, 400 MHz).

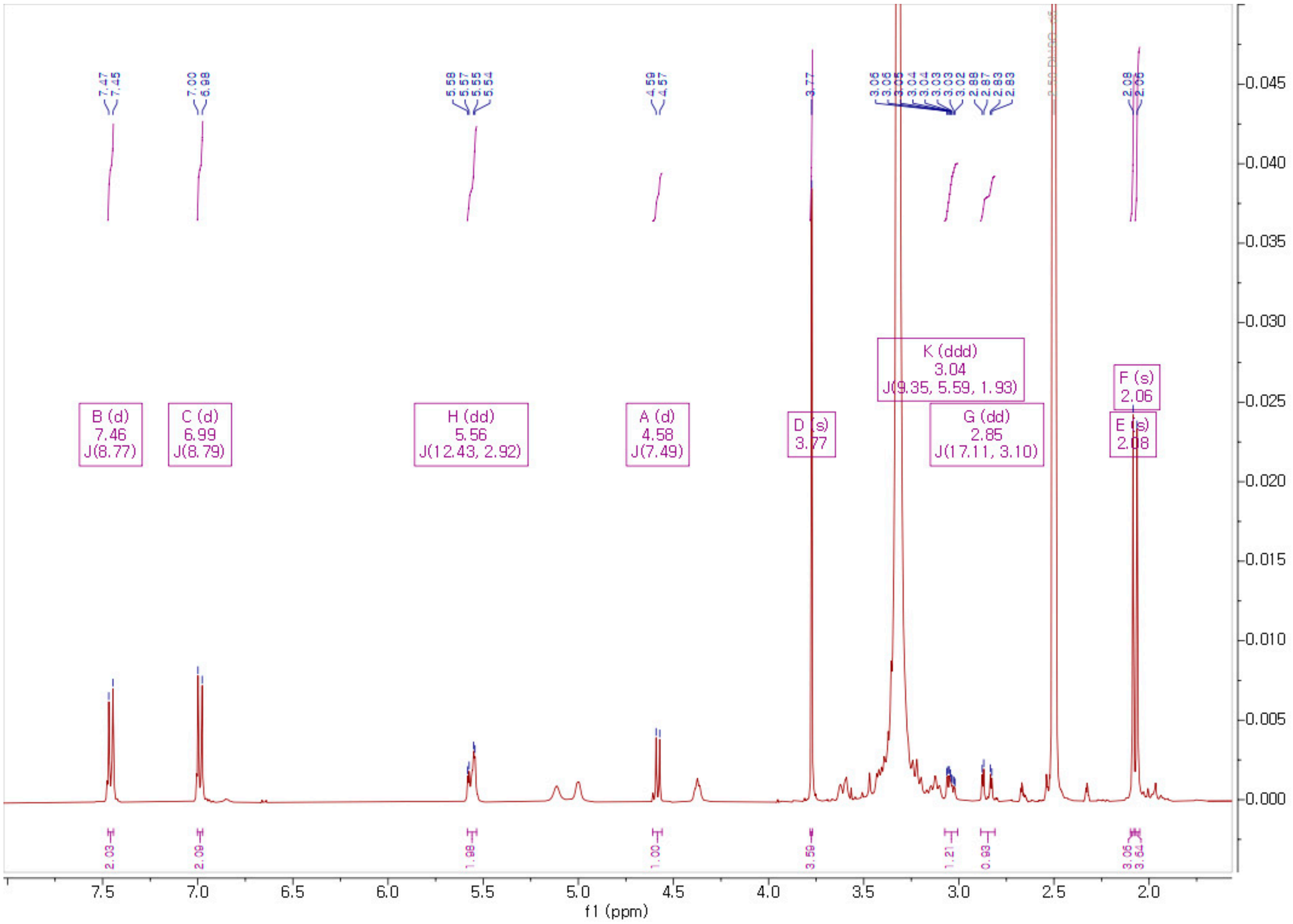

Figure S53. The ^{1}H NMR spectrum of compound **38** (DMSO-d_6, 400 MHz).

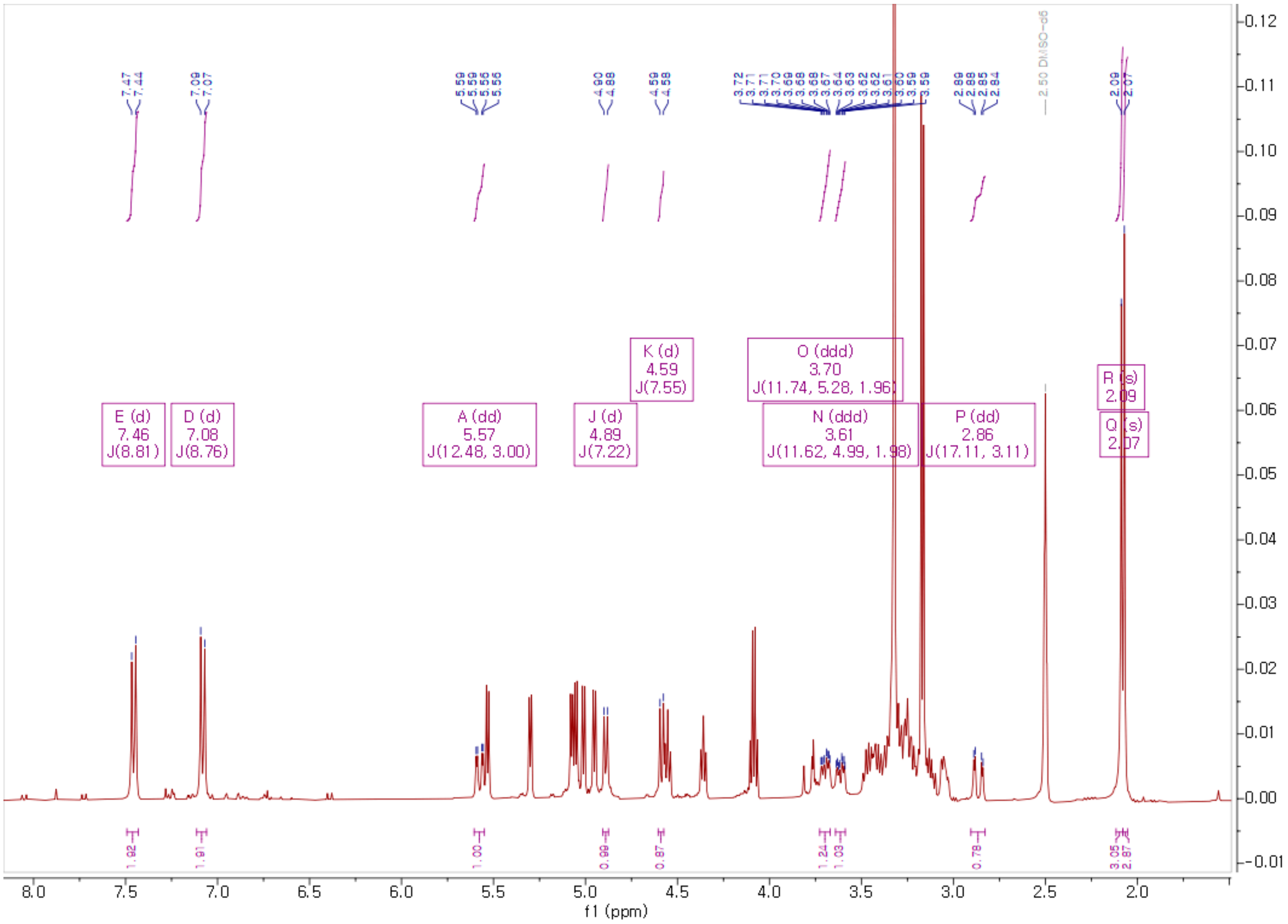

Figure S54. The ^{1}H NMR spectrum of compound **39** (DMSO-d_6, 400 MHz).

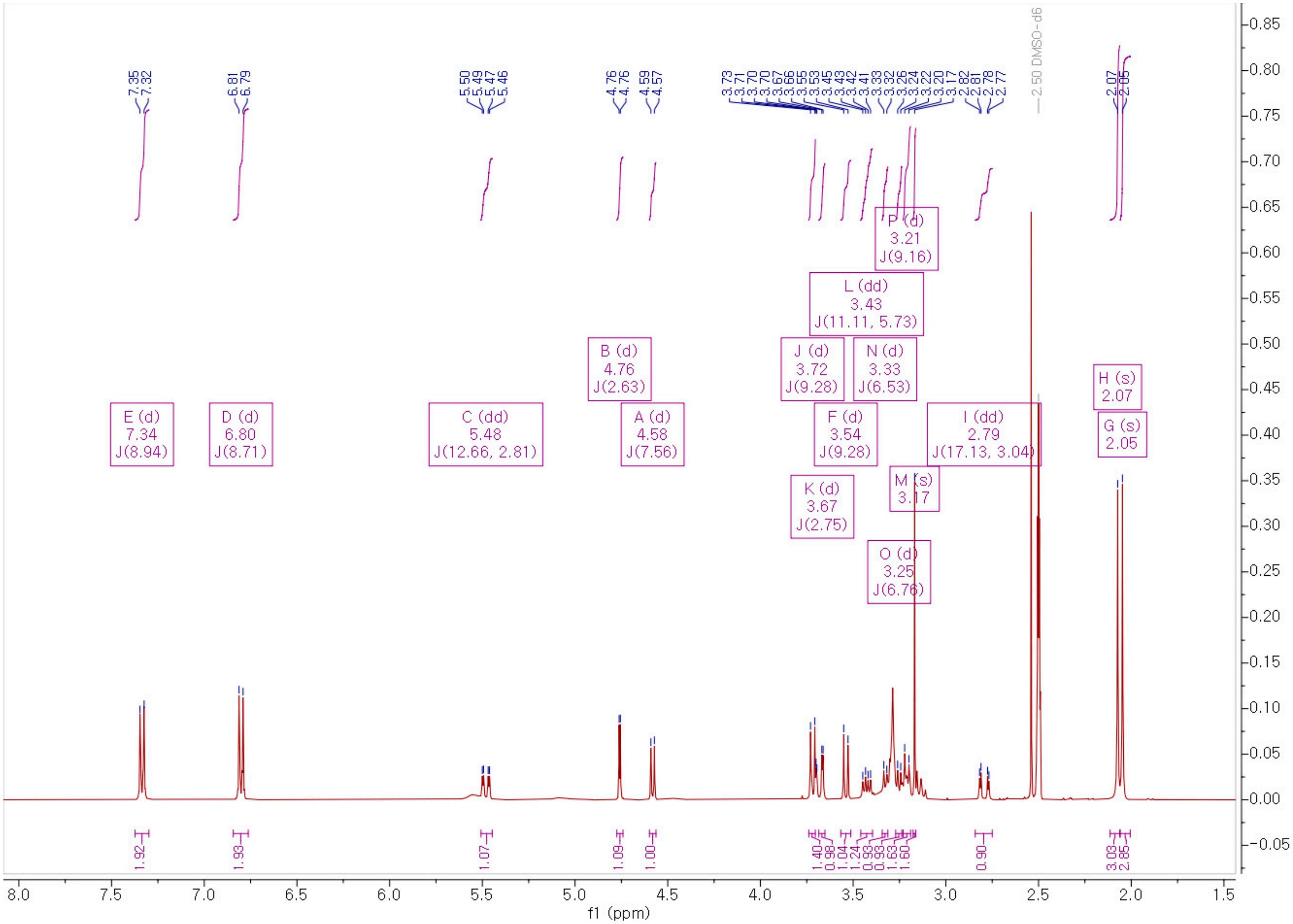

Figure S55. The ^{1}H NMR spectrum of compound **40** (DMSO-d_6, 400 MHz).

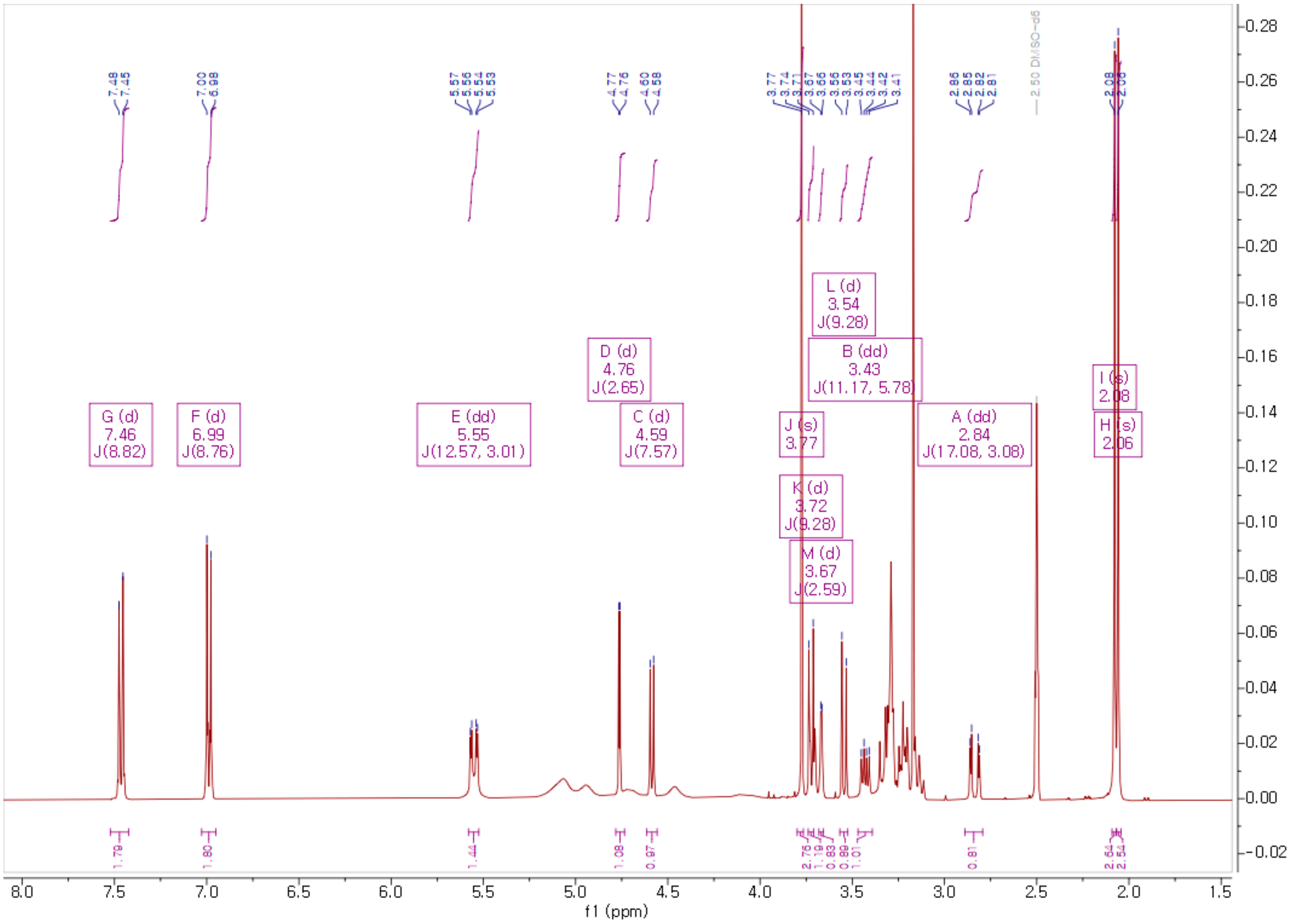

Figure S56. The ^{1}H NMR spectrum of compound **41** (DMSO-d_6, 400 MHz).

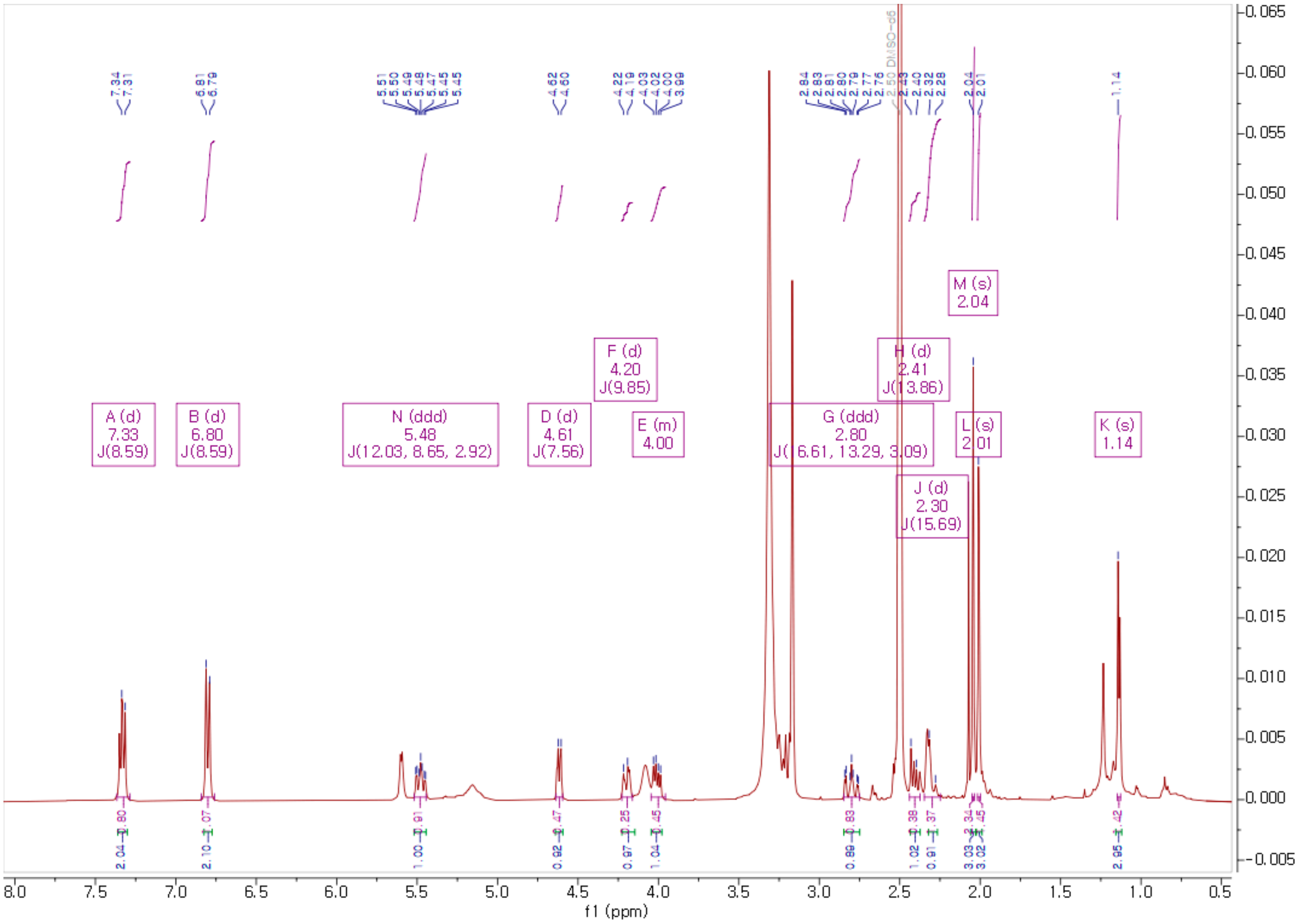

Figure S57. The ^{1}H NMR spectrum of compound **42** (DMSO-d_6, 400 MHz).

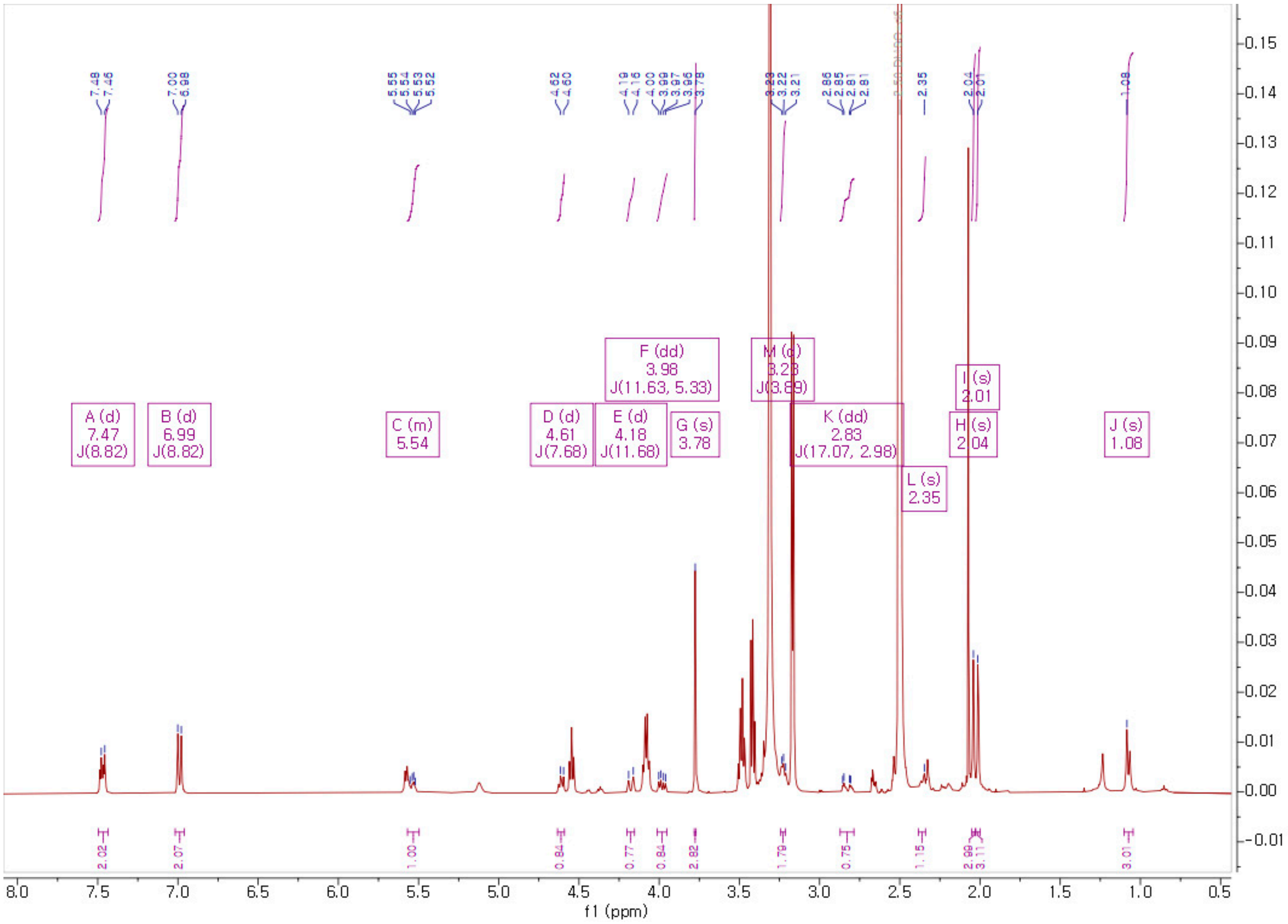

Supplement: Supplementary file 1 [file nutrients-17-01552-s001.zip › nutrients-3561576-supplementary.pdf]
